# The Hippocampus and Entorhinal Cortex Encode the Path and Euclidean Distances to Goals during Navigation

**DOI:** 10.1016/j.cub.2014.05.001

**Published:** 2014-06-16

**Authors:** Lorelei R. Howard, Amir Homayoun Javadi, Yichao Yu, Ravi D. Mill, Laura C. Morrison, Rebecca Knight, Michelle M. Loftus, Laura Staskute, Hugo J. Spiers

**Affiliations:** 1 UCL Institute of Behavioural Neuroscience, Research Department of Experimental Psychology, Division of Psychology and Language Sciences, University College London, London WC1H 0AP, UK; 2 Aging & Cognition Research Group, German Center for Neurodegenerative Diseases (DZNE), 39120 Magdeburg, Germany; 3 UCL Centre for Advanced Biomedical Imaging, University College London, London WC1E 6DD, UK; 4 School of Psychology & Neuroscience, University of St. Andrews, Fife KY16 9JP, UK; 5 Department of Psychology, University of Hertfordshire, Hertfordshire AL10 9AB, UK

## Abstract

**Background:**

Despite decades of research on spatial memory, we know surprisingly little about how the brain guides navigation to goals. While some models argue that vectors are represented for navigational guidance, other models postulate that the future path is computed. Although the hippocampal formation has been implicated in processing spatial goal information, it remains unclear whether this region processes path- or vector-related information.

**Results:**

We report neuroimaging data collected from subjects navigating London’s Soho district; these data reveal that both the path distance and the Euclidean distance to the goal are encoded by the medial temporal lobe during navigation. While activity in the posterior hippocampus was sensitive to the distance along the path, activity in the entorhinal cortex was correlated with the Euclidean distance component of a vector to the goal. During travel periods, posterior hippocampal activity increased as the path to the goal became longer, but at decision points, activity in this region increased as the path to the goal became closer and more direct. Importantly, sensitivity to the distance was abolished in these brain areas when travel was guided by external cues.

**Conclusions:**

The results indicate that the hippocampal formation contains representations of both the Euclidean distance and the path distance to goals during navigation. These findings argue that the hippocampal formation houses a flexible guidance system that changes how it represents distance to the goal depending on the fluctuating demands of navigation.

## Highlights

• The hippocampus represents both the path and the Euclidean distances to goals
• Entorhinal activity reflects the change in the Euclidean distance when the goal is set
• The posterior hippocampus represents the future path at different stages en route
• Significant correlations are abolished when travel is guided by external cues

## Graphical Abstract

**Figure:**
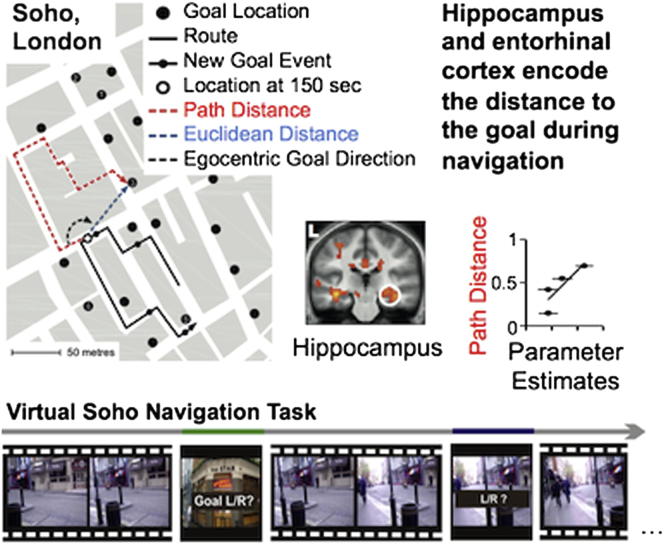

## Teaser

Howard et al. reveal that during the navigation of a simulated real-word environment, hippocampal and entorhinal activity is correlated with the distance to the goal. The posterior hippocampus encodes the path during travel, decision making, and detours. The entorhinal cortex encodes the distance along the vector when the goal is set.

## Introduction

The mammalian brain has developed a remarkable capacity to create an internal map of space and keep track of current heading direction. Evidence of a cognitive map comes from the spatially localized firing of hippocampal “place cells” and entorhinal “grid cells,” which code for an animal’s current position in an environment [1, 2]. “Head direction cells” in companion structures [3] provide a signal for orientation. Despite substantive gains in understanding how these cells support spatial cognition, we know surprisingly little about how the brain uses such information to guide navigation.

While numerous functional MRI (fMRI) studies have explored the neural correlates of navigation [4–16], few have tested predictions from computational models. Such models have mainly used one of two mechanisms for guidance: (1) the straight-line Euclidean distance to the goal is computed as part of a heading vector, allowing shortcuts to be detected [17–21]; and (2) the path to the goal is computed, enabling optimal routes to be selected and dead ends to be avoided [22–27]. These two mechanisms provide divergent predictions about how neural activity will be modulated by the distance to the goal during navigation, but both implicate medial temporal lobe (MTL) structures. Path-processing models can be interpreted as predicting that MTL activity will reflect the distance along the intended path to the goal (path distance) because computational demands will vary with the path distance. By contrast, vector models argue that neurons provide a firing-rate population vector proportional to the Euclidean distance to the goal. Recently, it has been argued that the anterior hippocampus provides a global representation of the environment, whereas the posterior hippocampus contains a fine-grained representation [15, 28]. Thus, it is possible that the anterior and posterior hippocampus contain different representations of the distance to the goal such that the posterior codes the specific regions of space forming the path and the anterior codes more global Euclidean distance information.

To test these predictions, we used fMRI and a novel real-world navigation task in which the Euclidean distance and the path distance to the goal had separable values over time. We found that MTL activity was correlated with both the path distance and the Euclidean distance during navigation and that the relationship between MTL activity and these spatial metrics depended on the task demands at different stages of navigation.

## Results

**Figure 1:**
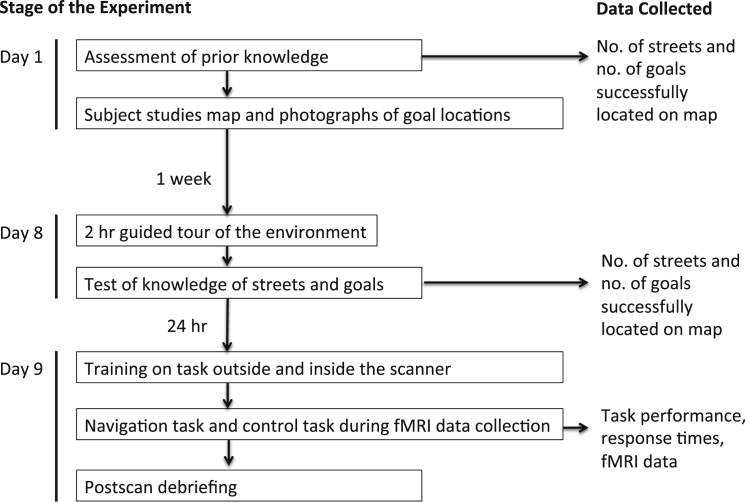
A Flow Chart of the Experimental Protocol Subjects were instructed to spend at least 30 min studying the training material between days 1 and 8. On day 8, all subjects confirmed that they had completed the training material. See Figure S1 for training materials.

**Figure 2:**
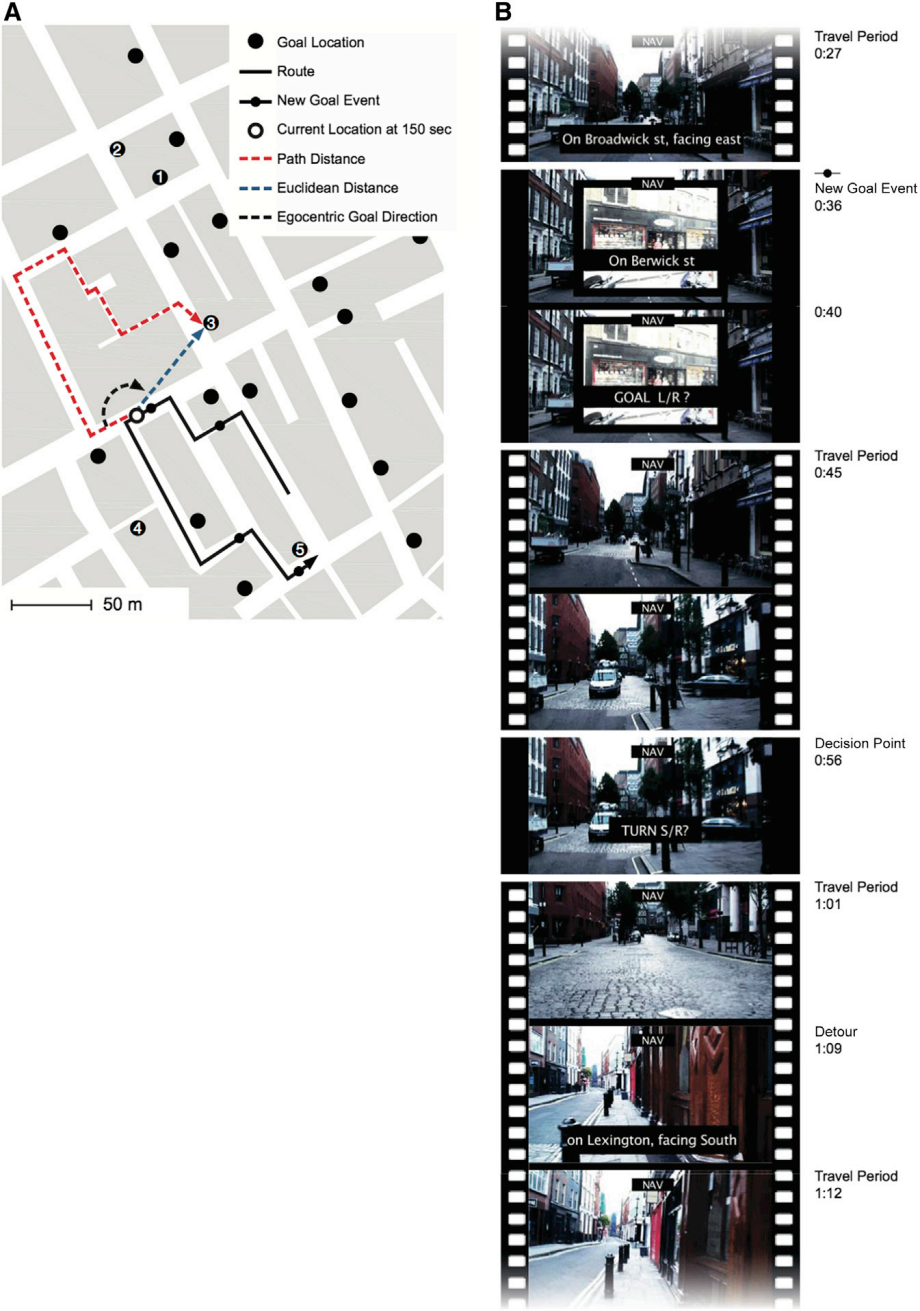
Task (A) Map of the environment (Soho, London). One of the ten routes is shown (black line) with New Goal Events (black circles on route), and their corresponding goal locations (numbered) are marked. The Euclidean distance (blue dashed line), path distance (red dashed line), and egocentric direction (black dashed line) to the goal are plotted for one location on the route. (B) An example sequence of movie frames from a small section of one route in the navigation task. At New Goal Events, subjects were given a new goal to navigate to, and they were required to decide whether that new goal was on the left or right in relation to their current facing direction. In between New Goal Events, movies contained footage of travel along the streets (travel periods) and paused near each street junction (Decision Points), where subjects judged which direction provided the shortest route to the goal. On entry to every street (temporally jittered in relation to Decision Points), the street name and cardinal direction were displayed. Occasionally, forced Detours occurred at street junctions where the movie took a suboptimal path to reach the goal. The control task was similar, but no navigational judgments were required. See Figure S2 for comparisons of activity in navigation and control tasks.

Prior to scanning, subjects learned, via studying maps and an intensive walking tour, the layout of a previously unfamiliar environment: the Soho district in London (Figures 1 and 2; Figure S1, available online). The day after the tour, subjects were scanned while watching ten first-person-view movies of novel routes through the environment. Five of the movies required subjects to make navigational decisions about how to reach goal locations (navigation routes), and the other five required no navigational decision making (control routes). Movies and tasks were counterbalanced across subjects. At the start of each navigation route, subjects were oriented as to where they were, and then shortly after (a period temporally jittered to be between 5 and 13 s), they were shown a goal destination (New Goal Event) and asked to indicate via a button press whether they thought the goal was to their left or right. They then viewed footage in which their viewpoint traversed the street (travel period) until arriving near the junction (Figure 2). At this time point, subjects pressed a button to indicate which direction at the upcoming junction provided the shortest path to the goal (Decision Point), after which the movie continued along the route. Varying the distance between the Decision Point and the junction allowed for a temporal jitter (3–9 s) between the Decision Point and outcome (crossing junction). Subjects were told they could not choose to turn around or walk backward at any point. At the beginning of each new street section, subjects were told which street they were on and the direction they were facing (north, south, east, or west). Routes were predetermined such that they generally followed the optimal route but occasionally required a forced detour (Detours) where the movie traveled along a suboptimal path. Subjects were informed that Detours were only temporary obstructions and would not affect the same junction in the future. The goal being navigated to changed several times (four or five) during each route at additional New Goal Events. In control routes (alternating in order with navigation routes), subjects were instructed to not navigate and to avoid thinking about the locations of goals or the directions to them. Control routes had the identical format to navigation routes, except that at New Goal Events, subjects were asked to indicate by a button press whether or not a drink could be purchased from that goal and were instructed which button to press at Decision Points. The button to press at each Decision Point was based on the optimal answer in the navigation version of that route. All routes ended when the current goal was reached and the text “final destination reached” was displayed with a photograph of the goal. Between routes, a gray screen with a fixation cross appeared for 17 s. See Figures 1 and 2 and the Supplemental Experimental Procedures for further details.

### Behavioral Results

Subjects acquired a detailed spatial knowledge and accurately performed the tasks (Table S1). For navigation routes, mean accuracy was 84.82% (SD = 10.96) at New Goal Events and 79.91% (SD = 13.28) at Decision Points. For control routes, mean accuracy was 95.90% (SD = 5.77) at New Goal Events and 97.63% (SD = 5.74) at Decision Points. Subjects made significantly fewer errors in the control task (F_(1,23)_ = 40.27, p < 0.001). Subjects were both faster to respond and more accurate at Decision Points when the goal was situated closer (in terms of the path distance) and more directly ahead (Table S1). At New Goal Events, we found no relationship between subjects’ performance (accuracy and response time) and the magnitude of the change in any of the spatial parameters (Table S1).

### fMRI Results

fMRI analyses revealed that retrosplenial, parietal, and frontal cortical regions and the cerebellum were significantly more active (at an uncorrected threshold of p < 0.001) during the navigation task blocks, New Goal Events, and Decision Points than during the control task blocks and events (Figure S2; Table S2). Significantly greater right posterior hippocampal activity was also observed during navigation task blocks than during control task blocks (Table S2).

To gain leverage on the spatial computations performed by the brain during navigation, we probed the fMRI data with measures of the Euclidean distance, path distance, and egocentric direction to the goal. First, we explored our a priori predictions (see Supplemental Experimental Procedures) during New Goal Events, Decision Points, Detours, and Travel Period Events (events sampled during travel periods at the temporal midway point between the time points of the other events, for both navigation and control routes). Second, on finding significant effects, we examined whether similar responses occurred in the control routes. Third, where responses were specific to navigation, we tested whether there was a significantly greater effect in navigation routes than in control routes. Finally, we examined whether these responses were significantly greater during certain event types than others and whether responses were significantly more correlated with one parameter than with others.

### Both Euclidean and Path Distances Are Tracked by the Hippocampus during Travel

**Figure 3:**
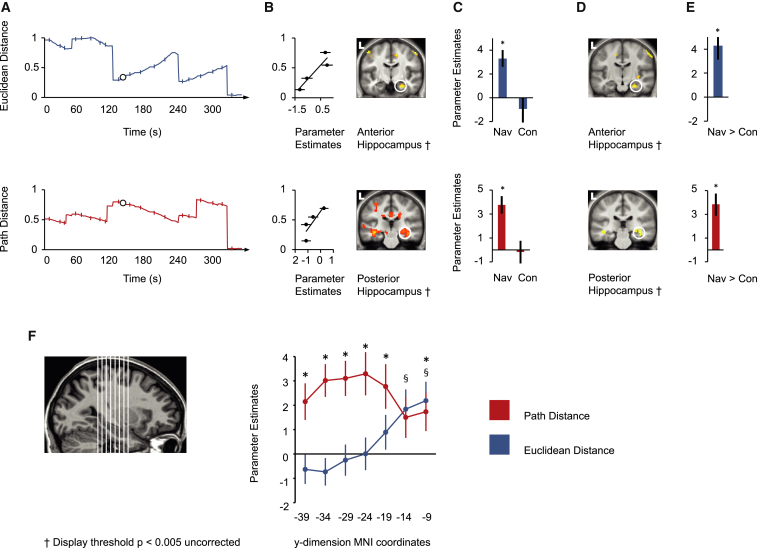
Hippocampal Activity Positively Correlates with Euclidean and Path Distances to the Goal during Travel Periods in Navigation Tasks (A) Top: the normalized Euclidean distance to the goal is plotted against time for the route shown in Figure 2A. Bottom: the normalized path distance to the goal is plotted against time for the route shown in Figure 2A. Normalization was with respect to the maximum over all routes. On both plots, the circle indicates the time point at 150 s (marked in Figure 2A), and Travel Period Events are indicated with bisecting lines. (B) Top: right anterior hippocampal activity correlated significantly with the Euclidean distance to the goal during navigation. Bottom: right posterior hippocampal activity correlated significantly with the path distance to the goal during navigation. Accompanying scatter plots show the normalized Euclidean distance (top) and path distance (below; separated into four levels) plotted against parameter estimates at the peak voxel for these regions. Note that these plots were not used for statistical inference (which was carried out within the statistical parametric mapping framework) and are shown solely for illustrative purposes. The following abbreviation is used: L, left. (C) Top: the parameter estimates for the peak voxel in the right anterior hippocampus in the navigation (Nav) condition are plotted for navigation and control (Con) conditions. Bottom: the parameter estimates for the peak voxel in the right posterior hippocampus in the navigation condition are plotted for the navigation and control conditions. Asterisks indicate significance at a threshold of p < 0.05 (family-wise error was corrected for a priori regions of interest). See Figure S3 for parameter estimates in all ROIs. (D) Top: right anterior hippocampal activity correlated significantly more positively with the Euclidean distance to the goal during navigation conditions than during control conditions. Bottom: right posterior hippocampal activity correlated significantly more positively with the path distance to the goal during navigation conditions than during control conditions. The following abbreviation is used: L, left. (E) Top: the bar graph shows the parameter estimate for the peak voxel in the right anterior hippocampus in the navigation > control contrast for the Euclidean distance. Bottom: the bar graph shows the parameter estimate for the peak voxel in the right posterior hippocampus in the navigation > control contrast for the path distance. Asterisks indicate significance at a threshold of p < 0.05 (family-wise error was corrected for a priori regions of interest). (F) Left: illustration of the seven sections through the longitudinal axis of the hippocampus. Middle: the parameter estimates of the parametric response to Euclidean and path distances for each of the seven sections (numbers on the x axis indicate the middle MNI y coordinate of each ROI) during Travel Period Events in navigation tasks. These parameter estimates were not used for detecting effects of interest but rather for characterizing the response post hoc. § symbols indicate a significant Euclidean distance, and asterisks indicate a significant path distance in relation to zero at p < 0.05 (see Table S5). Error bars in (B), (C), (E), and (F) denote the SEM.

During Travel Period Events in the navigation routes, activity in the posterior hippocampus was significantly positively correlated with the path distance to the goal (i.e., more active at larger distances, see Figures 3A and 3B; Table S2). However, at the same time points, activity in the anterior hippocampus was significantly positively correlated with the Euclidean distance to the goal (Figures 3A and 3B; Table S2). Significant correlations were also present when we downsampled the Travel Period Events to remove 25% of the events in which the Euclidean and path distances were most correlated (Table S2). A region-of-interest (ROI)-based analysis of the hippocampal longitudinal axis revealed that whereas the posterior and mid hippocampus were specifically correlated with the path distance to the goal (but not the Euclidean distance), the anterior hippocampus was not specific to the Euclidean distance (Figure 3F; Figure S3). This was further confirmed by direct contrasts between parameters (Table S3).

Models assume that the guidance system is under volitional goal-directed control rather than automatic control. Our data support this view. No significant correlation between hippocampal activity and the distance (either Euclidean or path) to the goal was observed during the Travel Period Events in the control routes. Furthermore, hippocampal activity was also significantly more positively correlated with distance measures in these events during navigation routes than during control routes (Figures 3C–3E; Table S2). Because route (1–5 versus 6–10) and task (navigation versus control) were counterbalanced across subjects, significant correlations could not have been purely stimulus driven. Nor were the correlations with the distance to the goal confounded with the time elapsed or distance traveled since the route began (Table S2).

Beyond the MTL, at a corrected threshold, the anterior cingulate was the only region that showed a significant correlation with distance in any of our event types, specifically (1) during navigation routes and (2) more in navigation routes than in control routes. It was positively correlated with the path distance to the goal during Travel Period Events in navigation routes and significantly more positively correlated in navigation routes than in control routes (Figure S4; Table S2).

### Egocentric Goal Direction Is Tracked by the Posterior Parietal Cortex during Travel

Activity in the MTL during travel periods was not correlated with egocentric direction to the goal or the interaction between this directional measure and distance (either Euclidean or path) to the goal. However, consistent with prior observations [10], during navigation routes, activity in the superior posterior parietal cortex was significantly positively correlated with the egocentric direction to the goal (i.e., the greater the angle between the current heading and the heading directly to the goal, the greater the activity [Figures S3 and S4; Table S2]). No such correlation was observed during Travel Period Events in the control routes. However, although the correlation was more positive during the Travel Period Events in navigation routes than in control routes, it was not significantly more positive (Table S2). We also observed lateral posterior parietal activity negatively correlated with the egocentric direction to the goal (Figure S4; Table S2); however, this did not survive at corrected thresholds.

### Posterior Hippocampal Activity Increases with Proximity and Orientation toward the Goal at Decision Points

**Figure 4:**
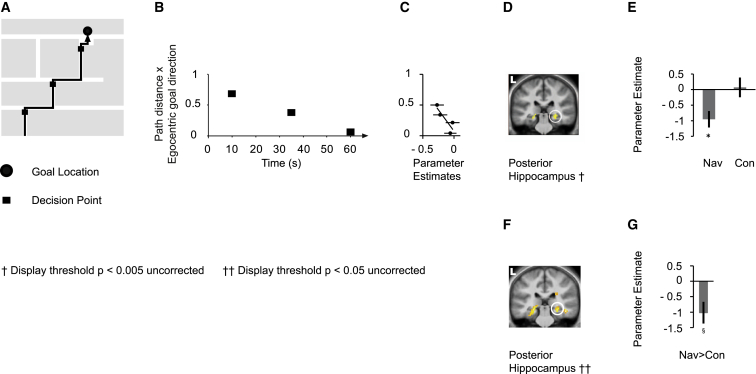
Posterior Hippocampal Activity Negatively Correlates with the Distance and Direction to the Goal during Decision Points in Navigation Tasks (A) Illustrative map with part of a route (black line) to a goal location (black circle) and Decision Points (black squares). (B) The parameter “normalized path distance to the goal × egocentric goal direction” (PD×EGD) at the three Decision Points from the example route in (A) is plotted against time. (C) Normalized PD×EGD separated into four levels is plotted against parameter estimates at the peak voxel of the posterior right hippocampus. Note that the scatter plot was not used for statistical inference (which was carried out within the SPM framework) and is shown solely for illustrative purposes. (D) Right posterior hippocampal activity correlated significantly negatively with PD×EGD during Decision Points in navigation. The following abbreviation is used: L, left. See Figure S4 for other coronal sections with this and other contrasts. (E) The parameter estimates for the peak voxel in the right posterior hippocampus in the navigation condition are plotted for navigation (Nav) and control (Con) conditions. Asterisks indicate significance at a threshold of p < 0.05 (family-wise error was corrected for a priori regions of interest). (F) Right posterior hippocampal activity correlated significantly more negatively with PD×EGD during navigation routes than during control routes. The following abbreviation is used: L, left. (G) The bar graph shows the parameter estimate for the peak voxel in the right posterior hippocampus in the navigation > control contrast for PD×EGD. § symbols indicate significance at a threshold of p < 0.005 (uncorrected). Errors bars in (C), (E), and (G) denote the SEM.

Hippocampal activity did not correlate with the Euclidean or path distance at Decision Points. However, because subjects responded faster, and more accurately, when the path distance was shorter and the goal was ahead of them (Table S1), we explored whether hippocampal activity was related to an interaction between the path distance and the egocentric goal direction by examining the response to the multiplication of these two variables (Figure 4). We also included response time in our analysis. We found that posterior hippocampal activity increased the closer, and more directly ahead, the goal lay (Figures 4B–4D; Figures S3 and S4; Table S2). Activity increased such that when subjects were close to and facing the goal, activity was similar to that during the fixation period between routes. No significant correlation with the path distance by egocentric goal direction was observed in the posterior hippocampus in control routes, and the correlation between this parameter and posterior hippocampal activity was significantly more negative in navigation routes than in control routes (Figures 4E–4G; Table S2). The significant correlation in navigation routes was independent of response time, which did not modulate MTL activity. The number of options at Decision Points (two or three) also had no impact on MTL activity (the path distance did not differ between these two types of Decision Points [t_(51)_ = 0.04, p = 0.97]).

### Entorhinal Activity Scales with the Change in the Euclidean Distance at New Goal Events

**Figure 5:**
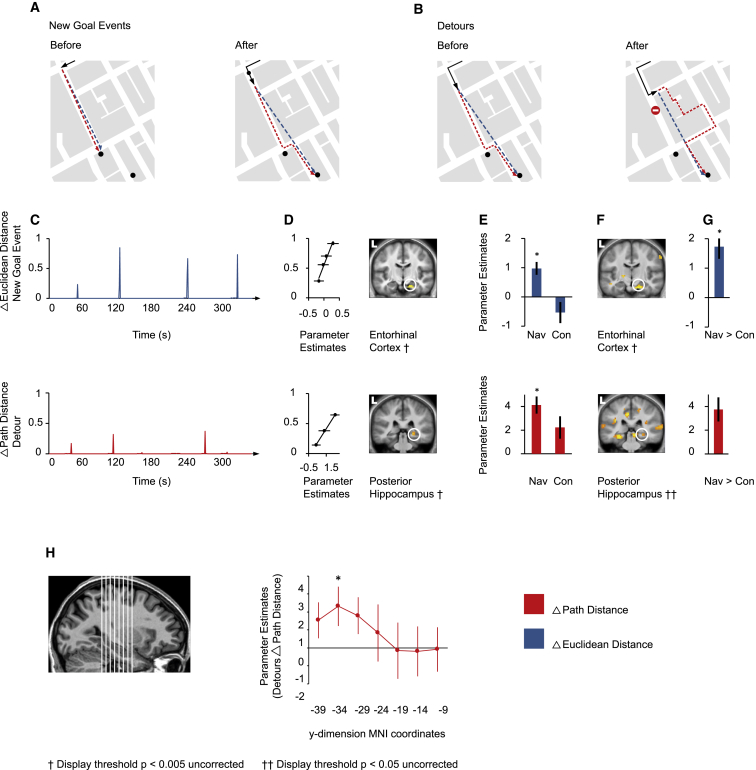
Entorhinal Activity and Posterior Hippocampal Activity Positively Correlate with the Change in the Euclidean Distance to the Goal during New Goal Events and the Change in the Path Distance to the Goal during Detours, Respectively (A) Illustrative example of how the Euclidean and path distances to the goal can change at New Goal Events. (B) Illustrative example of how the path distance to the goal can change at Detours. The “no entry” sign marks the Detour, but no marker was presented in the movie. (C) Top: the normalized differential (Δ) of the Euclidean distance to the goal at New Goal Events is plotted against time for the route shown in Figure 2A. Bottom: the normalized differential (Δ) of the path distance to the goal at Detours is plotted against time for the route shown in Figure 2A. Normalization was with respect to the maximum over all routes. (D) Top: right entorhinal activity significantly correlated with the Δ Euclidean distance to the goal during New Goal Events in navigation. Bottom: right posterior hippocampal activity significantly correlated with the Δ path distance during Detours in navigation. Accompanying scatter plots show the normalized Δ Euclidean distance (top) and the Δ path distance (bottom) (separated into four and three levels, respectively) plotted against parameter estimates at the peak voxel for these regions. Note that these plots were not used for statistical inference (which was carried out within the SPM framework) and are shown solely for illustrative purposes. See Figure S5 for a display of results on other coronal sections. The following abbreviation is used: L, left. (E) Top: the parameter estimates for the peak voxel in the entorhinal cortex in the navigation condition are plotted for navigation (Nav) and control (Con) conditions. Bottom: the parameter estimates for the peak voxel in the posterior hippocampus in the navigation condition are plotted for the navigation and control conditions. Asterisks indicate significance at a threshold of p < 0.05 (family-wise error was corrected for a priori regions of interest). (F) Top: right entorhinal activity correlated significantly more positively with the Δ Euclidean distance to the goal at New Goal Events during navigation routes than during control routes. Bottom: right posterior hippocampal activity correlated more positively with the Δ path distance at Detours during navigation routes than during control routes, but not significantly. (G) Top: the bar graph shows the parameter estimate for the peak voxel in the right entorhinal cortex in the navigation > control contrast for the Δ Euclidean distance to the goal at New Goal Events. Bottom: the bar graph shows the parameter estimate for the peak voxel in the right posterior hippocampus in the navigation > control contrast for the Δ path distance at Detours. Asterisks indicate significance at a threshold of p < 0.05 (family-wise error was corrected for a priori regions of interest). (H) Left: illustration of seven sections through the longitudinal axis of the hippocampus (these were used for plotting the parameter estimates in the middle panel). Middle: parameter estimates of the parametric response to the Δ path distance at Detours during navigation for each of the seven hippocampal ROIs (numbers on the x axis indicate the middle MNI y coordinate of each ROI). These parameter estimates were not used for detecting effects of interest but rather for characterizing the response post hoc. Asterisks indicate significance relative to zero at p < 0.05 (see Table S5). Error bars in (D), (E), (G), and (H) denote the SEM.

At New Goal Events, the distance to the goal changed abruptly (Figures 5A and 5C). For navigation routes, we found that the greater the change in the Euclidean distance (but not the path distance) at these time points, the greater the evoked response in the right entorhinal cortex (Figure 5D; Figures S3 and S5; Table S2). At New Goal Events, the goal could move to a location that was closer to or farther from the subject (in terms of both path and Euclidean distances). We found no difference in MTL activity associated with New Goal Events either when the new goal was located closer to the subject or when it was located farther away (for both distance types). Notably, increases and decreases in either the Euclidean or path distance for these two types of New Goal Events were not significantly different in magnitude (Euclidean distance: t_(41)_ = 0.54, p = 0.59; path distance: t_(41)_ = 1.96, p = 0.056). No significant correlation with the change in the Euclidean distance was observed in the entorhinal cortex in control routes, and the correlation between entorhinal activity and this parameter was significantly more positive in the New Goal Events in navigation routes than in control routes (Figures 5E–5G; Table S2). The correlation between entorhinal activity and the change in the Euclidean distance during New Goal Events in navigation routes was also significantly more positive than the correlation with the change in the path distance during New Goal Events in navigation routes (Table S3). Finally, we also explored the MTL response to the distance (path and Euclidean) to the new goal at New Goal Events and found no significant correlation between MTL activity and either type of distance (Figure S3).

### Right Posterior Hippocampal Activity Reflects the Amount of Change in the Path Distance at Detours

At Detours, subjects were unable to proceed along the optimal path and thus had to derive an alternative route to the goal. At these events, the path distance to the goal increased abruptly and by varying amounts (Figures 5B and 5C). Our data show a dissociation between prefrontal and MTL responses at Detours. Consistent with prior studies [6, 29], prefrontal regions, but not MTL regions, were significantly more active at Detours than during optimal route progression at junctions or events in control routes (Figure S2; Table S2). However, we found that right posterior hippocampal, but not prefrontal, activity was positively correlated with the magnitude of change in the path distance during Detours (i.e., Detours that added a large amount of distance evoked more posterior hippocampal activity than did Detours that added a small distance [Figures 5D and 5H; Figures S3 and S5; Table S2]). No equivalent significant correlation was present at corresponding Detour events in the control movies. Although the correlation between the change in the path distance and hippocampal activity at Detours was greater in navigation routes than in control routes, this difference did not reach significance (Figures 5E–5G; Table S2). See Table 1 for a summary of these and other results.

### Comparison of Correlations with Spatial Parameters across Different Event Types

We found that all correlations between MTL activity and the distance to the goal were specific to each event type (Table S4). For example, the correlation between posterior hippocampal activity and the path distance during Travel Period Events was significantly more positive during Travel Period Events than during Decision Points or New Goal Events. The posterior parietal response to egocentric goal direction was not significantly more positive during Travel Period Events than during other events (Table S4).

### Analysis of the Mean Response in ROIs

When we used an alternative approach of examining the mean response in our ROIs, we found a small number of differences from our statistical parametric mapping (SPM) analysis (Figure S3; Table S6). Examining the Euclidean distance to the goal during Travel Period Events, we found that although there was no significant cluster in the right entorhinal cortex in SPM, our ROI analysis revealed a significant correlation. A similar pattern was found in the left posterior parietal cortex for the egocentric goal direction to the new goal at New Goal Events.

## Discussion

Using a novel real-world task, we explored how the brain dynamically encodes the distance to goals during navigation. Our results provide support for both vector- and path-processing accounts of navigational guidance [17–26] and give insight into the precise navigation stages during which the different regions of the MTL process the distance to future goals. In summary, we found that whereas posterior hippocampal activity was related to the path distance to the goal (during travel, decision making, and forced detours), anterior hippocampal activity (during travel) and entorhinal activity (during the processing of new goals) reflected the Euclidean distance to the goal. These responses were relatively specific to these time periods, and with the exception of anterior hippocampal activity, responses were relatively selective to one type of distance.

Our study provides a number of advances over previous fMRI studies exploring representations of distance in the MTL [10, 16, 30, 31]. First, the absence of significant effects in our control routes, and the observation of significantly stronger activity during navigation routes than during control routes in the majority of analyses, indicates that simply being led along a path to a goal is insufficient to engage the MTL in processing the distance. Rather, our data are consistent with the view that distance-to-goal coding requires active navigation based on long-term memory of the environment. Second, while the visual properties of the stimuli and their temporal dynamics might have driven the effects in prior studies [10, 16, 30, 31], we show that this was not the case in our study because task and route were counterbalanced. Finally, the fact that we altered the distance to the goal sporadically at time points (Detours and New Goal Events) along the route shows that the MTL activity correlated with the distance was not simply a function of the time elapsed or distance traveled.

These findings advance our understanding of navigational guidance systems in several ways. Whereas many models propose that the brain processes either the path [24–27] or the Euclidean [17–21] distance component of a vector to the goal, we reveal that both representations are actively deployed during different time windows and by different MTL regions. While it is important to acknowledge that the responses we observed show modulation over time rather than categorical on and off responses, our results are consistent with the following explanation: during the initiation of navigation, when the spatial relationship to the goal must be established, information related to the Euclidean distance along the vector is processed, and when path choice is required at Decision Points or a detour along a new route is required, information related to the path distance is represented. Although such results are consistent with models in which both vector and path search mechanisms are used [23], no current model captures the dynamic pattern of distance representations we observed. Thus, we provide much needed empirical data for the development of future models.

Previous studies reporting MTL activity correlated with the distance to goal have provided apparently contradictory reports. While some studies have found that activity increases as the goal becomes farther away [10, 31], others have reported that activity increases as the goal becomes closer [16, 30, 32]. These prior studies did not dissect the operational stages during navigation, nor did they isolate the type of distance that might have been represented. By doing so, we found that both profiles of response can occur at different stages of a single journey and that different types of distances can be represented in different time windows. A possible determinant of the activity profile may be whether subjects had to update their spatial position or decide which path to take. In our study, and others [10, 31], activity increased as the distance during periods of spatial updating (e.g., Travel Period Events) became longer. By contrast, in other studies [16, 30], hippocampal activity increased as the distance to the goal became shorter during decision making about which path or direction to take. Our findings extend prior work by revealing that the proximity to the goal along the path (but not the Euclidean) distance, combined with the direction to the goal, modulates hippocampal activity at Decision Points. Previous studies reporting that hippocampal activity increased with proximity to the goal did not include goal direction in their analysis [16, 30]; thus, it is possible that an interaction between distance and direction was present, but not detected. While several models predict that the path to the goal is represented in the hippocampal population activity [22, 24–27] or that activity changes with goal proximity [17, 18, 20], none argue that activity reflects both distance and direction. Given that estimates of the distance along a path have been found to be biased by the number of junctions and turns along the path [33], it is possible that facing away from the goal might increase the subject’s internal estimate of the distance. If so, our combined measure of distance and direction may more accurately reflect the subjects’ estimate of the distance than the distance we measured from geospatial data. Exploring this will require further research.

While our primary focus was the MTL, we found responses in other regions thought to be important for navigation. Consistent with prior research [5, 11, 16, 34], we observed greater activity in parietal and retrosplenial cortices during navigation tasks (route blocks, New Goal Events, and Decision Points) than during control tasks. Of these regions, the posterior parietal cortex showed a correlation with the egocentric direction to the goal, consistent with a similar previous report [10] and a role in egocentric processing [35]. It is not clear why parietal activity increases the more the goal lies behind the subject. It is possible that landmarks and geometry in the current field of view make it easier to determine the direction to a goal ahead of the subject, and thus by comparison, make it more demanding to track goals located behind. Alternatively, increased parietal activity may suggest that subjects pay greater attention to direction the more the goal lies behind them.

Our results inform the debated specialization of function in the anterior and posterior hippocampus [28, 36, 37]. Posterior hippocampal activity was consistently correlated with the path distance to the goal. This region is the homolog of the rodent hippocampal dorsal (septal) pole, which contains place cells, representing small regions of space with their “place fields” [38], and is thus suited to the fine-grain coding of space along precise paths [28]. Moreover, such cells can exhibit “forward sweeps” during travel [39] and “replay” of locations along the path ahead prior to travel [40], plausibly recruiting more cells the longer the future path, leading to a predicted positive correlation between the length of the path and hippocampal activity. While responses during Travel Period Events and Detours are consistent with this prediction, our response at Decision Points is the opposite of this prediction. Thus, while our data consistently indicate that the posterior hippocampus processes information about the path, it does not appear to do so in a manner directly predicted from “preplay.” Greater integration of rodent and human neural recording methods would be useful for gaining traction on this issue.

Our observed anterior hippocampal activity tracking the distance to the goal during travel periods is consistent with a role in spatial updating [13, 31, 41–43]. If human anterior hippocampal cells, like those of rodents [38], have broad spatial tuning, it would make them suited to extracting global environmental information rather than precise paths [28]. Similarly, the spatially extensive repeating grid-like firing of entorhinal grid cells may make them ideal for computing vectors rather than paths [19, 21, 23]. Our observation of a Euclidean-based code in the right entorhinal cortex is consistent with the finding that the same region codes the Euclidean distance to the goal in London taxi drivers navigating a simulation of London [10]. We found that the entorhinal cortex was equally active for increases and decreases in the Euclidean distance, indicating that resetting the distance rather than purely extending it may drive the response. It is possible that the entorhinal cortex is driven by resetting because it may be more computationally demanding to make large alterations in the representation of the distance than to make small changes. Alternatively, another explanation, provided by Morgan et al. [31], is that this response is driven by a repetition-suppression effect. According to this view, the activity is maximal when the change in the distance is large because it provides the least overlap in the regional representation of the distance.

In this study, we separated path and Euclidean distances. Future studies will be required for dissecting the path distance from other variables. Two such variables are “time to reach the goal” and “reward expectation.” While our analysis revealed that time elapsed was not correlated with hippocampal activity, it is possible that correlates of the path distance rather than purely the distance relate to the estimated time to the goal. Similarly, because reaching a goal is rewarding and the likelihood of this increases with proximity along the path, the path distance and reward expectation are related. Manipulating travel speed, travel costs, and reward outcomes may help separate distance, time, and reward expectation. This would help clarify whether the anterior cingulate activity observed to correlate with the path distance is related to reward expectation. Such a prediction is based on evidence that this region processes progress toward goals [44] and the probability of obtaining a reward [45].

Here, we examined navigation in a recently learned environment. In future research, it will be useful to compare how distance is represented in recently learned and remotely learned environments. It is possible that in remotely learned environments, the distance to the goal is represented by cortical regions rather than the hippocampus [46, 47] and that the type of distance represented changes with familiarity of the environment.

## Figures and Tables

**Table 1:** Summary of Significant Effects with Parametric Measures in Navigation Routes

Event Type	Brain Region			
	Anterior Hippocampus	Posterior Hippocampus	Entorhinal Cortex	Posterior Parietal Cortex
Travel Period Events	+ ED	+ PD	NS	+ EGD
Decision Points	NS	− PD×EGD	NS	NS
New Goal Events	NS	NS	+ ΔED	NS
Detours	NS	+ ΔPD	NS	NS

Abbreviations are as follows: +, positive correlation; −, negative correlation; Δ, change in the parameter; ED, Euclidean distance; EGD, egocentric goal direction; NS, not significant; and PD, path distance. See Figure S3 for the parameter estimates for each parameter, brain region, and event type and Table S6 for the results of an analysis of the mean response in each ROI.

## Supplemental Information

Document S1. Figures S1–S5, Tables S1 and S3–S6, and Supplemental Experimental Procedures

Table S2. Montreal Neurological Institute Coordinates and *Z* Scores for Peak Voxels

Document S2. Article plus Supplemental Information
