# Supplementary material for: The Hippocampus and Entorhinal Cortex Encode the Path and Euclidean Distances to Goals during Navigation
Source: Curr Biol. 2014 Jun 16;24(12):1331–40. doi: 10.1016/j.cub.2014.05.001 (PMC4062938; doi:10.1016/j.cub.2014.05.001)
Supplement: Document S1. Figures S1–S5, Tables S1 and S3–S6, and Supplemental Experimental Procedures [file mmc1.pdf]

Current Biology, Volume *24*

Supplemental Information

The Hippocampus and Entorhinal Cortex Encode the Path and Euclidean Distances to Goals during Navigation

Lorelei R. Howard, Amir H. Javadi, Yichao Yu, Ravi D. Mill, Laura C. Morrison, Rebecca Knight, Michelle M. Loftus, Laura Staskute, and Hugo J. Spiers

Supplemental Figures

A

a) Name as many of the streets in this area as you can:

1. Oxford St
2.____________________________
3.____________________________
4.____________________________
5.____________________________
6.____________________________
7.____________________________
8.____________________________
9.____________________________
10.____________________________
11.____________________________
12.____________________________
13.____________________________
14.____________________________
15.____________________________
16.____________________________
17.____________________________
18.____________________________
19.____________________________
20.____________________________
21.____________________________
22.____________________________
23.____________________________
24.____________________________
25.____________________________
26.____________________________
27.____________________________
28.____________________________
29.____________________________
30.____________________________
31.____________________________
32.____________________________
33.____________________________
34.____________________________
35.____________________________

Name:____________________________

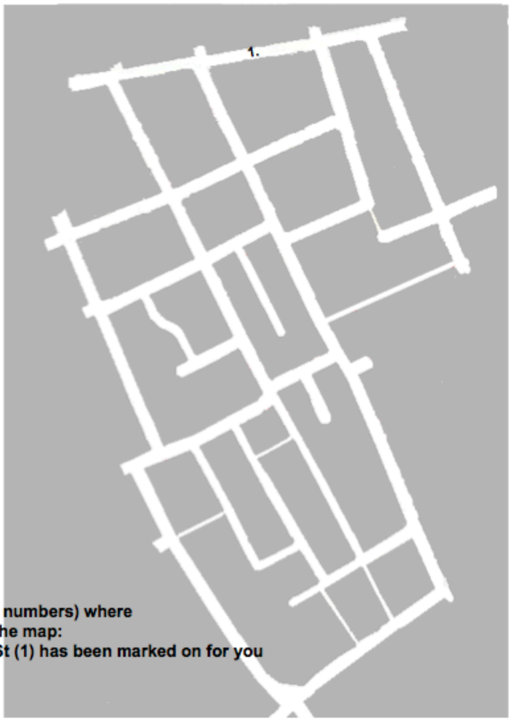

b) Indicate (using the numbers) where these streets are on the map: for example, Oxford St (1) has been marked on for you

a) Please indicate (circle the number) whether any of these places are familiar to you: (i.e., have you walked past them?)

Place

1. 33 Broadwick
2. Calumet Photography
3. Floridita
4. Freedom Bar
5. IMLI Indian Tapas
6. Ingestre Court
7. Let's Fill This Town With Artists
8. Nicholas Wine Shop (Berwick St store)
9. Number One Salon
10. Papaya Cafe
11. Pierre Victoire
12. Refuel
13. Self-Sacrifice
14. Silk Society
15. Sir Tom Baker
16. Sister Ray Records
17. Soho Screening rooms
18. Somerfield (Berwick St store)
19. Star Cafe
20. Tequila Marketing
21. Westminster Kingsway College
22. Yauatcha Restaurant
23. Lo Profile Cafe

b) Indicate (using the dots and numbers) where you think the places you were familiar with are on the map:

Name:____________________________

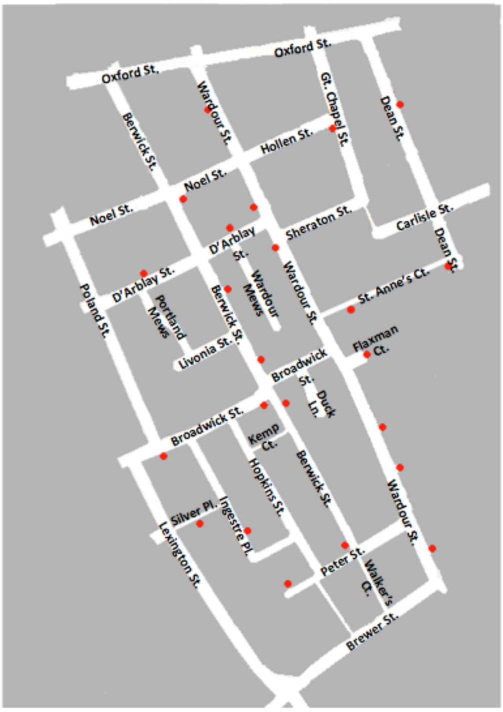

B

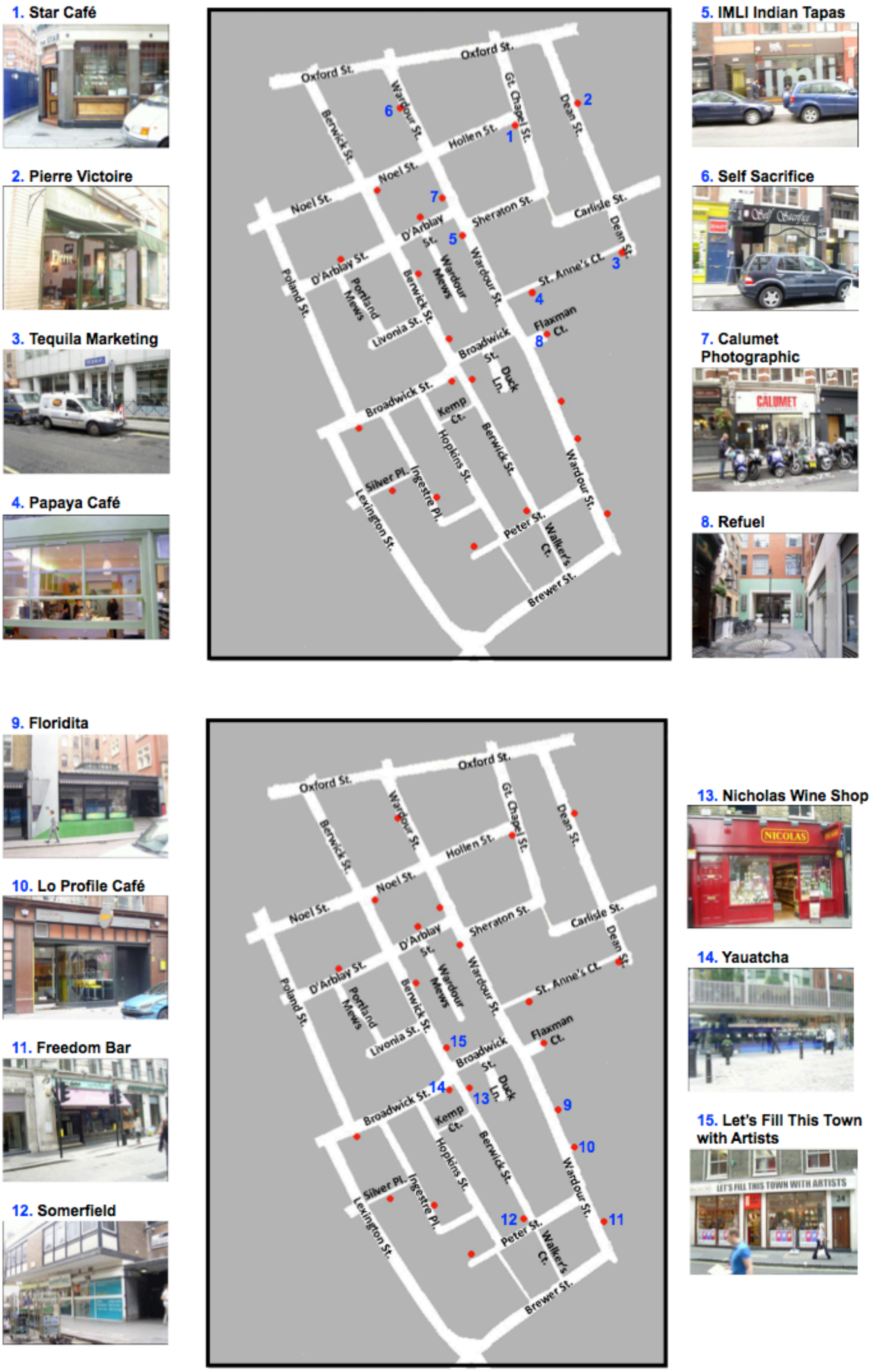
1. Star Café
2. Pierre Victoire
3. Tequila Marketing
4. Papaya Café
5. IMLI Indian Tapas
6. Self Sacrifice
7. Calumet Photographic
8. Refuel
9. Floridita
10. Lo Profile Café
11. Freedom Bar
12. Somerfield
13. Nicholas Wine Shop
14. Yauatcha
15. Let's Fill This Town with Artists
Oxford St.
Berwick St.
Wardour St.
Gt. Chapel St.
Dean St.
Hollen St.
Noel St.
Sheraton St.
Carlisle St.
D'Arblay St.
Poland St.
Portland Mews
Wardour Mews
St. Anne's Ct.
Livonia St.
Broadwick St.
Flaxman Ct.
Duck Ln.
Kemp Ct.
Hopkins St.
Silver Pl.
Ingestre Pl.
Lexington St.
Peter St.
Walker's Ct.
Brewer St.
CALUMET
NICOLAS
LET'S FILL THIS TOWN WITH ARTISTS

B (Continued)

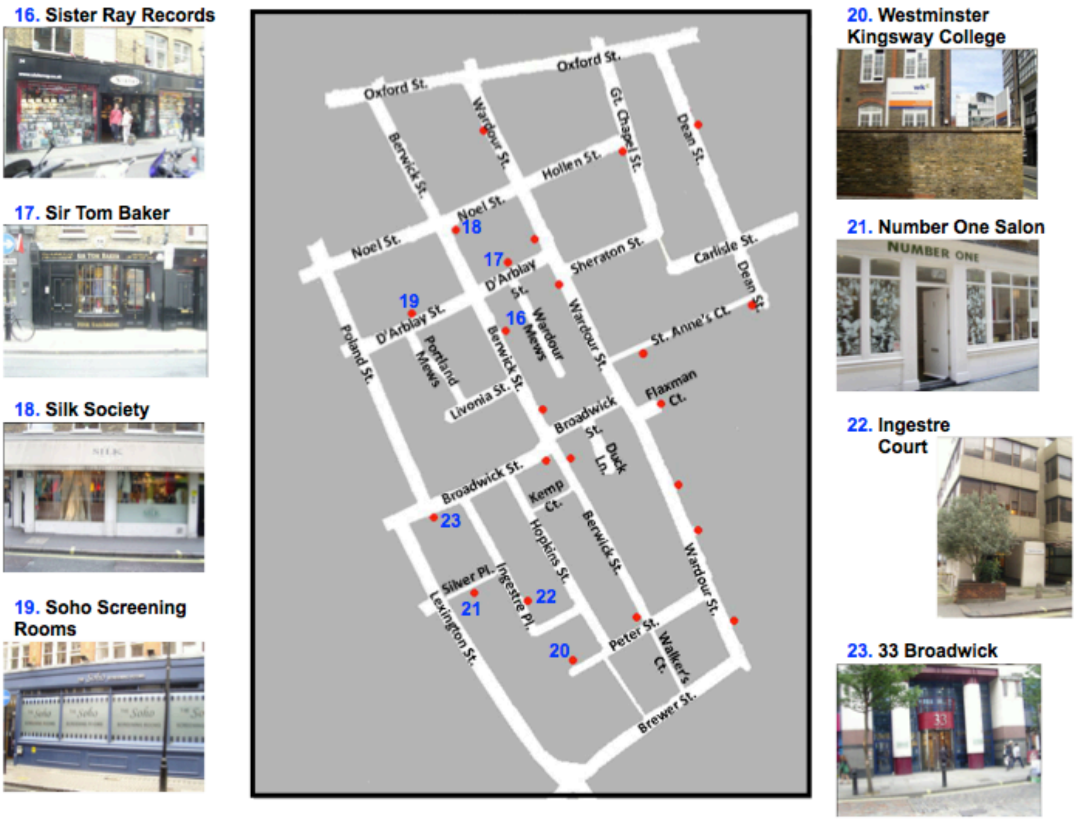

Figure S1. Environmental Knowledge Assessment and Training Material. (**A**) Environmental knowledge assessment. Top: Image from testing material used to test street name knowledge before and after training. Bottom: Image from testing material used to test goal location knowledge before and after training. (**B**) Goal location training material. This material was used to allow subjects to learn about the location of the various goal locations. Note that goal numbers in this material were unrelated to goal location presentation order during New Goal Events during fMRI scanning. Start location material was similar to these but indicated viewpoints at the 10 locations where each route would start.

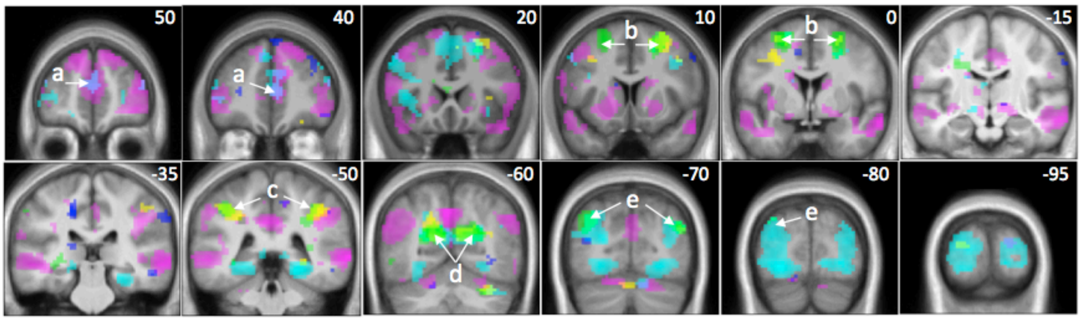

- Tasks (Nav > Con)
- Travel Period Events (Nav > Con)
- Detours (Nav > Con)
- New Goal Events (Nav > Con)
- Decision Points (Nav > Con)

a overlap of Decision Points + Detours – medial prefrontal cortex
b overlap of Nav > Con Tasks + New Goal Events – superior frontal gyrus
c overlap of Nav > Con Task + New Goal Events – surpamarginal gyrus / intraparietal sulcus
d overlap of Nav > Con Task + New Goal Events + Decision Points – Retrosplenial cortex
e overlap of New Goal Events + Decision Points – Posterior parietal cortex (angular gyrus)

Figure S2. Navigation versus control. Comparison of the navigation condition with the control condition for the task blocks and the four events. The activation maps are displayed on the mean structural image at a threshold of p < 0.005 uncorrected, 5 voxels minimum cluster size. Values in the top right corner of each image refer to the MNI y-value for that slice. Slices were chosen to optimally display the pattern of response.

A) Peak Responses

Travel Period Events

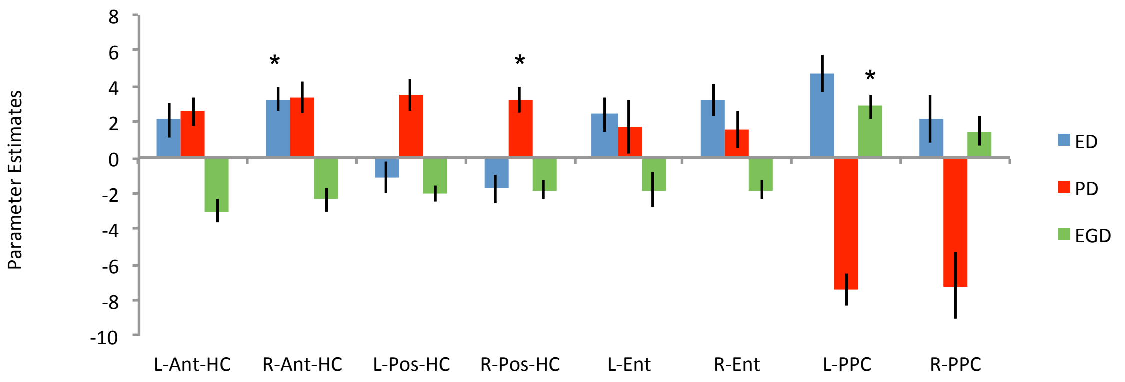

Decision Points

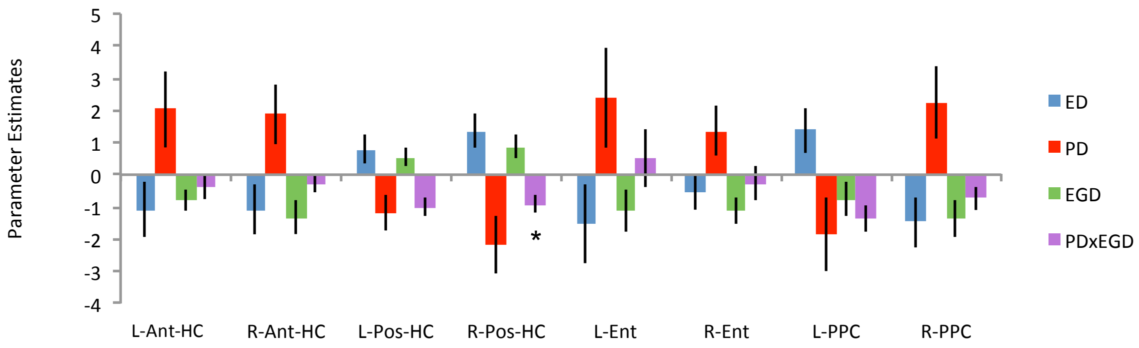

New Goal Events

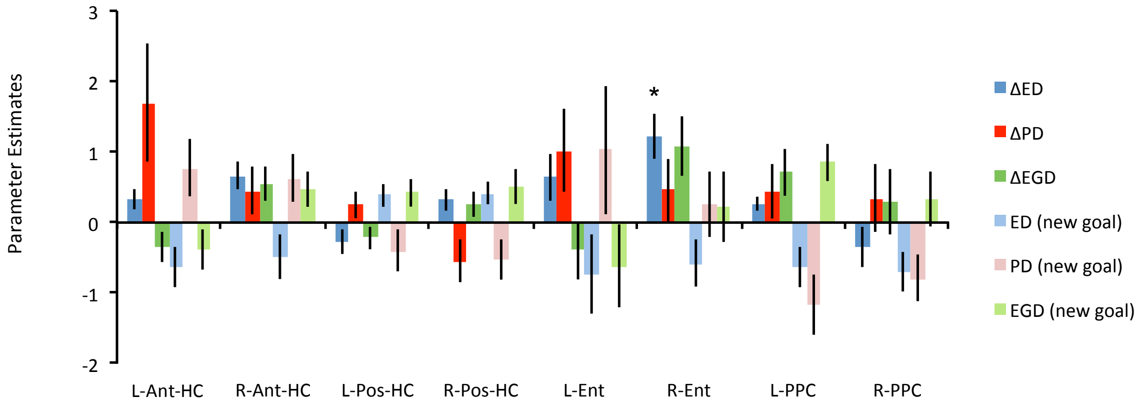

Detours

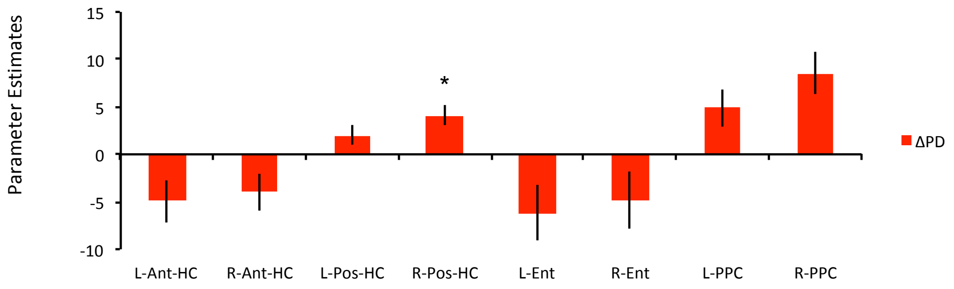

B) Mean Responses

Travel Period Events

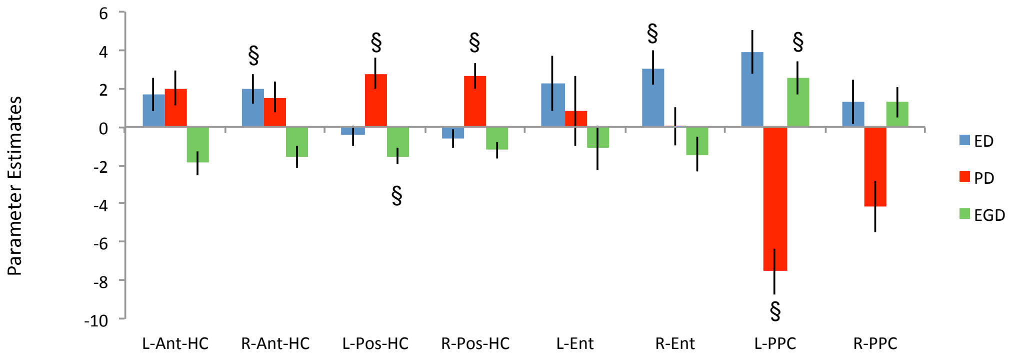

Decision Points

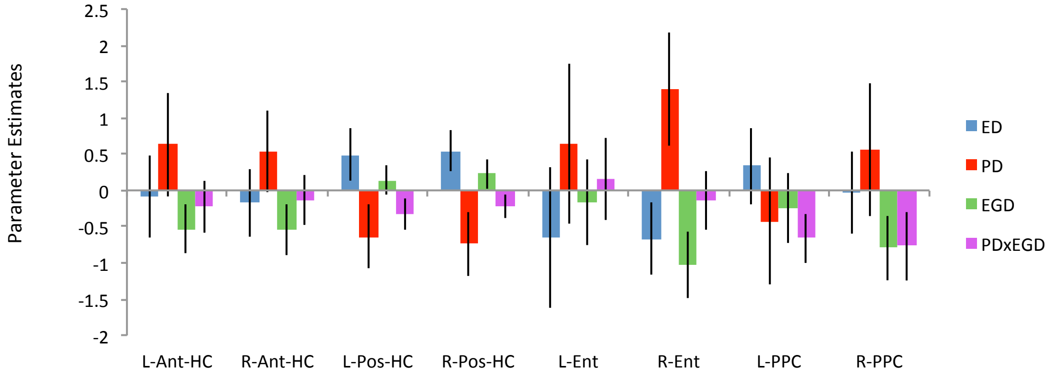

New Goal Events

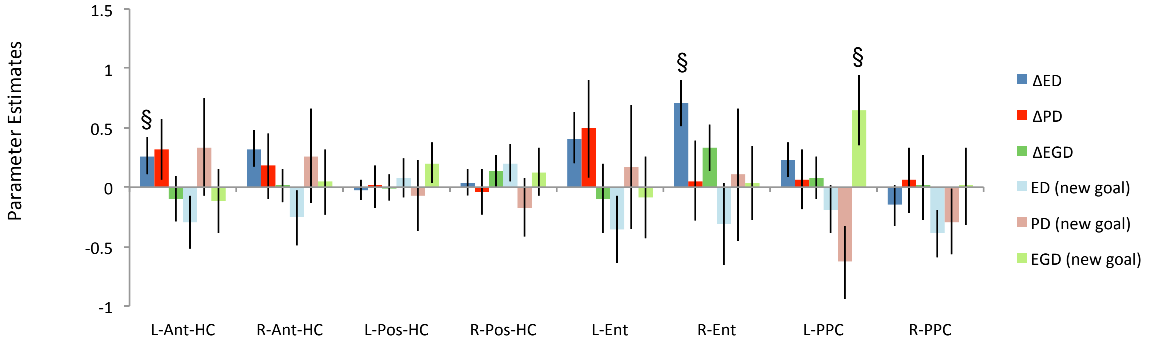

Detours

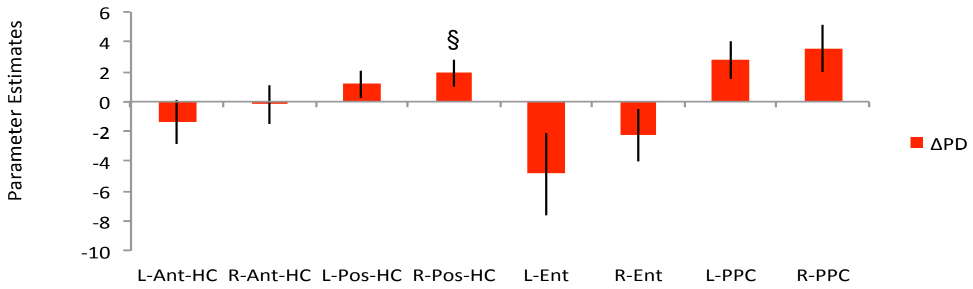

Figure S3. Parameter estimates in each region of interest (ROI) for each spatial parameter for each event type. (**A**) The peak response in each ROI. (**B**) The mean response in each ROI.

ED = Euclidean distance, PD = path distance, EGD = egocentric goal direction. L = Left, R = Right, Ant = anterior, Pos = posterior, HC = hippocampus, Ent = entorhinal cortex, PPC = posterior parietal cortex. Error bars denote SEM. For peak responses in (A) * = significant at $p < 0.05$ corrected for predicted ROIs (right hemisphere for the MTL), see Table S2 for Z-scores derived from SPM. For mean responses in (B) § = significant at $p < 0.05$ for predicted ROIs or $p < 0.05$ bonferroni corrected for other regions, see Table S6 for t-scores and p-values derived from SPSS. See Figures S4 and S5 below for visualisation of the SPM analysis on mean structural images. Note, only regions that showed a significant response and distinct cluster with a minimum of 5 voxels in the ROI were reported in Table S2. There were cases where this criterion was not met, yet a significant mean response in the ROI-based analysis, or a high peak parameter estimate in our ROI, was observed.

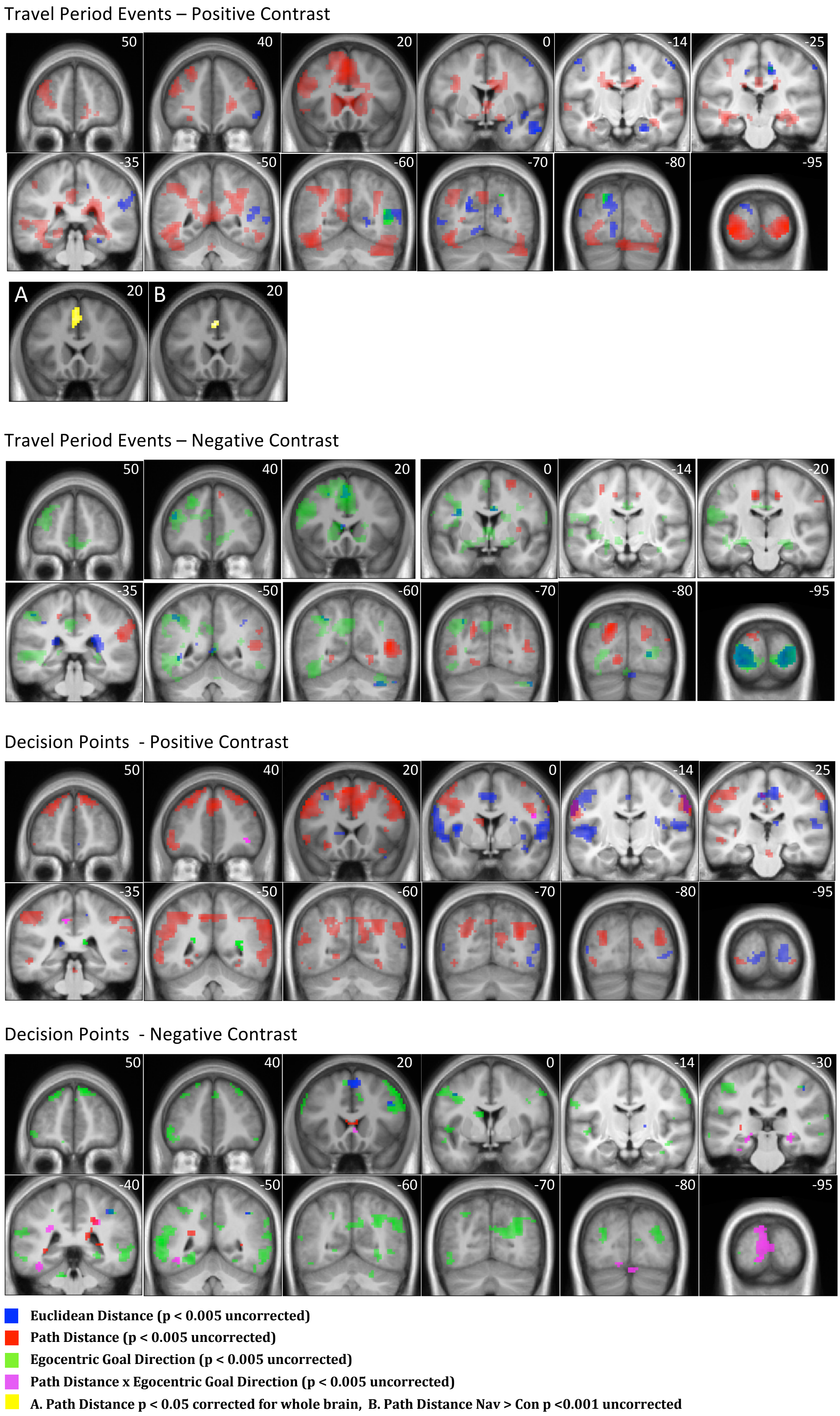
Travel Period Events – Positive Contrast
50 40 20 0 -14 -25
-35 -50 -60 -70 -80 -95
A 20 B 20
Travel Period Events – Negative Contrast
50 40 20 0 -14 -20
-35 -50 -60 -70 -80 -95
Decision Points - Positive Contrast
50 40 20 0 -14 -25
-35 -50 -60 -70 -80 -95
Decision Points - Negative Contrast
50 40 20 0 -14 -30
-40 -50 -60 -70 -80 -95
Euclidean Distance (p < 0.005 uncorrected)
Path Distance (p < 0.005 uncorrected)
Egocentric Goal Direction (p < 0.005 uncorrected)
Path Distance x Egocentric Goal Direction (p < 0.005 uncorrected)
A. Path Distance p < 0.05 corrected for whole brain, B. Path Distance Nav > Con p <0.001 uncorrected

Figure S4 Activity correlated with the spatial parameters at Travel Period Events and Decision Points. The activation maps are displayed on the mean structural, 5 voxels minimum cluster size. Values in the top right corner of each image refer to the MNI y-value for that slice. Slices were chosen to optimally display the pattern of response. Path distance x egocentric distance was only examined at Decision Points. The anterior cingulate activity displayed in images A and B in the section 'Travel Period Events – Positive Contrast' is the only brain region that was both significant at a threshold of $p < 0.05$ (corrected for whole brain volume) for the navigation routes (image A, see Table S2) and also significant at a threshold of $p < 0.001$ uncorrected for Nav > Con for the same parameter and time period (image B: x, y, z, = -3, 20, 37; Z-score = 3.72).

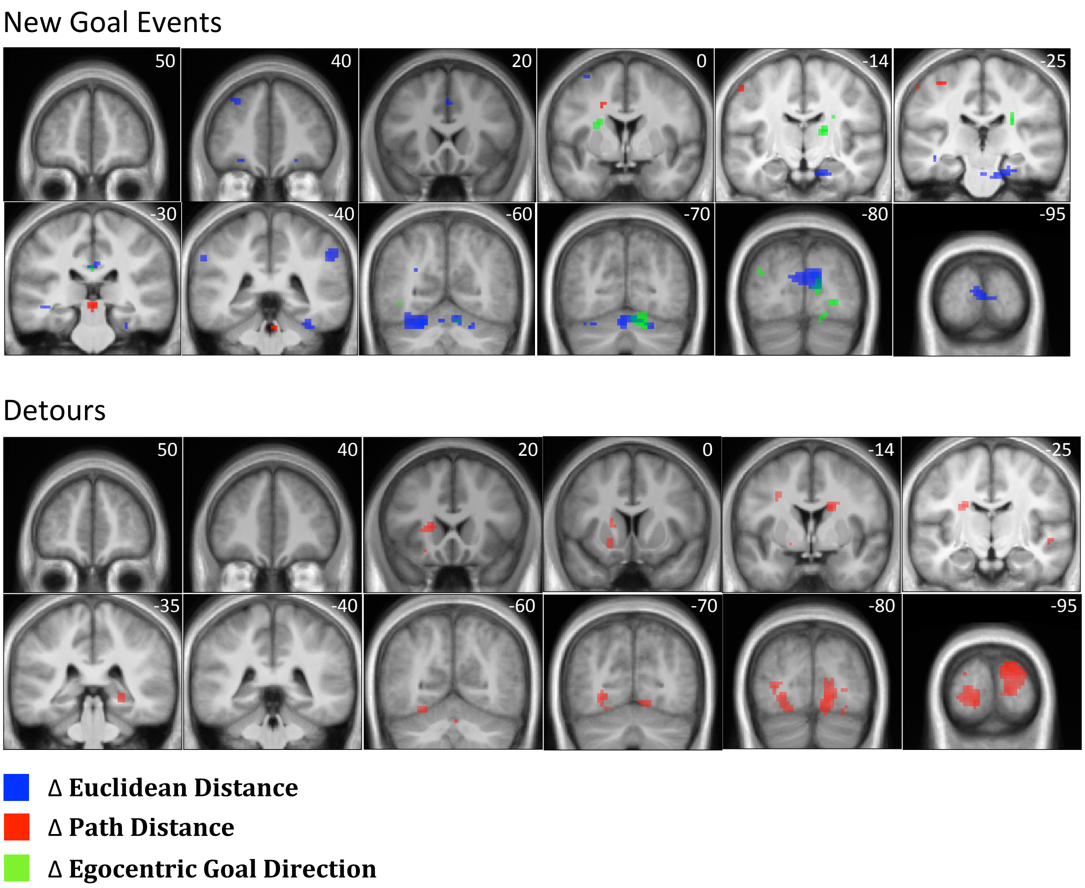

Figure S5 Activity correlated with the change in spatial parameters during New Goal Events and Detours. The activation maps are displayed on the mean structural at a threshold of $p < 0.005$ uncorrected, 5 voxels minimum cluster size. Values in the top right corner of each image refer to the MNI y-value for that slice. Slices were chosen to optimally display the pattern of response. Only path distance changed at Detours.

Supplemental Tables

Table S1. Behavioural Results.

Mean (standard deviation) performance scores (% correct) for the pre- and post-training environmental knowledge assessment

	Pre-training	Post-training
**Streets**	1.44 (3.36)	74.68 (22.55)
**Goals**	1.81 (3.61)	96.92 (6.96)

2x2 repeated measures ANOVA (training phase (pre/post), information type (streets/goals)) revealed a significant main effect of training phase ($F_{(1,23)} = 1092.36$, $p < 0.001$) a significant main effect of information type ($F_{(1,23)} = 23.72$, $p < 0.001$) and a significant interaction ($F_{(1,23)} = 21.00$, $p < 0.001$).

Mean (standard deviation) performance scores (% correct) for New Goal Events and Decision Points in the navigation and control tasks

	Navigation	Control
**New Goal Events**	84.82 (10.96)	95.90 (5.77)
**Decision Points**	79.91 (13.28)	97.63 (5.74)

Mean (standard deviation) reaction times (msec) for New Goal Events and Decision Points in the navigation and control tasks

	Navigation	Control
**New Goal Events**	2036.02 (667.90)	1436.74 (331.71)
**Decision Points**	1902.60 (699.95)	1265.26 (370.63)

Table S1. Behavioural Results (Continued)

Correlation coefficients for behavioural measures and spatial parameters at Decision Points

	Path distance (meters)	Euclidean distance (meters)	Egocentric goal direction (0°- 180°)	Path distance x Egocentric goal direction	Euclidean distance x Egocentric goal direction
**Accuracy**	-0.272*	0.119	-0.342*	-0.367**	-0.117
**Reaction time**	0.363**	-0.099	0.285*	0.409**	0.115

Correlation coefficients for behavioural measures and spatial parameters at New Goal Events

	Change in: Path distance (meters)	***Change in:*** Euclidean distance (meters)	***Change in:*** Egocentric goal direction (0°- 180°)
**Accuracy**	-0.121	-0.049	-0.141
**Reaction time**	-0.005	-0.06	0.143

* sig at p < 0.05, ** sig at p < 0.01. See Results for details of the analysis of the task performance measures.

Table S3. Contrasts between different spatial parameters at the same event type

Brain region	Event type	Parameter comparison	Z-score
Ant. hippocampus	Travel Period Events	ED > PD	1.86
Post. hippocampus	Travel Period Events	ED < PD	3.34*
Post. hippocampus	Decision Points	PDxEGD < ED	2.69 [§]
Entorhinal Cortex	New Goal Events	ΔED > ΔPD	3.31[§]

* significant at $p < 0.05$ corrected for region of interest, 5 voxel minimum cluster size. [§] significant at $p < 0.005$ uncorrected for region of interest, 5 voxel minimum cluster size. Coordinates were very similar to those in the tables above and so are not re-listed here. ED = Euclidean distance, PD = path distance, EGD = egocentric goal direction, Δ = change in the variable, Ant = Anterior, Post = Posterior. In this table ‘<’ and ‘>’ refer to one parameter being significantly more positively, or negatively, correlated relative to the other parameter, not that the absolute correlation of one parameter is greater than the other.

Table S4. Contrasts between the same spatial parameter at different event types

Region	-/+ correlation, Parameter	Event comparison	Z-score
Anterior Hippocampus	+, ED	Travel Period Events > New Goal Events	4.15*
Anterior Hippocampus	+, ED	Travel Period Events > Decision Points	4.04*
Posterior Hippocampus	+, PD	Travel Period Events > New Goal Events	3.00*
Posterior Hippocampus	+, PD	Travel Period Events > Decision Points	3.78*
Posterior Hippocampus	-, PDxEGD	Decision Points > Travel Period Events	3.16*
Posterior Hippocampus	-, PDxEGD	Decision Points > New Goal Events	3.22*
Posterior Hippocampus	+, ΔPD	Detours > New Goal Events	3.26*
Posterior Parietal Cortex	+, EGD	Travel Period Events > New Goal Events	0.33
Posterior Parietal Cortex	+, EGD	Travel Period Events > Decision Points	-0.93

* significant at $p < 0.05$ corrected for region of interest, 5 voxel minimum cluster size. Coordinates were very similar to those in the tables above and so are not re-listed here. ED = Euclidean distance, PD = path distance, EGD = egocentric goal direction, Δ = change in the variable.

Table S5. Analysis of ROI data from the 7 sections through the right hippocampus

MNI-y	Travel<br>ED<br>t (p –value)	Travel<br>PD<br>t (p –value)	Travel<br>ED-PD<br>t (p –value)	Detours<br>ΔPD<br>t (p –value)
-39	-1.05 (0.30)	2.86 (0.010*	-2.22 (0.04*	1.54 (0.14)
-34	-1.30 (0.21)	4.48 (< 0.001)**	-3.75 (0.001)*	2.12 (0.04)*
-29	-0.40 (0.70)	4.31 (< 0.001)**	-2.84 (0.01)*	1.75 (0.09)
-24	0.01 (0.99)	3.71 (0.001)*	-2.39 (0.025)*	0.52 (0.61)
-19	1.25 (0.22)	3.02 (0.01)*	-1.38 (0.18)	-0.09 (0.92)
-14	2.27 (0.01)*	1.79 (0.09)	0.25 (0.81)	-0.15 (0.87)
-9	2.81 (0.01)*	2.19 (0.04)*	0.35 (0.73)	-0.07 (0.95)

ED = Euclidean distance, PD = Path distance, red font indicates a significant response. * significant at $p < 0.05$, MNI-y = MNI y-coordinate values for the middle of the ROI slice through the hippocampus, ** significant at $p < 0.001$ corrected, ED, PD and ΔPD were all independent samples t-tests, n =23, ED-PD comparisons were paired samples t-test, n=23

Table S6. T-scores (uncorrected p-values) from an analysis of ROI mean responses for navigation routes

	L-Ant-HC	R-Ant-HC	L-Pos-HC	R-Pos-HC	L-Ent	R-Ent	L-PPC	R-PPC
**Travel Period Events**								
ED	1.91 (0.068)	2.55 (0.018)§	-0.87 (0.395)	-1.18 (0.250)	1.57 (0.129)	3.51 (0.002)§	3.46 (0.002)	1.14 (0.265)
PD	2.27 (0.033)	1.96 (0.062)	3.54 (0.002)§	4.07 (0.000)§	0.45 (0.655)	0.02 (0.982)	-6.32 (0.000)§	-3.06 (0.006)
EGD	-2.95 (0.007)	-2.56 (0.017)	-3.68 (0.001)§	-2.87 (0.009)	-0.91 (0.371)	-1.62 (0.118)	2.95 (0.007)§	1.63 (0.116)
**Decision Points**								
ED	-0.14 (0.889)	-0.38 (0.711)	1.38 (0.182)	1.87 (0.182)	-0.67 (0.508)	-1.32 (0.200)	0.64 (0.531)	-0.07 (0.946)
PD	0.88 (0.386)	0.96 (0.348)	-1.47 (0.154)	-1.68 (0.154)	0.58 (0.565)	1.80 (0.086)	-0.49 (0.629)	0.61 (0.547)
EGD	-1.58 (0.128)	-1.54 (0.138)	0.69 (0.497)	1.08 (0.497)	-0.29 (0.776)	-2.25 (0.034)	-0.52 (0.607)	-1.77 (0.089)
PDxEGD	-0.65 (0.525)	-0.36 (0.724)	-1.54 (0.138)	-1.45 (0.138)	0.27 (0.790)	-0.36 (0.724)	-1.93 (0.065)	-1.61 (0.121)

Table S6. T-scores (uncorrected p-values) from an analysis of ROI mean responses for navigation routes (Continued)

	L-Ant-HC	R-Ant-HC	L-Pos-HC	R-Pos-HC	L-Ent	R-Ent	L-PPC	R-PPC
**New Goal Events**								
ΔED	1.67 (0.109)	2.14 (0.043)§	-0.23 (0.820)	0.36 (0.719)	1.95 (0.064)	3.58 (0.002)§	1.57 (0.131)	-0.88 (0.389)
ΔPD	1.25 (0.224)	0.64 (0.530)	0.02 (0.985)	-0.22 (0.830)	1.20 (0.243)	0.17 (0.871)	0.27 (0.789)	0.22 (0.830)
ΔEGD	-0.48 (0.634)	0.10 (0.920)	-0.01 (0.989)	0.96 (0.349)	-0.34 (0.739)	1.68 (0.107)	0.46 (0.648)	0.01 (0.995)
ED	-1.30 (0.206)	-1.08 (0.293)	0.47 (0.643)	1.31 (0.202)	-1.27 (0.217)	-0.90 (0.375)	-0.91 (0.372)	-1.92 (0.068)
PD	0.82 (0.422)	0.67 (0.513)	-0.24 (0.816)	-0.69 (0.497)	0.33 (0.745)	0.19 (0.850)	-2.03 (0.054)	-1.06 (0.301)
EGD	-0.42 (0.679)	0.17 (0.868)	1.20 (0.244)	0.62 (0.540)	-0.24 (0.812)	0.11 (0.913)	2.20 (0.038)§	0.02 (0.982)
**Detours**								
PD	-0.92 (0.369)	-0.15 (0.880)	1.25 (0.225)	2.12 (0.045)§	-1.75 (0.094)	-1.29 (0.209)	2.18 (0.040)	2.24 (0.035)

ED = Euclidean distance, PD = Path Distance, EGD = Egocentric Goal Direction, L = Left, R = right, Ant = anterior, Pos = Posterior. Red font indicates significant values. § = $p < 0.05$ for *a priori* regions of interest (see Procedures below) or $p < 0.05$ bonferroni corrected for multiple comparisons with each event type (16 comparisons for Travel Period Events, 21 for Decision Points, 35 for New Goal Events, and 5 for Detours).

Supplemental Experimental Procedures

Subjects

Twenty-four right-handed, healthy volunteers (13 males, mean age = 26.25 years, SD = 3.52 years, range = 20 – 35 years) with normal or corrected to normal vision participated in this experiment. All subjects were free from colour blindness, neurological and psychiatric disease and gave informed written consent in accordance with the local research ethics committee. Only subjects who reported minimal, or no experience, with the environment were invited to take part in the study. Subjects were also screened with the Santa Barbara Sense of Direction Scale [S1]. To avoid testing poor navigators, only those scoring over 3.6 (1 SD below the mean score provided by [S1]) were selected. Subjects tested in our study had a mean score of 4.89 (SD = 0.68).

Test Environment

Soho, London UK, was selected due to its high density of streets (increasing Decision Point sampling) and large number of pubs, clubs, restaurants, cafes and shops, which served as useful landmarks and goals. The region was bounded by Oxford Street in the north, Brewer Street in the south, Lexington Street to the West, and Dean Street to the East. We used a real-world environment rather than a virtual one, to allow subjects the full range of natural sensory cues to encode the space, which has been found to improve spatial memory [S2].

Assessment of Prior Knowledge of the Environment

We assessed subjects' prior knowledge of the environment precisely one week prior to the day of scanning. Subjects were shown a map of the region used with all street names removed, except Oxford Street (Figure S1). They were asked to label as many of the streets as they could. Next, they were shown the same map with street names shown, a set of red dots marked and a list of landmarks (Figure S1). Subjects are asked to indicate if they were familiar with any of the landmarks, and if so to indicate which of the red dots identified its location.

Training

Subjects were required to learn the layout of 26 streets and the location of 23 goals within our test environment in Soho (Figures 1, 2 and S1). The training strategy employed was based on the method London taxi drivers use to learn 'The Knowledge' of London. To ensure that subjects acquired accurate knowledge about the topography of Soho we provided both survey- and ground-level information throughout training. Following completion of the prior knowledge assessment, subjects were given a pack of training materials, this contained: coloured photographs of the 23 goals along with their locations (Figure S1), coloured photographs of 10 start positions along with their locations, a list of 5 routes across the test environment to learn, blank maps for self-testing, and a set of instructions. Subjects were instructed to spend at least 30 minutes looking at each set of photographs with the aim of memorising the location of each goal and start position.

All subjects later confirmed this was the case. Subjects were instructed that they would be expected to remember the name and location of each goal/start solely from presentation of the photograph. To facilitate this process self-testing was encouraged and blank maps, along with examples of the kind of self-testing that might be useful, were enclosed. Finally, to encourage subjects to think about the street layout and devise optimal routes between locations, subjects were required to devise and memorise the optimal (shortest) route between 5 sets of locations (only small sub-sections of these routes overlapped with the routes during scanning). A monetary incentive (£2 extra payment if they scored >70% correct across all tasks) was used to encourage subjects to utilise their training packs as much as possible. Importantly, subjects were specifically told not to use any other maps of Soho to aid their training and instead to rely solely on the materials in this pack.

On the day prior to scanning, subjects were taken on a two-hour tour of the test region in Soho, during which their spatial knowledge was rigorously tested and feedback was given. All subjects were taken on the same training route. This was carefully designed so that each start location was visited once and each goal location was passed at least twice and from different directions. When each of these locations was reached the experimenter showed subjects the coloured photograph of the start or goal as well as their current position on a map. Throughout the tour the experimenter highlighted all useful topographical information and encouraged the subjects to attend to landmarks that would be salient and/or useful for orientation during the subsequent navigation task (i.e., they were highly salient in the movie footage). Subjects were periodically probed about their knowledge of

upcoming streets and goals (e.g. ‘What is the name of the next street coming up on the left?’, ‘There are two goals ahead of us on this road; do you know what they are?’). They were also asked at 6 different locations to indicate, via pointing, the direction along the Euclidean distance to distant goals and describe the optimal route to reach them. None of these goals were later tested from these street segments in the fMRI task. Feedback was provided to the subject for each question asked, using the map where necessary, to ensure subjects benefitted as much as possible from the tour experience. Immediately after the tour, subjects were taken to a café and their post-training knowledge of the test environment was assessed, using the same procedure that had been used pre-training. Immediate feedback was provided to guide subjects towards any aspects they should ‘revise’ on the final evening before scanning. At no point during the training were subjects asked to estimate the path or Euclidean distance.

Stimuli and task

Details of the stimuli are presented in Figure 2. The 10 routes were novel combinations of the streets experienced during the guided tour. Immediately prior to the scan session itself, a training session was conducted to ensure that participants were pre-exposed to both route types and understood the task requirements. A total of 3 training routes were viewed (2 navigation and 1 control). During this period, subjects experienced multiple changes to the goal at several New Goal Events, and thus were aware that their goal would change from time to time during scanning. They were reminded that they must pay attention to the current

goal in order to perform correctly. During scanning, routes were separated by a 17 second interval. During the first 12 seconds of this a centrally positioned white fixation cross was presented on a grey screen and during the latter 5 seconds, just before each route commenced, the fixation cross was replaced with either the cue 'NAVIGATE' or 'CONTROL'; to indicate which type of route would follow. Throughout each route the instruction 'NAV' or 'CON' was displayed at the top of the screen, depending on the route type. The mean duration of the routes was 266.60 sec (SD = 43.63, range = 198 – 325). Routes were presented at walking speed (mean = 1.6 m/s SD = 0.41). The routes were designed to minimize the correlation between Euclidean and path distance to the goal and to maximise the number of turns experienced. Each route was produced in two task formats: Navigation and Control. Route and task were counterbalanced across subjects.

Correlations between the spatial parameters across event types

Correlation Coefficients comparing parameters across all routes

	Euclidean – Path	Euclidean – EGD	Path – EGD
Travel Period Events	0.49*	-0.04	0.06
Decision Points	0.58*	-0.15	0.46*
New Goal Events (change in values)	0.05	-0.24	0.52*

Mean Correlation Coefficients (with range) of the each of the routes

	Euclidean – Path	Euclidean – EGD	Path – EGD
Travel Period Events	0.21 (-0.12 - 0.46)	-0.13 (-0.43 - 0.31)	-0.18 (-0.34 - 0.10)
Decision Points	0.38 (-0.66 - 0.95)	-0.29 (-0.89 - 0.41)	-0.19 (-0.73 - 0.48)
New Goal Events (change in values)	0.24 (-0.56 - 0.79)	-0.08 (-0.64 - 0.63)	0.20 (-0.60 - 0.75)

EGD = Egocentric Goal Direction, * significant at $p < 0.05$

For all routes, the duration that start images remained on screen prior to the display of the first New Goal Event in each route was temporally jittered to last between 5 and 13 seconds. Timing remained constant for New Goal Events and Decision Points. New Goal Events lasted 9 seconds in total, during which the movie was paused. In the initial 4 seconds a colour photograph of the new goal was overlaid, along with text describing its location. For the remaining 5 seconds the photograph of the goal remained but the location description was replaced with the question 'GOAL L/R?'. During this time the subject was expected to make their button press response. Decision Points lasted 5 seconds, during which the movie was paused and the subject was presented with the options to turn at the junction ahead (e.g. 'TURN L/S/R?'), and again, subjects were expected to respond during this time. The amount of time between Decision Points and the onset of the following turn was temporally jittered to last between 3 and 9 seconds to allow separate measures of the BOLD signal at these two events. After each turn, at the beginning of each new street section, text appeared on screen for 3 seconds describing the subject's

current location and general heading direction (e.g., ‘Broadwick st, facing east’). At the end of each route, the duration of the final shot of each movie instructing the subject that the final destination had been reached was also temporally jittered so that it remained on screen for between 3 and 9 seconds. In control routes, subjects were instructed which button to press at Decision Points, and had to decide whether it was possible to purchase a drink at the goal location during New Goal Events. Subjects were informed that they could use the street name and direction information presented on entering new streets to orient in control routes, but they must not think about navigating to the goals presented in the New Goal Events.

Immediately after scanning (outside the scanner), subjects completed a debrief interview where all navigation routes viewed during scanning were re-presented in the same order on a laptop (screen size: 12 inch). We do not report data from this debriefing here. Response time and accuracy scores were calculated by comparing the subjects button presses during fMRI scanning with the correct answers based on measurements of the ideal paths and directions to the goal. Statistical analysis of these and all other behavioural data was conducted with SPSS (© IBM Corp).

The ten routes within the test region of Soho were filmed using a HD Sony Z1 and a camera stabilizer (B Hague). Final Cut Pro was used to edit and overlay text onto the original footage to form the first-person-view movie stimuli used in the experiment. Minimizing the correlation was achieved by selecting specific combinations of starting locations, New Goal Events, and Detours. Because of the geometric relationship between these two parameters some degree of correlation

was inevitable, particularly given that at the end of each route the subject reached the goal location and that the goal was not changed too frequently. These constraints were determined to be important from pilot studies. Session 1 started with a navigation route, while session 2 started with a control route. MATLAB 7.5 (© Mathworks) and the Cogent2000 v1.28 Toolbox (http://www.vislab.ucl.ac.uk/cogent_2000.php) were used to control stimulus presentation, interact with the scanner and record response data. All button press responses were made using a button box positioned in the subject's right hand.

Calculation of spatial parameters

Distance and direction data were derived as follows. For each route, the latitude and longitude of start and end points, as well as all those of all the street junctions in between were determined using the program Google Earth (© Google 2010) and converted into Northings and Eastings on a transverse Mercator projection using software from DMAP (©Alan Morton). Each of these coordinates was given a time stamp, indicating the time since the start of that route. MATLAB 7.5 (© Mathworks) was used to provide a linear interpolation over these coordinates to create an estimate of the viewers' spatial position for every second of every route movie. Coordinates of the goal locations were used to create a record of Euclidean distance and egocentric direction to the goal. Euclidean distance measurements were re-scaled across all 10 routes to be between 0 and 1, where a value of 0 corresponded to being at the goal and a value of 1 to being at the maximum Euclidean distance from the goal.

The path distance was the length of the optimal (shortest) route to the current goal. This was measured by summing the length, in meters, of all the component street sections that made up the optimal route. Path distance scores were re-scaled across all 10 routes to be between 0 and 1, where a value of 0 corresponded to being at the goal and a value of 1 to being at the maximum path distance from the goal.

To calculate the egocentric direction to the goal, we first determined the current heading direction (along the route) and the heading direction pointing directly to the goal at each location on each route. The current heading direction was determined by finding the phase angle between current location and the location of the viewer 1 second later on the route. Because there was no future location for each final location, we assumed the viewer was heading in the same direction at the final location as the location occupied 1 second previously. The heading direction pointing directly to the goal was determined by finding the phase angle between the current location and the goal location. The egocentric direction to the goal was the (smallest) angular difference between the current heading direction and the heading direction pointing to the goal. In this study, our main focus was on measuring the overall variation in the egocentric direction toward the goal, thus we collapsed across left and right directions. This meant that values greater than 180° were then subtracted from 360° to bring all values into a range between 0° and 180°. Egocentric direction values were re-scaled across all 10 routes to be between 0 and 1, where a value of 0 corresponded to the goal being directly in front of the subject (0°) and 1 corresponded to the goal being directly behind the subject (180°).

fMRI acquisition and analysis

Participants were scanned at the Birkbeck-UCL Centre for Neuroimaging (BUCNI) using a 1.5 Tesla Siemens Avanto MRI scanner (Siemens Medical Systems, Erlangen, Germany), with a 32-channel head coil. The experimental task, performed over two sessions, lasted around forty-five minutes and twenty-six seconds. A total of nine hundred and forty-one (± 2) functional scans were acquired using a gradient-echo echoplanar imaging (GE-EPI) sequence (TR = 2,897 ms, TE = 50 ms, flip angle = 90°, FoV = $192mm^2$). In each volume thirty-four oblique axial slices, approximately perpendicular to the hippocampus and 3 mm thick were acquired. Following this a high-resolution T1 structural scan was acquired (MPRAGE, 176 slices, 1 x 1 x 1 mm resolution). Foam padding was used to minimise head motions and ear-plugs were used to dampen the noise of the scanner. Stimuli were projected centrally onto a screen at the front of the magnet which participants viewed using a mirror mounted on the head coil (21 x 13 degrees of visual angle of the whole screen). The first 6 functional volumes of each session (dummy scans) were discarded to permit T1 equilibrium. Statistical parametric mapping (SPM8; http://www.fil.ion.ucl.ac.uk/spm/software/spm8/) was used for spatial preprocessing and subsequent analyses. Images were spatially realigned to the first volume of the first session to correct for motion artefacts, coregistered with the structural scan, normalised to a standard EPI template in Montreal Neurological Institute (MNI) space, and spatially smoothed with an isotropic 8 mm FWHM Gaussian kernel filter.

After preprocessing, the smoothed, normalised functional imaging data were entered into a voxel-wise subject-specific general linear model (GLM) (i.e., the first level design matrix). The effects of interest were task epochs (navigation or control) and event-related effects corresponding to: New Goal Events (9 sec duration), Decision Points (5 sec duration), turns along the optimal route (6 sec duration), Detours (6 sec duration) and Travel Period Events (which were time points (zero duration) during the travel periods equidistant between the other events). The number of events varied across subjects because route and task were counter-balanced. Thus, for navigation routes numbers of each type of event were: 21 or 22 New Goal Events, 26 or 27 Decision Points, 12 or 14 Detours, 79 or 80 Travel Period Events. There was the same variation in control route event numbers, e.g. 21 or 22 New Goal Events in control routes. Regressors for each of events/epochs were entered separately for navigation and control routes. For this GLM, the regressors of interest and six subject-specific movement parameters (included as regressors of no interest) derived from the realignment phase of preprocessing, were included. The periods of fixation between blocks was not modelled and treated as the implicit baseline. Each of the regressors of interest was then convolved with the canonical haemodynamic response function (HRF) and a high pass filter with a cut-off of 128 s was used to remove low-frequency drifts. Temporal autocorrelation was modelled using an AR(1) process. At the first level, linear weighted contrasts were used to identify effects of interest, providing contrast images for group effects analysed at the second (random-effects) level. The basic GLM was used to contrast navigation and control tasks and key events as well as Detours with congruent turns (intended route progress).

Following this, in a series of GLM analyses we probed the fMRI data with the spatial parameters (Euclidean distance, path distance, and egocentric goal direction). Parametric regressors were *not* serially orthogonalized, thus allowing each regressor to account independently for the response at each voxel. Separate GLMs were generated to target specific hypotheses about the data at the four event types in our study (Travel Period Events, New Goal Events, Decision Points, and Detours). Each GLM explored the first order parametric modulation of the events of that type, for both navigation and control routes. We did not exclude any New Goal Events or Decision Points from our analysis on the basis of subjects' performance. The table below provides details of the variables assessed in the different GLMs.

General Linear Models with parametric analysis

**Time period**	**Parameters**
Travel Period Events	ED, PD, EGD
Travel Period Events	EDxEGD, PD
Travel Period Events	ED, PDxEGD
Decision Points	ED, PD, EGD, RT
Decision Points	ED, PDxEGD, RT
New Goal Events	ΔED, ΔPD, ΔEGD
New Goal Events	ED, PD, EGD (based on new goal location)
Detours	ΔPD
**Additional models for follow up analyses**	
Travel Period Events (25% events removed)*	ED, PD, EGD
Travel Period Events	ED, PD, EGD, TE

General Linear Models with parametric analysis continued.

**Additional models to allow comparison between events**	
Travel Period Events, Decision Points & New Goal Events	ED, PD, EGD
Travel Period Events, Decision points & New Goal Events	ED, PDxEGD
New Goal Events & Detours	ΔDP

All models contained all the key events (Travel Period Events, New Goal Events, Decision Points, Detours), plus navigation task blocks, control task blocks, non-detour turns. The implicit baseline contained the 17 sec period of fixation between task blocks.

ED = Euclidean distance, PD = path distance, EGD = egocentric goal direction, TE = time elapsed since route started, RT = reaction time, Δ = change in the variable. * 25% events were removed which contained the highest correlation between PD and ED.

In our models exploring Decision Points*,* we included reaction times (RTs) as a regressor because we observed significant correlations between both path distance and egocentric goal direction with reaction time at Decision Points (Table S1). In light of this behavioural result we examined Decision Points with a model in which the path distance and direction regressors were replaced with a regressor composed of the multiplication of both regressors. We also examined Travel Period Events by applying a similar approach to examine the interaction of the direction with both types of distance. In a further set of GLMs we investigated the parametric effect of the magnitude of the change in path distance at Detours and of the change in all

three spatial parameters at New Goal Events, during both navigation and control routes.

The next set of GLM analyses comprised a variety of control analyses. These aimed to explore whether MTL activity was also modulated by other potential explanatory variables. To summarise, the analyses examined the effect of: 1) time elapsed on the routes, 2) the number of choices at Decision Points, 3) goals moving nearer or further away at New Goal Events, and 4) the impact of removing events from the analysis which contained high correlations between path and Euclidean distance during travel periods. In order to assess the number of choices at Decision Points, the regressor for Decision Points was divided into two regressors, one for events with two choices (T-junctions) the other for events with three choices (crossroads). In the second analysis, two types of New Goal Events (goal moves closer-to vs. goal moves farther-from subjects) were compared in order to determine whether our MTL responses to the change in distance to the goal was driven solely by instances when the goal moves closer-to or farther-from subjects. Two models were constructed for this analysis, one in which the goal moved closer/farther in terms of Euclidean distance, the second for instances where the goal moved closer/farther in terms of path distance. Because hippocampal activity was significantly correlated with both path and Euclidean distance during travel periods, we also examined whether MTL activity was significantly correlated with these parameters by conducting our model of travel periods with the modification that 25% of the events containing the most correlated path and Euclidean distance were removed.

In order to plot parameter estimates for different levels of Euclidean distance to the goal at each event type (e.g. Decision Points) new GLMs were created in which events were assigned to one of four regressors according to the magnitude of their corresponding value. Each regressor reflected 1/4th of the total range of values. Thus, for example when plotting path distance, the first of these regressors reflected events when the subject was nearest to the goal, while the fourth regressor reflected events when the subject was far from the goal. These values were extracted from the sampled events of each participant. Therefore they were not evenly spread over the normalised scale of 0-1. The number of Detours was lower than the rest of the events. Therefore, in order to keep the variance comparable across different event types, path distance values at Detours were split into only three regressors (Figure 5). Note, these GLMs were employed purely for plotting the data and were not used for significance testing.

Given our *a priori* anatomical hypotheses, for distance correlates we specifically report activations in the hippocampus and entorhinal cortex at a threshold of $p < 0.05$ (family-wise-error corrected for brain volume determined by ROIs) and minimum of 5 contiguous voxels. Due to current speculation about the role of the anterior and posterior hippocampus, we used ROIs in the anterior and posterior hippocampus and entorhinal cortex. We focused on the right hemisphere because the right MTL has been more consistently associated with spatial memory in humans (see e.g. [S3–S8]) Both the entorhinal cortex and the hippocampal ROIs were defined using the Duvernoy hippocampal atlas [S9] and Insausti et al. [S10] as guides. Anterior was defined as the most anterior 3rd of the hippocampus, and

posterior as the most posterior 3rd. For follow up contrasts (having initially established a significant response in navigation routes) we explored the MTL data with a more liberal threshold of $p < 0.005$ uncorrected, as we have done in prior work [S11]. To examine the prediction that posterior parietal cortex would encode the egocentric goal direction we used 10 mm spheres located at specific Montreal Neurological Institute (MNI) coordinates [27, -87, 36 and -18, -81, 36] based on [S4]. To plot the response along the longitudinal axis we divided the hippocampus into 7 sections running from the anterior limit to the posterior limit in the right hemisphere: each ROI contained 3 slices of 3mm thickness. This ROI approach was used to display the response along the long axis of the hippocampus. In order to also report left hemisphere MTL parameter estimates we also constructed ROIs for the left anterior hippocampus, left posterior hippocampus and left entorhinal cortex using the same procedure as above. Statistical analyses of mean responses in ROIs were conducted in SPSS, using bonferroni correction to account for multiple comparisons where we had no *a priori* predictions. For completeness, we report all brain regions at a threshold of $p < 0.001$ uncorrected (or $p < 0.005$ for MTL regions) and minimum of 5 contiguous voxels for the planned contrasts in Table S2. We also note which regions survive at a threshold of $p < 0.05$ corrected for whole brain volume. All t-scores and p-values (uncorrected) from our ROI analyses are reported in Tables S5 and S6.

Supplemental References

S1. Hegarty, M., Montello, D. R., Richardson, A. E., Ishikawa, T., and Lovelace, K. (2006). Spatial abilities at different scales: Individual differences in aptitude-test performance and spatial-layout learning. Intelligence *34*, 151–176.

S2. Waller, D., Loomis, J. M., and Haun, D. B. M. (2004). Body-based senses enhance knowledge of directions in large-scale environments. Psychon. Bull. Rev. *11*, 157–163.

S3. Spiers, H. J., Burgess, N., Maguire, E. A., Baxendale, S. A., Hartley, T., Thompson, P. J., and O'Keefe, J. (2001). Unilateral temporal lobectomy patients show lateralized topographical and episodic memory deficits in a virtual town. Brain *124*, 2476–2489.

S4. Spiers, H. J., and Maguire, E. A. (2007). A navigational guidance system in the human brain. Hippocampus *17*, 618–626.

S5. Maguire, E. A., Burgess, N., Donnett, J. G., Frackowiak, R. S., Frith, C. D., and O'Keefe, J. (1998). Knowing where and getting there: a human navigation network. Science *280*, 921–924.

S6. Maguire, E. A., Frackowiak, R. S., and Frith, C. D. (1997). Recalling routes around london: activation of the right hippocampus in taxi drivers. J. Neurosci. Off. J. Soc. Neurosci. *17*, 7103–7110.

S7. Baumann, O., and Mattingley, J. B. (2013). Dissociable representations of environmental size and complexity in the human hippocampus. J. Neurosci. Off. J. Soc. Neurosci. *33*, 10526–10533.

S8. Bohbot, V. D., Kalina, M., Stepankova, K., Spackova, N., Petrides, M., and Nadel, L. (1998). Spatial memory deficits in patients with lesions to the right hippocampus and to the right parahippocampal cortex. Neuropsychologia *36*, 1217–1238.

S9. Duvernoy, H. M. (2005). The Human Hippocampus: Functional Anatomy, Vascularization and Serial Sections with MRI (Springer).

S10. Insausti, R., Juottonen, K., Soininen, H., Insausti, A. M., Partanen, K., Vainio, P., Laakso, M. P., and Pitkänen, A. (1998). MR volumetric analysis of the human entorhinal, perirhinal, and temporopolar cortices. Am. J. Neuroradiol. *19*, 659–671.

S11. Howard, L. R., Kumaran, D., Ólafsdóttir, H. F., and Spiers, H. J. (2011). Double dissociation between hippocampal and parahippocampal responses to object-background context and scene novelty. J. Neurosci. Off. J. Soc. Neurosci. *31*, 5253–5261.
