# Supplementary material for: The Hippocampus and Entorhinal Cortex Encode the Path and Euclidean Distances to Goals during Navigation
Source: Curr Biol. 2014 Jun 16;24(12):1331–40. doi: 10.1016/j.cub.2014.05.001 (PMC4062938; doi:10.1016/j.cub.2014.05.001)
Supplement: Document S2. Article plus Supplemental Information [file mmc3.pdf]

Current Biology 24, 1331–1340, June 16, 2014 ©2014 The Authors http://dx.doi.org/10.1016/j.cub.2014.05.001

Article

The Hippocampus and Entorhinal Cortex Encode the Path and Euclidean Distances to Goals during Navigation

Lorelei R. Howard,[1,2] Amir Homayoun Javadi,[1] Yichao Yu,[3] Ravi D. Mill,[4] Laura C. Morrison,[1] Rebecca Knight,[5] Michelle M. Loftus,[1] Laura Staskute,[1] and Hugo J. Spiers[1,*]
[1]UCL Institute of Behavioural Neuroscience, Research Department of Experimental Psychology, Division of Psychology and Language Sciences, University College London, London WC1H 0AP, UK
[2]Aging & Cognition Research Group, German Center for Neurodegenerative Diseases (DZNE), 39120 Magdeburg, Germany
[3]UCL Centre for Advanced Biomedical Imaging, University College London, London WC1E 6DD, UK
[4]School of Psychology & Neuroscience, University of St. Andrews, Fife KY16 9JP, UK
[5]Department of Psychology, University of Hertfordshire, Hertfordshire AL10 9AB, UK

Summary

Background: Despite decades of research on spatial memory, we know surprisingly little about how the brain guides navigation to goals. While some models argue that vectors are represented for navigational guidance, other models postulate that the future path is computed. Although the hippocampal formation has been implicated in processing spatial goal information, it remains unclear whether this region processes path- or vector-related information.
Results: We report neuroimaging data collected from subjects navigating London's Soho district; these data reveal that both the path distance and the Euclidean distance to the goal are encoded by the medial temporal lobe during navigation. While activity in the posterior hippocampus was sensitive to the distance along the path, activity in the entorhinal cortex was correlated with the Euclidean distance component of a vector to the goal. During travel periods, posterior hippocampal activity increased as the path to the goal became longer, but at decision points, activity in this region increased as the path to the goal became closer and more direct. Importantly, sensitivity to the distance was abolished in these brain areas when travel was guided by external cues.
Conclusions: The results indicate that the hippocampal formation contains representations of both the Euclidean distance and the path distance to goals during navigation. These findings argue that the hippocampal formation houses a flexible guidance system that changes how it represents distance to the goal depending on the fluctuating demands of navigation.

Introduction

The mammalian brain has developed a remarkable capacity to create an internal map of space and keep track of current heading direction. Evidence of a cognitive map comes from the spatially localized firing of hippocampal "place cells" and entorhinal "grid cells," which code for an animal's current position in an environment [1, 2]. "Head direction cells" in companion structures [3] provide a signal for orientation. Despite substantive gains in understanding how these cells support spatial cognition, we know surprisingly little about how the brain uses such information to guide navigation.

While numerous functional MRI (fMRI) studies have explored the neural correlates of navigation [4–16], few have tested predictions from computational models. Such models have mainly used one of two mechanisms for guidance: (1) the straight-line Euclidean distance to the goal is computed as part of a heading vector, allowing shortcuts to be detected [17–21]; and (2) the path to the goal is computed, enabling optimal routes to be selected and dead ends to be avoided [22–27]. These two mechanisms provide divergent predictions about how neural activity will be modulated by the distance to the goal during navigation, but both implicate medial temporal lobe (MTL) structures. Path-processing models can be interpreted as predicting that MTL activity will reflect the distance along the intended path to the goal (path distance) because computational demands will vary with the path distance. By contrast, vector models argue that neurons provide a firing-rate population vector proportional to the Euclidean distance to the goal. Recently, it has been argued that the anterior hippocampus provides a global representation of the environment, whereas the posterior hippocampus contains a fine-grained representation [15, 28]. Thus, it is possible that the anterior and posterior hippocampus contain different representations of the distance to the goal such that the posterior codes the specific regions of space forming the path and the anterior codes more global Euclidean distance information.

To test these predictions, we used fMRI and a novel real-world navigation task in which the Euclidean distance and the path distance to the goal had separable values over time. We found that MTL activity was correlated with both the path distance and the Euclidean distance during navigation and that the relationship between MTL activity and these spatial metrics depended on the task demands at different stages of navigation.

Results

Prior to scanning, subjects learned, via studying maps and an intensive walking tour, the layout of a previously unfamiliar environment: the Soho district in London (Figures 1 and 2; Figure S1, available online). The day after the tour, subjects were scanned while watching ten first-person-view movies of novel routes through the environment. Five of the movies required subjects to make navigational decisions about how to reach goal locations (navigation routes), and the other five required no navigational decision making (control routes). Movies and tasks were counterbalanced across subjects. At the start of each navigation route, subjects were oriented as to where they were, and then shortly after (a period temporally jittered to be between 5 and 13 s), they were shown a goal

*Correspondence: h.spiers@ucl.ac.uk
This is an open access article under the CC BY license (http://creativecommons.org/licenses/by/3.0/).
CrossMark

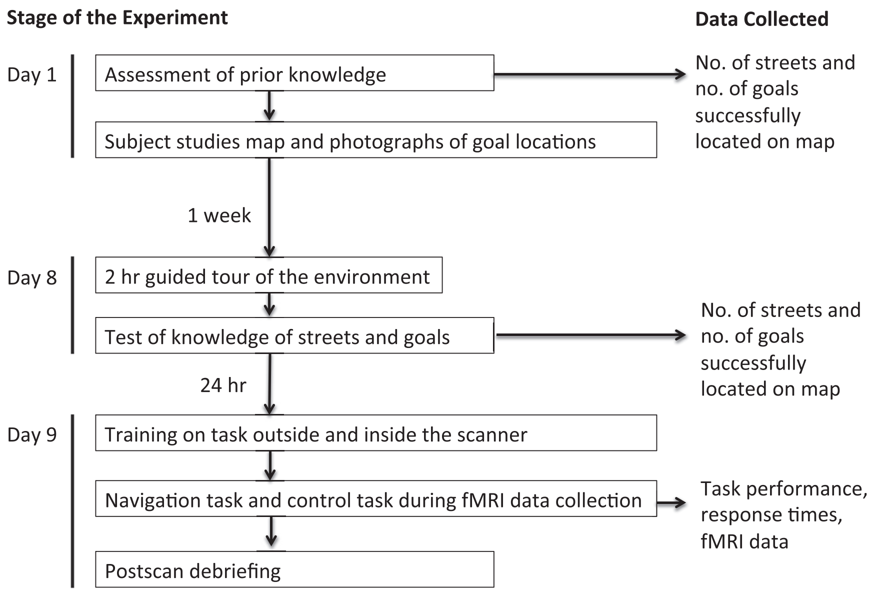

Figure 1. A Flow Chart of the Experimental Protocol

Subjects were instructed to spend at least 30 min studying the training material between days 1 and 8. On day 8, all subjects confirmed that they had completed the training material. See Figure S1 for training materials.

destination (New Goal Event) and asked to indicate via a button press whether they thought the goal was to their left or right. They then viewed footage in which their viewpoint traversed the street (travel period) until arriving near the junction (Figure 2). At this time point, subjects pressed a button to indicate which direction at the upcoming junction provided the shortest path to the goal (Decision Point), after which the movie continued along the route. Varying the distance between the Decision Point and the junction allowed for a temporal jitter (3–9 s) between the Decision Point and outcome (crossing junction). Subjects were told they could not choose to turn around or walk backward at any point. At the beginning of each new street section, subjects were told which street they were on and the direction they were facing (north, south, east, or west). Routes were predetermined such that they generally followed the optimal route but occasionally required a forced detour (Detours) where the movie traveled along a suboptimal path. Subjects were informed that Detours were only temporary obstructions and would not affect the same junction in the future. The goal being navigated to changed several times (four or five) during each route at additional New Goal Events. In control routes (alternating in order with navigation routes), subjects were instructed to not navigate and to avoid thinking about the locations of goals or the directions to them. Control routes had the identical format to navigation routes, except that at New Goal Events, subjects were asked to indicate by a button press whether or not a drink could be purchased from that goal and were instructed which button to press at Decision Points. The button to press at each Decision Point was based on the optimal answer in the navigation version of that route. All routes ended when the current goal was reached and the text “final destination reached” was displayed with a photograph of the goal. Between routes, a gray screen with a fixation cross appeared for 17 s. See Figures 1 and 2 and the Supplemental Experimental Procedures for further details.

Behavioral Results

Subjects acquired a detailed spatial knowledge and accurately performed the tasks (Table S1). For navigation routes, mean accuracy was 84.82% (SD = 10.96) at New Goal Events and 79.91% (SD = 13.28) at Decision Points. For control routes, mean accuracy was 95.90% (SD = 5.77) at New Goal Events and 97.63% (SD = 5.74) at Decision Points. Subjects made significantly fewer errors in the control task ($F_{(1,23)}$ = 40.27, $p < 0.001$). Subjects were both faster to respond and more accurate at Decision Points when the goal was situated closer (in terms of the path distance) and more directly ahead (Table S1). At New Goal Events, we found no relationship between subjects’ performance (accuracy and response time) and the magnitude of the change in any of the spatial parameters (Table S1).

fMRI Results

fMRI analyses revealed that retrosplenial, parietal, and frontal cortical regions and the cerebellum were significantly more active (at an uncorrected threshold of $p < 0.001$) during the navigation task blocks, New Goal Events, and Decision Points than during the control task blocks and events (Figure S2; Table S2). Significantly greater right posterior hippocampal activity was also observed during navigation task blocks than during control task blocks (Table S2).

To gain leverage on the spatial computations performed by the brain during navigation, we probed the fMRI data with measures of the Euclidean distance, path distance, and egocentric direction to the goal. First, we explored our a priori predictions (see Supplemental Experimental Procedures) during New Goal Events, Decision Points, Detours, and Travel Period Events (events sampled during travel periods at the temporal midway point between the time points of the other events, for both navigation and control routes). Second, on finding significant effects, we examined whether similar responses occurred in the control routes. Third, where responses were specific to navigation, we tested whether there was a significantly greater effect in navigation routes than in control routes. Finally, we examined whether these responses were significantly greater during certain event types than others and whether responses were significantly more correlated with one parameter than with others.

Both Euclidean and Path Distances Are Tracked by the Hippocampus during Travel

During Travel Period Events in the navigation routes, activity in the posterior hippocampus was significantly positively correlated with the path distance to the goal (i.e., more active at larger distances, see Figures 3A and 3B; Table S2). However, at the same time points, activity in the anterior hippocampus was significantly positively correlated with the Euclidean distance to the goal (Figures 3A and 3B; Table S2). Significant correlations were also present when we downsampled the Travel Period Events to remove 25% of

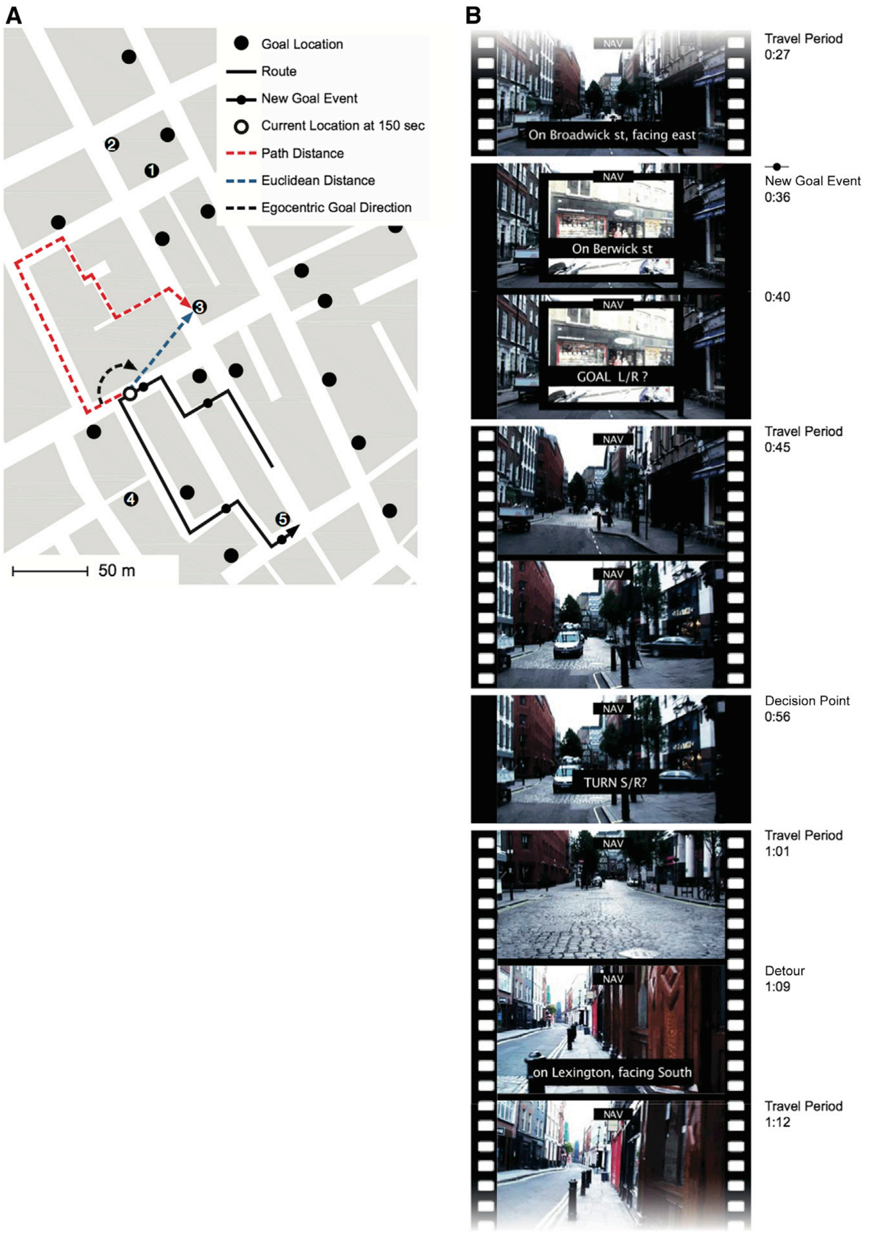

Figure 2. Task
(A) Map of the environment (Soho, London). One of the ten routes is shown (black line) with New Goal Events (black circles on route), and their corresponding goal locations (numbered) are marked. The Euclidean distance (blue dashed line), path distance (red dashed line), and egocentric direction (black dashed line) to the goal are plotted for one location on the route.
(B) An example sequence of movie frames from a small section of one route in the navigation task. At New Goal Events, subjects were given a new goal to navigate to, and they were required to decide whether that new goal was on the left or right in relation to their current facing direction. In between New Goal Events, movies contained footage of travel along the streets (travel periods) and paused near each street junction (Decision Points), where subjects judged which direction provided the shortest route to the goal. On entry to every street (temporally jittered in relation to Decision Points), the street name and cardinal direction were displayed. Occasionally, forced Detours occurred at street junctions where the movie took a suboptimal path to reach the goal. The control task was similar, but no navigational judgments were required. See Figure S2 for comparisons of activity in navigation and control tasks.

the events in which the Euclidean and path distances were most correlated (Table S2). A region-of-interest (ROI)-based analysis of the hippocampal longitudinal axis revealed that whereas the posterior and mid hippocampus were specifically correlated with the path distance to the goal (but not the Euclidean distance), the anterior hippocampus was not specific to the Euclidean distance (Figure 3F; Figure S3). This was further confirmed by direct contrasts between parameters (Table S3).

Models assume that the guidance system is under volitional goal-directed control rather than automatic control. Our data support this view. No significant correlation between hippocampal activity and the distance (either Euclidean or path) to the goal was observed during the Travel Period Events in the control routes. Furthermore, hippocampal activity was also significantly more positively correlated with distance measures in these events during navigation routes than during control routes (Figures 3C–3E; Table S2). Because route (1–5 versus 6–10) and task (navigation versus control) were counterbalanced across subjects, significant correlations could not have been purely stimulus driven. Nor were the correlations with the distance to the goal confounded with the time elapsed or distance traveled since the route began (Table S2).

Beyond the MTL, at a corrected threshold, the anterior cingulate was the only region that showed a significant correlation with distance in any of our event types, specifically (1) during navigation routes and (2) more in navigation routes than in control routes. It was positively correlated with the path distance to the goal during Travel Period Events in navigation routes and significantly more positively correlated in navigation routes than in control routes (Figure S4; Table S2).

Egocentric Goal Direction Is Tracked by the Posterior Parietal Cortex during Travel

Activity in the MTL during travel periods was not correlated with egocentric direction to the goal or the interaction between this directional measure and distance (either Euclidean or path) to the goal. However, consistent with prior observations [10], during navigation routes, activity in the superior posterior parietal cortex was significantly positively correlated with the egocentric direction to the goal (i.e., the greater the angle between the current heading and the heading directly to the goal, the greater the activity [Figures S3 and S4; Table S2]). No such correlation was observed during Travel Period Events in the control routes. However, although the correlation was more positive during

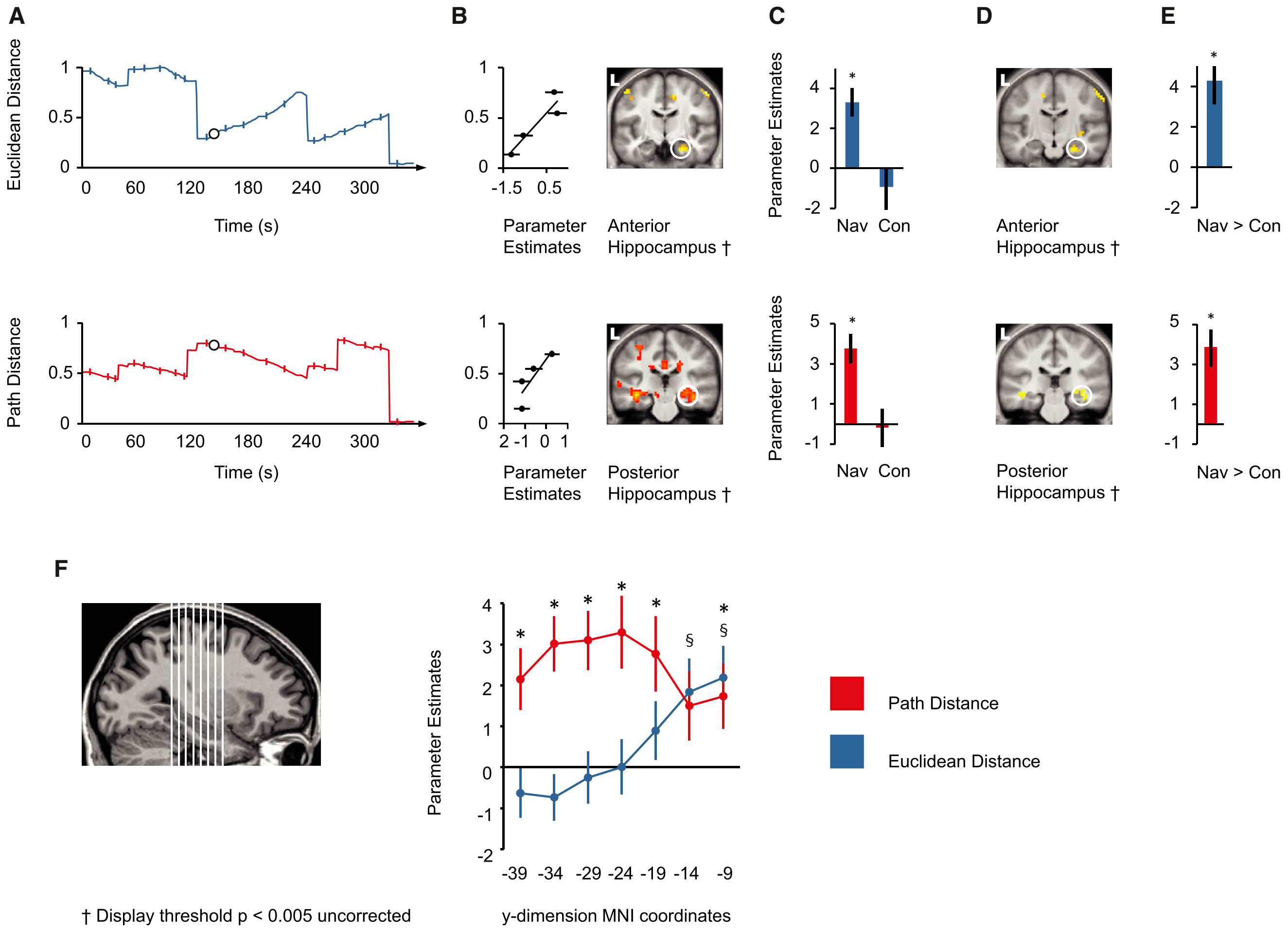

Figure 3. Hippocampal Activity Positively Correlates with Euclidean and Path Distances to the Goal during Travel Periods in Navigation Tasks

(A) Top: the normalized Euclidean distance to the goal is plotted against time for the route shown in Figure 2A. Bottom: the normalized path distance to the goal is plotted against time for the route shown in Figure 2A. Normalization was with respect to the maximum over all routes. On both plots, the circle indicates the time point at 150 s (marked in Figure 2A), and Travel Period Events are indicated with bisecting lines.

(B) Top: right anterior hippocampal activity correlated significantly with the Euclidean distance to the goal during navigation. Bottom: right posterior hippocampal activity correlated significantly with the path distance to the goal during navigation. Accompanying scatter plots show the normalized Euclidean distance (top) and path distance (below; separated into four levels) plotted against parameter estimates at the peak voxel for these regions. Note that these plots were not used for statistical inference (which was carried out within the statistical parametric mapping framework) and are shown solely for illustrative purposes. The following abbreviation is used: L, left.

(C) Top: the parameter estimates for the peak voxel in the right anterior hippocampus in the navigation (Nav) condition are plotted for navigation and control (Con) conditions. Bottom: the parameter estimates for the peak voxel in the right posterior hippocampus in the navigation condition are plotted for the navigation and control conditions. Asterisks indicate significance at a threshold of $p < 0.05$ (family-wise error was corrected for a priori regions of interest). See Figure S3 for parameter estimates in all ROIs.

(D) Top: right anterior hippocampal activity correlated significantly more positively with the Euclidean distance to the goal during navigation conditions than during control conditions. Bottom: right posterior hippocampal activity correlated significantly more positively with the path distance to the goal during navigation conditions than during control conditions. The following abbreviation is used: L, left.

(E) Top: the bar graph shows the parameter estimate for the peak voxel in the right anterior hippocampus in the navigation > control contrast for the Euclidean distance. Bottom: the bar graph shows the parameter estimate for the peak voxel in the right posterior hippocampus in the navigation > control contrast for the path distance. Asterisks indicate significance at a threshold of $p < 0.05$ (family-wise error was corrected for a priori regions of interest).

(F) Left: illustration of the seven sections through the longitudinal axis of the hippocampus. Middle: the parameter estimates of the parametric response to Euclidean and path distances for each of the seven sections (numbers on the x axis indicate the middle MNI y coordinate of each ROI) during Travel Period Events in navigation tasks. These parameter estimates were not used for detecting effects of interest but rather for characterizing the response post hoc. § symbols indicate a significant Euclidean distance, and asterisks indicate a significant path distance in relation to zero at $p < 0.05$ (see Table S5).

Error bars in (B), (C), (E), and (F) denote the SEM.

the Travel Period Events in navigation routes than in control routes, it was not significantly more positive (Table S2). We also observed lateral posterior parietal activity negatively correlated with the egocentric direction to the goal (Figure S4; Table S2); however, this did not survive at corrected thresholds.

Posterior Hippocampal Activity Increases with Proximity and Orientation toward the Goal at Decision Points

Hippocampal activity did not correlate with the Euclidean or path distance at Decision Points. However, because subjects responded faster, and more accurately, when the path distance was shorter and the goal was ahead of them

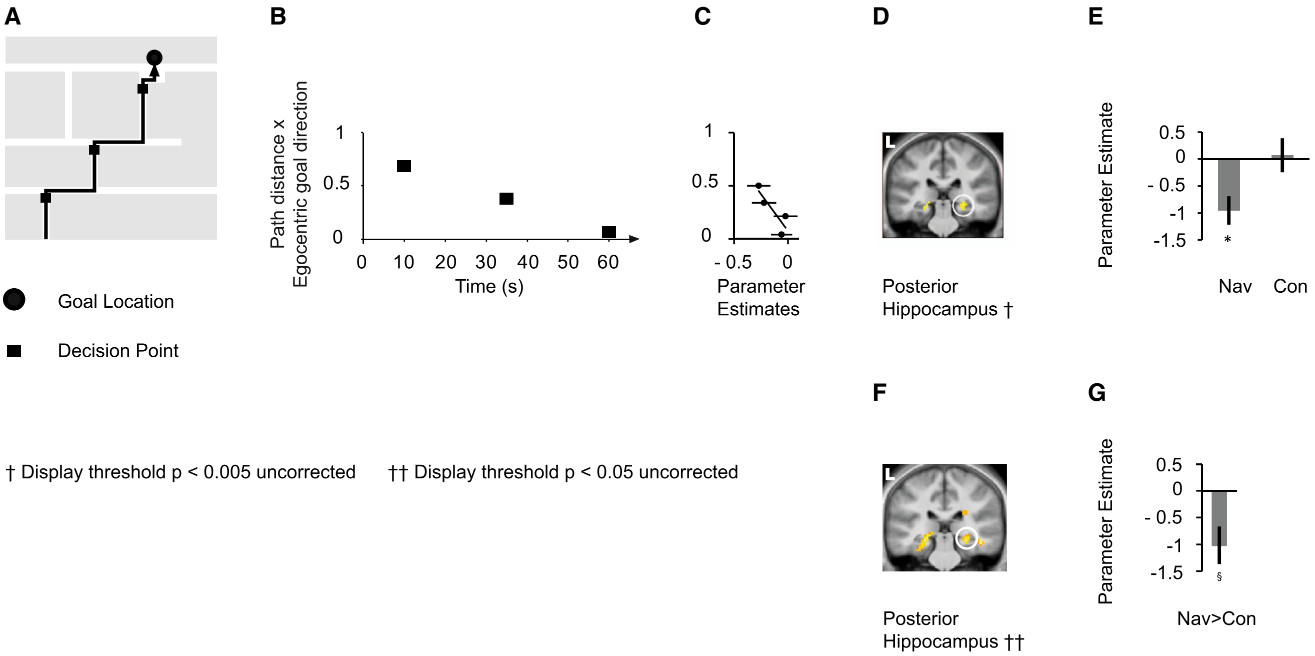

Figure 4. Posterior Hippocampal Activity Negatively Correlates with the Distance and Direction to the Goal during Decision Points in Navigation Tasks

(A) Illustrative map with part of a route (black line) to a goal location (black circle) and Decision Points (black squares).

(B) The parameter "normalized path distance to the goal × egocentric goal direction" (PD×EGD) at the three Decision Points from the example route in (A) is plotted against time.

(C) Normalized PD×EGD separated into four levels is plotted against parameter estimates at the peak voxel of the posterior right hippocampus. Note that the scatter plot was not used for statistical inference (which was carried out within the SPM framework) and is shown solely for illustrative purposes.

(D) Right posterior hippocampal activity correlated significantly negatively with PD×EGD during Decision Points in navigation. The following abbreviation is used: L, left. See Figure S4 for other coronal sections with this and other contrasts.

(E) The parameter estimates for the peak voxel in the right posterior hippocampus in the navigation condition are plotted for navigation (Nav) and control (Con) conditions. Asterisks indicate significance at a threshold of $p < 0.05$ (family-wise error was corrected for a priori regions of interest).

(F) Right posterior hippocampal activity correlated significantly more negatively with PD×EGD during navigation routes than during control routes. The following abbreviation is used: L, left.

(G) The bar graph shows the parameter estimate for the peak voxel in the right posterior hippocampus in the navigation > control contrast for PD×EGD. § symbols indicate significance at a threshold of $p < 0.005$ (uncorrected).

Errors bars in (C), (E), and (G) denote the SEM.

(Table S1), we explored whether hippocampal activity was related to an interaction between the path distance and the egocentric goal direction by examining the response to the multiplication of these two variables (Figure 4). We also included response time in our analysis. We found that posterior hippocampal activity increased the closer, and more directly ahead, the goal lay (Figures 4B–4D; Figures S3 and S4; Table S2). Activity increased such that when subjects were close to and facing the goal, activity was similar to that during the fixation period between routes. No significant correlation with the path distance by egocentric goal direction was observed in the posterior hippocampus in control routes, and the correlation between this parameter and posterior hippocampal activity was significantly more negative in navigation routes than in control routes (Figures 4E–4G; Table S2). The significant correlation in navigation routes was independent of response time, which did not modulate MTL activity. The number of options at Decision Points (two or three) also had no impact on MTL activity (the path distance did not differ between these two types of Decision Points [$t_{(51)} = 0.04$, $p = 0.97$]).

Entorhinal Activity Scales with the Change in the Euclidean Distance at New Goal Events

At New Goal Events, the distance to the goal changed abruptly (Figures 5A and 5C). For navigation routes, we found that the greater the change in the Euclidean distance (but not the path distance) at these time points, the greater the evoked response in the right entorhinal cortex (Figure 5D; Figures S3 and S5; Table S2). At New Goal Events, the goal could move to a location that was closer to or farther from the subject (in terms of both path and Euclidean distances). We found no difference in MTL activity associated with New Goal Events either when the new goal was located closer to the subject or when it was located farther away (for both distance types). Notably, increases and decreases in either the Euclidean or path distance for these two types of New Goal Events were not significantly different in magnitude (Euclidean distance: $t_{(41)} = 0.54$, $p = 0.59$; path distance: $t_{(41)} = 1.96$, $p = 0.056$). No significant correlation with the change in the Euclidean distance was observed in the entorhinal cortex in control routes, and the correlation between entorhinal activity and this parameter was significantly more positive in the New Goal Events in navigation routes than in control routes (Figures 5E–5G; Table S2). The correlation between entorhinal activity and the change in the Euclidean distance during New Goal Events in navigation routes was also significantly more positive than the correlation with the change in the path distance during New Goal Events in navigation routes (Table S3). Finally, we also explored the MTL response to the distance (path and

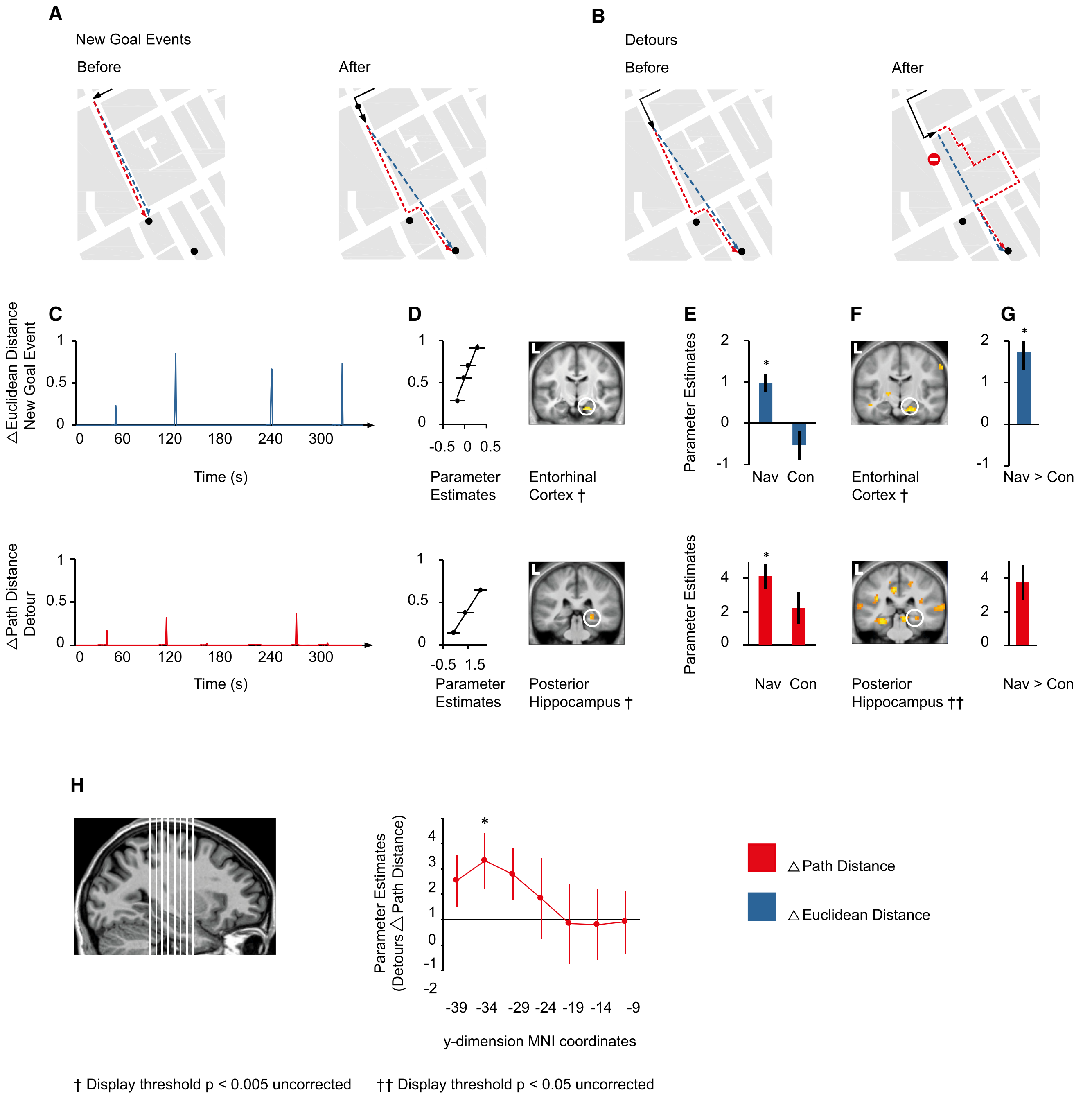

Figure 5. Entorhinal Activity and Posterior Hippocampal Activity Positively Correlate with the Change in the Euclidean Distance to the Goal during New Goal Events and the Change in the Path Distance to the Goal during Detours, Respectively

(A) Illustrative example of how the Euclidean and path distances to the goal can change at New Goal Events.

(B) Illustrative example of how the path distance to the goal can change at Detours. The "no entry" sign marks the Detour, but no marker was presented in the movie.

(C) Top: the normalized differential (Δ) of the Euclidean distance to the goal at New Goal Events is plotted against time for the route shown in Figure 2A. Bottom: the normalized differential (Δ) of the path distance to the goal at Detours is plotted against time for the route shown in Figure 2A. Normalization was with respect to the maximum over all routes.

(D) Top: right entorhinal activity significantly correlated with the Δ Euclidean distance to the goal during New Goal Events in navigation. Bottom: right posterior hippocampal activity significantly correlated with the Δ path distance during Detours in navigation. Accompanying scatter plots show the normalized Δ Euclidean distance (top) and the Δ path distance (bottom) (separated into four and three levels, respectively) plotted against parameter estimates at the peak voxel for these regions. Note that these plots were not used for statistical inference (which was carried out within the SPM framework) and are shown solely for illustrative purposes. See Figure S5 for a display of results on other coronal sections. The following abbreviation is used: L, left.

(E) Top: the parameter estimates for the peak voxel in the entorhinal cortex in the navigation condition are plotted for navigation (Nav) and control (Con) conditions. Bottom: the parameter estimates for the peak voxel in the posterior hippocampus in the navigation condition are plotted for the navigation and control conditions. Asterisks indicate significance at a threshold of $p < 0.05$ (family-wise error was corrected for a priori regions of interest).

(F) Top: right entorhinal activity correlated significantly more positively with the Δ Euclidean distance to the goal at New Goal Events during navigation routes

(legend continued on next page)

Euclidean) to the new goal at New Goal Events and found no significant correlation between MTL activity and either type of distance (Figure S3).

Right Posterior Hippocampal Activity Reflects the Amount of Change in the Path Distance at Detours

At Detours, subjects were unable to proceed along the optimal path and thus had to derive an alternative route to the goal. At these events, the path distance to the goal increased abruptly and by varying amounts (Figures 5B and 5C). Our data show a dissociation between prefrontal and MTL responses at Detours. Consistent with prior studies [6, 29], prefrontal regions, but not MTL regions, were significantly more active at Detours than during optimal route progression at junctions or events in control routes (Figure S2; Table S2). However, we found that right posterior hippocampal, but not prefrontal, activity was positively correlated with the magnitude of change in the path distance during Detours (i.e., Detours that added a large amount of distance evoked more posterior hippocampal activity than did Detours that added a small distance [Figures 5D and 5H; Figures S3 and S5; Table S2]). No equivalent significant correlation was present at corresponding Detour events in the control movies. Although the correlation between the change in the path distance and hippocampal activity at Detours was greater in navigation routes than in control routes, this difference did not reach significance (Figures 5E–5G; Table S2). See Table 1 for a summary of these and other results.

Comparison of Correlations with Spatial Parameters across Different Event Types

We found that all correlations between MTL activity and the distance to the goal were specific to each event type (Table S4). For example, the correlation between posterior hippocampal activity and the path distance during Travel Period Events was significantly more positive during Travel Period Events than during Decision Points or New Goal Events. The posterior parietal response to egocentric goal direction was not significantly more positive during Travel Period Events than during other events (Table S4).

Analysis of the Mean Response in ROIs

When we used an alternative approach of examining the mean response in our ROIs, we found a small number of differences from our statistical parametric mapping (SPM) analysis (Figure S3; Table S6). Examining the Euclidean distance to the goal during Travel Period Events, we found that although there was no significant cluster in the right entorhinal cortex in SPM, our ROI analysis revealed a significant correlation. A similar pattern was found in the left posterior parietal cortex for the egocentric goal direction to the new goal at New Goal Events.

Discussion

Using a novel real-world task, we explored how the brain dynamically encodes the distance to goals during navigation. Our results provide support for both vector- and path-processing accounts of navigational guidance [17–26] and give insight into the precise navigation stages during which the different regions of the MTL process the distance to future goals. In summary, we found that whereas posterior hippocampal activity was related to the path distance to the goal (during travel, decision making, and forced detours), anterior hippocampal activity (during travel) and entorhinal activity (during the processing of new goals) reflected the Euclidean distance to the goal. These responses were relatively specific to these time periods, and with the exception of anterior hippocampal activity, responses were relatively selective to one type of distance.

Our study provides a number of advances over previous fMRI studies exploring representations of distance in the MTL [10, 16, 30, 31]. First, the absence of significant effects in our control routes, and the observation of significantly stronger activity during navigation routes than during control routes in the majority of analyses, indicates that simply being led along a path to a goal is insufficient to engage the MTL in processing the distance. Rather, our data are consistent with the view that distance-to-goal coding requires active navigation based on long-term memory of the environment. Second, while the visual properties of the stimuli and their temporal dynamics might have driven the effects in prior studies [10, 16, 30, 31], we show that this was not the case in our study because task and route were counterbalanced. Finally, the fact that we altered the distance to the goal sporadically at time points (Detours and New Goal Events) along the route shows that the MTL activity correlated with the distance was not simply a function of the time elapsed or distance traveled.

These findings advance our understanding of navigational guidance systems in several ways. Whereas many models propose that the brain processes either the path [24–27] or the Euclidean [17–21] distance component of a vector to the goal, we reveal that both representations are actively deployed during different time windows and by different MTL regions. While it is important to acknowledge that the responses we observed show modulation over time rather than categorical on and off responses, our results are consistent with the following explanation: during the initiation of navigation, when the spatial relationship to the goal must be established, information related to the Euclidean distance along the vector is processed, and when path choice is required at Decision Points or a detour along a new route is required, information related to the path distance is represented. Although such results are consistent with models in which both vector and path search mechanisms are used [23], no current model captures

than during control routes. Bottom: right posterior hippocampal activity correlated more positively with the Δ path distance at Detours during navigation routes than during control routes, but not significantly.

(G) Top: the bar graph shows the parameter estimate for the peak voxel in the right entorhinal cortex in the navigation > control contrast for the Δ Euclidean distance to the goal at New Goal Events. Bottom: the bar graph shows the parameter estimate for the peak voxel in the right posterior hippocampus in the navigation > control contrast for the Δ path distance at Detours. Asterisks indicate significance at a threshold of $p < 0.05$ (family-wise error was corrected for a priori regions of interest).

(H) Left: illustration of seven sections through the longitudinal axis of the hippocampus (these were used for plotting the parameter estimates in the middle panel). Middle: parameter estimates of the parametric response to the Δ path distance at Detours during navigation for each of the seven hippocampal ROIs (numbers on the x axis indicate the middle MNI y coordinate of each ROI). These parameter estimates were not used for detecting effects of interest but rather for characterizing the response post hoc. Asterisks indicate significance relative to zero at $p < 0.05$ (see Table S5).

Error bars in (D), (E), (G), and (H) denote the SEM.

Table 1. Summary of Significant Effects with Parametric Measures in Navigation Routes

	Brain Region			
Event Type	Anterior Hippocampus	Posterior Hippocampus	Entorhinal Cortex	Posterior Parietal Cortex
Travel Period Events	+ ED	+ PD	NS	+ EGD
Decision Points	NS	− PD×EGD	NS	NS
New Goal Events	NS	NS	+ ΔED	NS
Detours	NS	+ ΔPD	NS	NS

Abbreviations are as follows: +, positive correlation; −, negative correlation; Δ, change in the parameter; ED, Euclidean distance; EGD, egocentric goal direction; NS, not significant; and PD, path distance. See Figure S3 for the parameter estimates for each parameter, brain region, and event type and Table S6 for the results of an analysis of the mean response in each ROI.

the dynamic pattern of distance representations we observed. Thus, we provide much needed empirical data for the development of future models.

Previous studies reporting MTL activity correlated with the distance to goal have provided apparently contradictory reports. While some studies have found that activity increases as the goal becomes farther away [10, 31], others have reported that activity increases as the goal becomes closer [16, 30, 32]. These prior studies did not dissect the operational stages during navigation, nor did they isolate the type of distance that might have been represented. By doing so, we found that both profiles of response can occur at different stages of a single journey and that different types of distances can be represented in different time windows. A possible determinant of the activity profile may be whether subjects had to update their spatial position or decide which path to take. In our study, and others [10, 31], activity increased as the distance during periods of spatial updating (e.g., Travel Period Events) became longer. By contrast, in other studies [16, 30], hippocampal activity increased as the distance to the goal became shorter during decision making about which path or direction to take. Our findings extend prior work by revealing that the proximity to the goal along the path (but not the Euclidean) distance, combined with the direction to the goal, modulates hippocampal activity at Decision Points. Previous studies reporting that hippocampal activity increased with proximity to the goal did not include goal direction in their analysis [16, 30]; thus, it is possible that an interaction between distance and direction was present, but not detected. While several models predict that the path to the goal is represented in the hippocampal population activity [22, 24–27] or that activity changes with goal proximity [17, 18, 20], none argue that activity reflects both distance and direction. Given that estimates of the distance along a path have been found to be biased by the number of junctions and turns along the path [33], it is possible that facing away from the goal might increase the subject's internal estimate of the distance. If so, our combined measure of distance and direction may more accurately reflect the subjects' estimate of the distance than the distance we measured from geospatial data. Exploring this will require further research.

While our primary focus was the MTL, we found responses in other regions thought to be important for navigation. Consistent with prior research [5, 11, 16, 34], we observed greater activity in parietal and retrosplenial cortices during navigation tasks (route blocks, New Goal Events, and Decision Points) than during control tasks. Of these regions, the posterior parietal cortex showed a correlation with the egocentric direction to the goal, consistent with a similar previous report [10] and a role in egocentric processing [35]. It is not clear why parietal activity increases the more the goal lies behind the subject. It is possible that landmarks and geometry in the current field of view make it easier to determine the direction to a goal ahead of the subject, and thus by comparison, make it more demanding to track goals located behind. Alternatively, increased parietal activity may suggest that subjects pay greater attention to direction the more the goal lies behind them.

Our results inform the debated specialization of function in the anterior and posterior hippocampus [28, 36, 37]. Posterior hippocampal activity was consistently correlated with the path distance to the goal. This region is the homolog of the rodent hippocampal dorsal (septal) pole, which contains place cells, representing small regions of space with their "place fields" [38], and is thus suited to the fine-grain coding of space along precise paths [28]. Moreover, such cells can exhibit "forward sweeps" during travel [39] and "replay" of locations along the path ahead prior to travel [40], plausibly recruiting more cells the longer the future path, leading to a predicted positive correlation between the length of the path and hippocampal activity. While responses during Travel Period Events and Detours are consistent with this prediction, our response at Decision Points is the opposite of this prediction. Thus, while our data consistently indicate that the posterior hippocampus processes information about the path, it does not appear to do so in a manner directly predicted from "preplay." Greater integration of rodent and human neural recording methods would be useful for gaining traction on this issue.

Our observed anterior hippocampal activity tracking the distance to the goal during travel periods is consistent with a role in spatial updating [13, 31, 41–43]. If human anterior hippocampal cells, like those of rodents [38], have broad spatial tuning, it would make them suited to extracting global environmental information rather than precise paths [28]. Similarly, the spatially extensive repeating grid-like firing of entorhinal grid cells may make them ideal for computing vectors rather than paths [19, 21, 23]. Our observation of a Euclidean-based code in the right entorhinal cortex is consistent with the finding that the same region codes the Euclidean distance to the goal in London taxi drivers navigating a simulation of London [10]. We found that the entorhinal cortex was equally active for increases and decreases in the Euclidean distance, indicating that resetting the distance rather than purely extending it may drive the response. It is possible that the entorhinal cortex is driven by resetting because it may be more computationally demanding to make large alterations in the representation of the distance than to make small changes. Alternatively, another explanation, provided by Morgan et al. [31], is that this response is driven by a repetition-suppression effect. According to this view, the activity is maximal when the change in the distance is large because it provides the least overlap in the regional representation of the distance.

In this study, we separated path and Euclidean distances. Future studies will be required for dissecting the path distance from other variables. Two such variables are "time to reach the goal" and "reward expectation." While our analysis revealed that time elapsed was not correlated with hippocampal activity, it is possible that correlates of the path distance rather than purely the distance relate to the estimated time to the goal. Similarly, because reaching a goal is rewarding and the likelihood of this increases with proximity along the path, the

path distance and reward expectation are related. Manipulating travel speed, travel costs, and reward outcomes may help separate distance, time, and reward expectation. This would help clarify whether the anterior cingulate activity observed to correlate with the path distance is related to reward expectation. Such a prediction is based on evidence that this region processes progress toward goals [44] and the probability of obtaining a reward [45].

Here, we examined navigation in a recently learned environment. In future research, it will be useful to compare how distance is represented in recently learned and remotely learned environments. It is possible that in remotely learned environments, the distance to the goal is represented by cortical regions rather than the hippocampus [46, 47] and that the type of distance represented changes with familiarity of the environment.

Supplemental Information

Supplemental Information contains five figures, six tables, and Supplemental Experimental Procedures and can be found with this article online at http://dx.doi.org/10.1016/j.cub.2014.05.001.

Acknowledgments

All subjects gave informed written consent in accordance with the local research ethics committee. This work was supported by the Wellcome Trust (grant 094850/Z/10/Z to H.J.S.), James S. McDonnell Foundation (H.J.S.), and the Biological and Biotechnical Research Council (L.R.H.). We thank Dishad Husain and Jack Kelley for film production, Fiona Zisch for figure preparation, Martin Chadwick for ROI assistance, and Peter Dayan, Neil Burgess, Eleanor Maguire, Dharshan Kumaran, Caswell Barry, Benedetto de Martino, Kate Jeffery, and four reviewers for their useful comments on the manuscript.

Received: December 9, 2013
Revised: April 8, 2014
Accepted: May 1, 2014
Published: June 5, 2014

References

1. Hafting, T., Fyhn, M., Molden, S., Moser, M.-B., and Moser, E.I. (2005). Microstructure of a spatial map in the entorhinal cortex. Nature *436*, 801–806.
2. O'Keefe, J., and Nadel, L. (1978). The Hippocampus as a Cognitive Map (Oxford: Oxford University Press).
3. Taube, J.S., Muller, R.U., and Ranck, J.B., Jr. (1990). Head-direction cells recorded from the postsubiculum in freely moving rats. I. Description and quantitative analysis. J. Neurosci. *10*, 420–435.
4. Brown, T.I., Ross, R.S., Keller, J.B., Hasselmo, M.E., and Stern, C.E. (2010). Which way was I going? Contextual retrieval supports the disambiguation of well learned overlapping navigational routes. J. Neurosci. *30*, 7414–7422.
5. Hartley, T., Maguire, E.A., Spiers, H.J., and Burgess, N. (2003). The well-worn route and the path less traveled: distinct neural bases of route following and wayfinding in humans. Neuron *37*, 877–888.
6. Iaria, G., Fox, C.J., Chen, J.-K., Petrides, M., and Barton, J.J.S. (2008). Detection of unexpected events during spatial navigation in humans: bottom-up attentional system and neural mechanisms. Eur. J. Neurosci. *27*, 1017–1025.
7. Rauchs, G., Orban, P., Balteau, E., Schmidt, C., Degueldre, C., Luxen, A., Maquet, P., and Peigneux, P. (2008). Partially segregated neural networks for spatial and contextual memory in virtual navigation. Hippocampus *18*, 503–518.
8. Rodriguez, P.F. (2010). Neural decoding of goal locations in spatial navigation in humans with fMRI. Hum. Brain Mapp. *31*, 391–397.
9. Rosenbaum, R.S., Ziegler, M., Winocur, G., Grady, C.L., and Moscovitch, M. (2004). "I have often walked down this street before": fMRI studies on the hippocampus and other structures during mental navigation of an old environment. Hippocampus *14*, 826–835.
10. Spiers, H.J., and Maguire, E.A. (2007). A navigational guidance system in the human brain. Hippocampus *17*, 618–626.
11. Spiers, H.J., and Maguire, E.A. (2006). Thoughts, behaviour, and brain dynamics during navigation in the real world. Neuroimage *31*, 1826–1840.
12. Voermans, N.C., Petersson, K.M., Daudey, L., Weber, B., Van Spaendonck, K.P., Kremer, H.P.H., and Fernández, G. (2004). Interaction between the human hippocampus and the caudate nucleus during route recognition. Neuron *43*, 427–435.
13. Wolbers, T., Wiener, J.M., Mallot, H.A., and Büchel, C. (2007). Differential recruitment of the hippocampus, medial prefrontal cortex, and the human motion complex during path integration in humans. J. Neurosci. *27*, 9408–9416.
14. Xu, J., Evensmoen, H.R., Lehn, H., Pintzka, C.W.S., and Håberg, A.K. (2010). Persistent posterior and transient anterior medial temporal lobe activity during navigation. Neuroimage *52*, 1654–1666.
15. Evensmoen, H.R., Lehn, H., Xu, J., Witter, M.P., Nadel, L., and Håberg, A.K. (2013). The anterior hippocampus supports a coarse, global environmental representation and the posterior hippocampus supports fine-grained, local environmental representations. J. Cogn. Neurosci. *25*, 1908–1925.
16. Sherrill, K.R., Erdem, U.M., Ross, R.S., Brown, T.I., Hasselmo, M.E., and Stern, C.E. (2013). Hippocampus and retrosplenial cortex combine path integration signals for successful navigation. J. Neurosci. *33*, 19304–19313.
17. Bilkey, D.K., and Clearwater, J.M. (2005). The dynamic nature of spatial encoding in the hippocampus. Behav. Neurosci. *119*, 1533–1545.
18. Burgess, N., and O'Keefe, J. (1996). Neuronal computations underlying the firing of place cells and their role in navigation. Hippocampus *6*, 749–762.
19. Kubie, J.L., and Fenton, A.A. (2012). Linear look-ahead in conjunctive cells: an entorhinal mechanism for vector-based navigation. Front. Neural Circuits *6*, 20.
20. Kubie, J.L., and Fenton, A.A. (2009). Heading-vector navigation based on head-direction cells and path integration. Hippocampus *19*, 456–479.
21. Huhn, Z., Somogyvári, Z., Kiss, T., and Erdi, P. (2009). Distance coding strategies based on the entorhinal grid cell system. Neural Netw. *22*, 536–543.
22. Chersi, F., and Pezzulo, G. (2012). Using hippocampal-striatal loops for spatial navigation and goal-directed decision-making. Cogn. Process. *13* (*Suppl 1*), S125–S129.
23. Erdem, U.M., and Hasselmo, M.E. (2014). A biologically inspired hierarchical goal directed navigation model. J. Physiol. Paris *108*, 28–37.
24. Martinet, L.-E., Sheynikhovich, D., Benchenane, K., and Arleo, A. (2011). Spatial learning and action planning in a prefrontal cortical network model. PLoS Comput. Biol. *7*, e1002045.
25. Matsumoto, J., Makino, Y., Miura, H., and Yano, M. (2011). A computational model of the hippocampus that represents environmental structure and goal location, and guides movement. Biol. Cybern. *105*, 139–152.
26. Muller, R.U., Stead, M., and Pach, J. (1996). The hippocampus as a cognitive graph. J. Gen. Physiol. *107*, 663–694.
27. Trullier, O., and Meyer, J.A. (2000). Animat navigation using a cognitive graph. Biol. Cybern. *83*, 271–285.
28. Poppenk, J., Evensmoen, H.R., Moscovitch, M., and Nadel, L. (2013). Long-axis specialization of the human hippocampus. Trends Cogn. Sci. *17*, 230–240.
29. Maguire, E.A., Burgess, N., Donnett, J.G., Frackowiak, R.S., Frith, C.D., and O'Keefe, J. (1998). Knowing where and getting there: a human navigation network. Science *280*, 921–924.
30. Viard, A., Doeller, C.F., Hartley, T., Bird, C.M., and Burgess, N. (2011). Anterior hippocampus and goal-directed spatial decision making. J. Neurosci. *31*, 4613–4621.
31. Morgan, L.K., Macevoy, S.P., Aguirre, G.K., and Epstein, R.A. (2011). Distances between real-world locations are represented in the human hippocampus. J. Neurosci. *31*, 1238–1245.
32. Dupret, D., O'Neill, J., Pleydell-Bouverie, B., and Csicsvari, J. (2010). The reorganization and reactivation of hippocampal maps predict spatial memory performance. Nat. Neurosci. *13*, 995–1002.
33. Thorndyke, P.W. (1981). Distance estimation from cognitive maps. Cognit. Psychol. *13*, 526–550.
34. Iaria, G., Chen, J.-K., Guariglia, C., Ptito, A., and Petrides, M. (2007). Retrosplenial and hippocampal brain regions in human navigation:

complementary functional contributions to the formation and use of cognitive maps. Eur. J. Neurosci. *25*, 890–899.
35. Andersen, R.A., Snyder, L.H., Bradley, D.C., and Xing, J. (1997). Multimodal representation of space in the posterior parietal cortex and its use in planning movements. Annu. Rev. Neurosci. *20*, 303–330.
36. Aggleton, J.P. (2012). Multiple anatomical systems embedded within the primate medial temporal lobe: implications for hippocampal function. Neurosci. Biobehav. Rev. *36*, 1579–1596.
37. Ranganath, C., and Ritchey, M. (2012). Two cortical systems for memory-guided behaviour. Nat. Rev. Neurosci. *13*, 713–726.
38. Jung, M.W., Wiener, S.I., and McNaughton, B.L. (1994). Comparison of spatial firing characteristics of units in dorsal and ventral hippocampus of the rat. J. Neurosci. *14*, 7347–7356.
39. Johnson, A., and Redish, A.D. (2007). Neural ensembles in CA3 transiently encode paths forward of the animal at a decision point. J. Neurosci. *27*, 12176–12189.
40. Pfeiffer, B.E., and Foster, D.J. (2013). Hippocampal place-cell sequences depict future paths to remembered goals. Nature *497*, 74–79.
41. Kumaran, D., and Maguire, E.A. (2007). Match mismatch processes underlie human hippocampal responses to associative novelty. J. Neurosci. *27*, 8517–8524.
42. Howard, L.R., Kumaran, D., Ólafsdóttir, H.F., and Spiers, H.J. (2011). Double dissociation between hippocampal and parahippocampal responses to object-background context and scene novelty. J. Neurosci. *31*, 5253–5261.
43. Duncan, K., Ketz, N., Inati, S.J., and Davachi, L. (2012). Evidence for area CA1 as a match/mismatch detector: a high-resolution fMRI study of the human hippocampus. Hippocampus *22*, 389–398.
44. Shidara, M., and Richmond, B.J. (2002). Anterior cingulate: single neuronal signals related to degree of reward expectancy. Science *296*, 1709–1711.
45. Kennerley, S.W., Walton, M.E., Behrens, T.E.J., Buckley, M.J., and Rushworth, M.F.S. (2006). Optimal decision making and the anterior cingulate cortex. Nat. Neurosci. *9*, 940–947.
46. Hirshhorn, M., Grady, C., Rosenbaum, R.S., Winocur, G., and Moscovitch, M. (2012). Brain regions involved in the retrieval of spatial and episodic details associated with a familiar environment: an fMRI study. Neuropsychologia *50*, 3094–3106.
47. Maguire, E.A., Nannery, R., and Spiers, H.J. (2006). Navigation around London by a taxi driver with bilateral hippocampal lesions. Brain *129*, 2894–2907.

Current Biology, Volume *24*

Supplemental Information

The Hippocampus and Entorhinal Cortex Encode the Path and Euclidean Distances to Goals during Navigation

Lorelei R. Howard, Amir H. Javadi, Yichao Yu, Ravi D. Mill, Laura C. Morrison, Rebecca Knight, Michelle M. Loftus, Laura Staskute, and Hugo J. Spiers

Supplemental Figures

A

a) Name as many of the streets in this area as you can:

1. Oxford St
2. ______________________
3. ______________________
4. ______________________
5. ______________________
6. ______________________
7. ______________________
8. ______________________
9. ______________________
10. ______________________
11. ______________________
12. ______________________
13. ______________________
14. ______________________
15. ______________________
16. ______________________
17. ______________________
18. ______________________
19. ______________________
20. ______________________
21. ______________________
22. ______________________
23. ______________________
24. ______________________
25. ______________________
26. ______________________
27. ______________________
28. ______________________
29. ______________________
30. ______________________
31. ______________________
32. ______________________
33. ______________________
34. ______________________
35. ______________________

Name:______________________

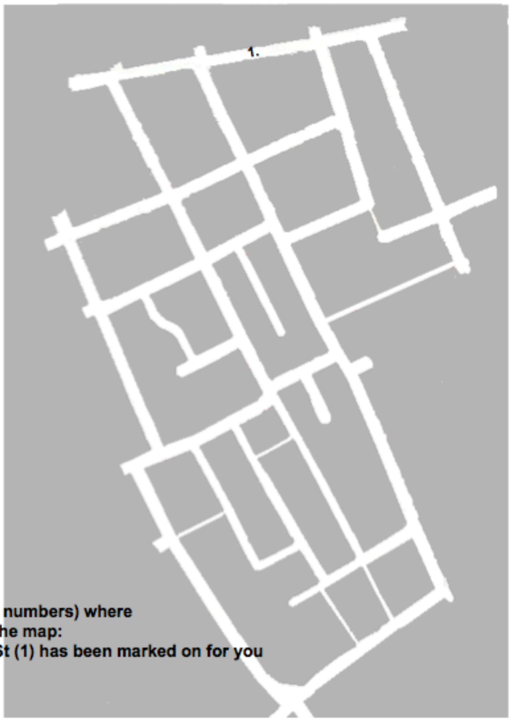

b) Indicate (using the numbers) where these streets are on the map: for example, Oxford St (1) has been marked on for you

a) Please indicate (circle the number) whether any of these places are familiar to you: (i.e., have you walked past them?)

Place

1. 33 Broadwick
2. Calumet Photography
3. Floridita
4. Freedom Bar
5. IMLI Indian Tapas
6. Ingestre Court
7. Let's Fill This Town With Artists
8. Nicholas Wine Shop (Berwick St store)
9. Number One Salon
10. Papaya Cafe
11. Pierre Victoire
12. Refuel
13. Self-Sacrifice
14. Silk Society
15. Sir Tom Baker
16. Sister Ray Records
17. Soho Screening rooms
18. Somerfield (Berwick St store)
19. Star Cafe
20. Tequila Marketing
21. Westminster Kingsway College
22. Yauatcha Restaurant
23. Lo Profile Cafe

b) Indicate (using the dots and numbers) where you think the places you were familiar with are on the map:

Name:______________________

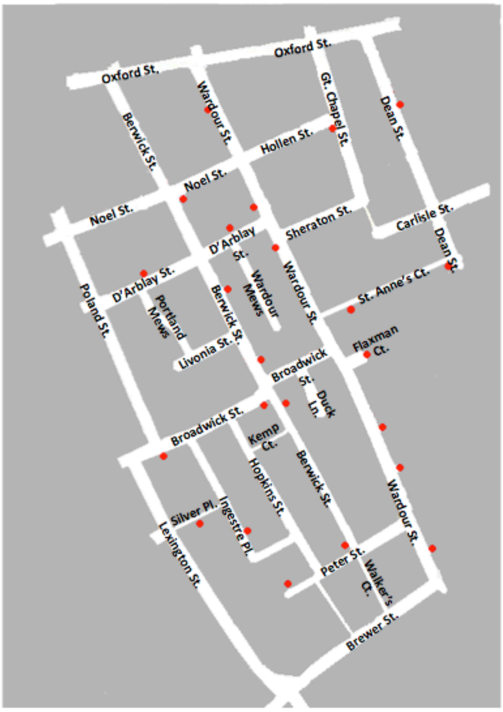

B

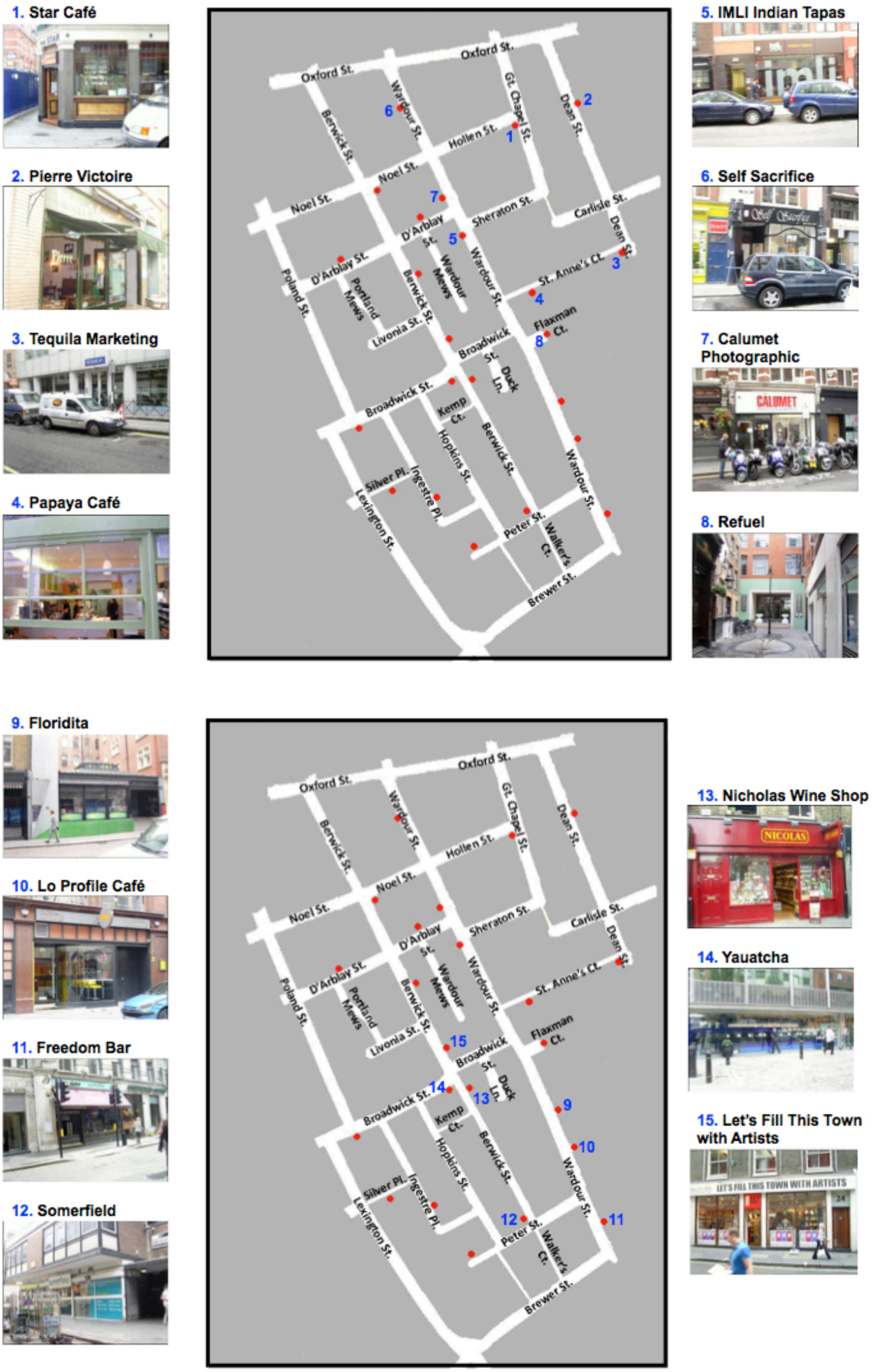
1. Star Café
2. Pierre Victoire
3. Tequila Marketing
4. Papaya Café
5. IMLI Indian Tapas
6. Self Sacrifice
7. Calumet Photographic
8. Refuel
9. Floridita
10. Lo Profile Café
11. Freedom Bar
12. Somerfield
13. Nicholas Wine Shop
14. Yauatcha
15. Let's Fill This Town with Artists
Oxford St.
Wardour St.
Berwick St.
Hollen St.
Gt. Chapel St.
Dean St.
Noel St.
Sheraton St.
Carlisle St.
D'Arblay St.
Poland St.
Portland Mews
Wardour Mews
St. Anne's Ct.
Livonia St.
Flaxman Ct.
Broadwick St.
Duck Ln.
Kemp Ct.
Hopkins St.
Silver Pl.
Ingestre Pl.
Lexington St.
Peter St.
Walker's Ct.
Brewer St.

B (Continued)

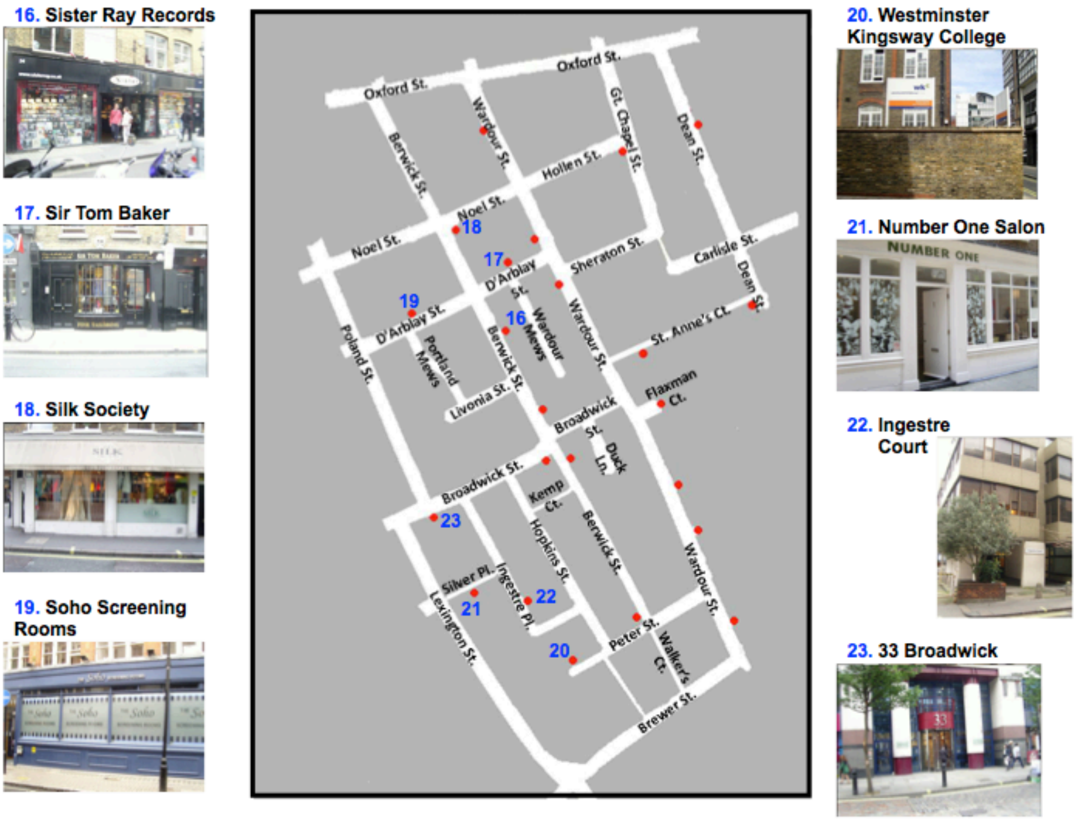

Figure S1. Environmental Knowledge Assessment and Training Material. (**A**) Environmental knowledge assessment. Top: Image from testing material used to test street name knowledge before and after training. Bottom: Image from testing material used to test goal location knowledge before and after training. (**B**) Goal location training material. This material was used to allow subjects to learn about the location of the various goal locations. Note that goal numbers in this material were unrelated to goal location presentation order during New Goal Events during fMRI scanning. Start location material was similar to these but indicated viewpoints at the 10 locations where each route would start.

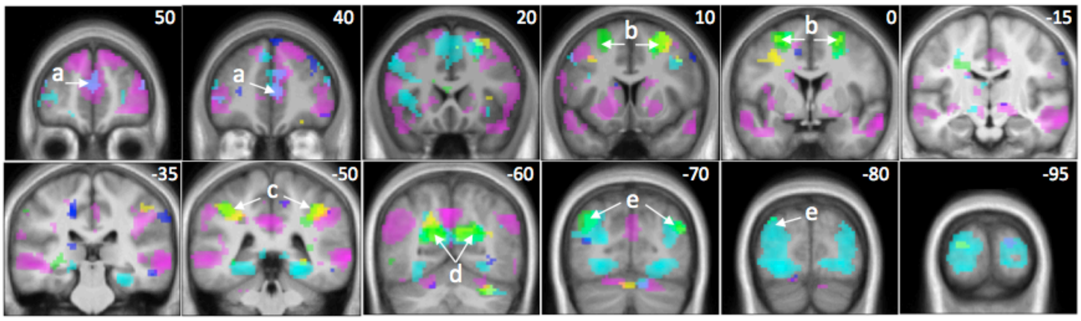

Tasks (Nav > Con)

Travel Period Events (Nav > Con)

Detours (Nav > Con)

New Goal Events (Nav > Con)

Decision Points (Nav > Con)

a overlap of Decision Points + Detours – medial prefrontal cortex

b overlap of Nav > Con Tasks + New Goal Events – superior frontal gyrus

c overlap of Nav > Con Task + New Goal Events – surpamarginal gyrus / intraparietal sulcus

d overlap of Nav > Con Task + New Goal Events + Decision Points – Retrosplenial cortex

e overlap of New Goal Events + Decision Points – Posterior parietal cortex (angular gyrus)

Figure S2. Navigation versus control. Comparison of the navigation condition with the control condition for the task blocks and the four events. The activation maps are displayed on the mean structural image at a threshold of $p < 0.005$ uncorrected, 5 voxels minimum cluster size. Values in the top right corner of each image refer to the MNI y-value for that slice. Slices were chosen to optimally display the pattern of response.

A) Peak Responses

Travel Period Events

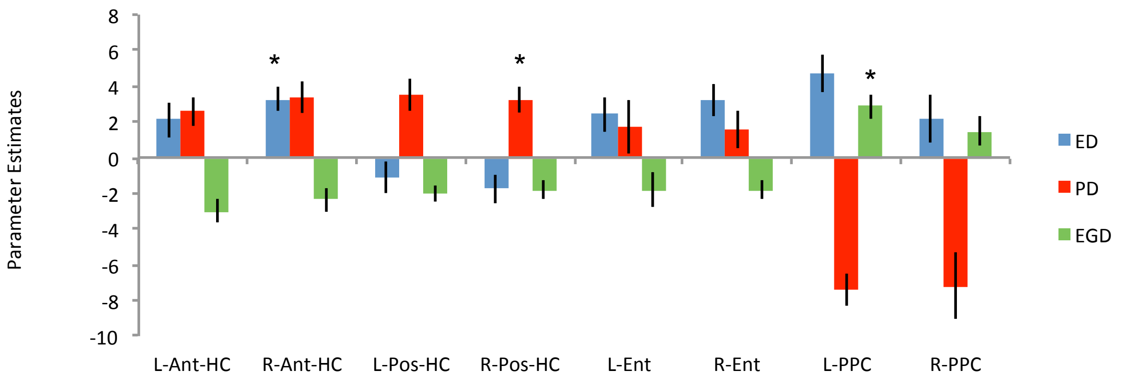

Decision Points

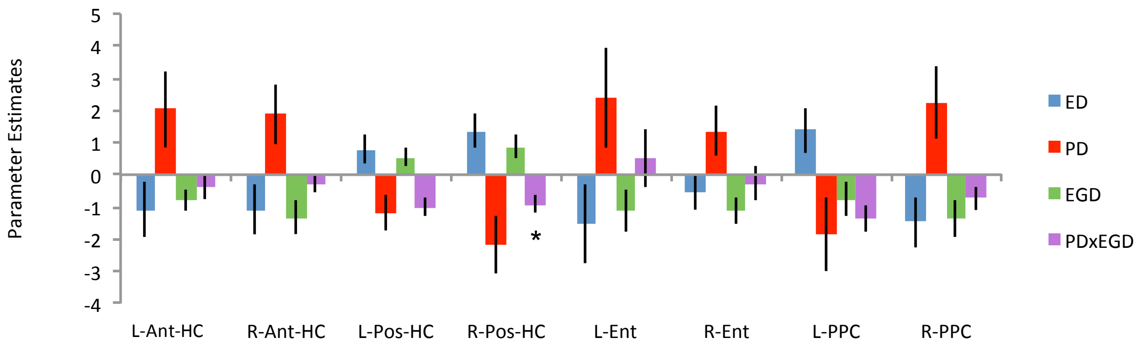

New Goal Events

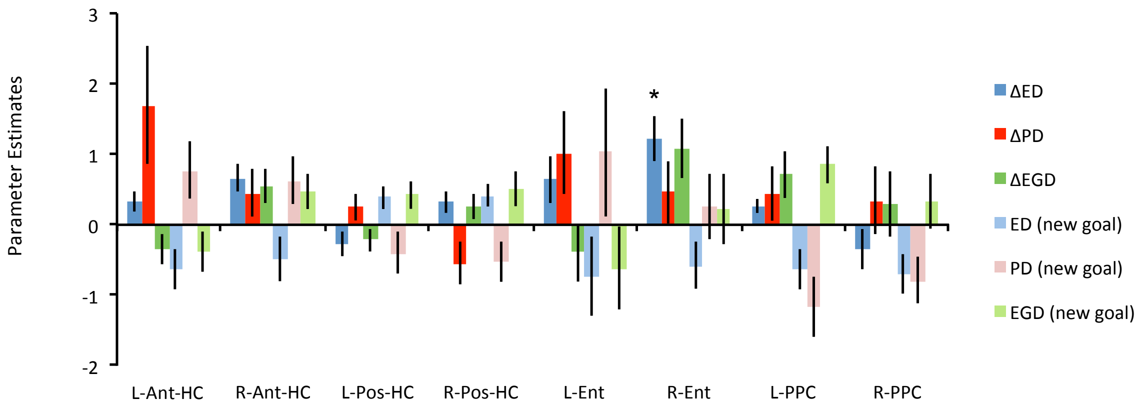

Detours

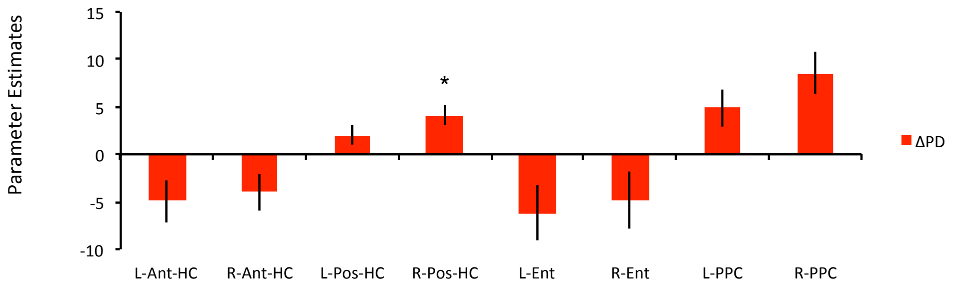

B) Mean Responses

Travel Period Events

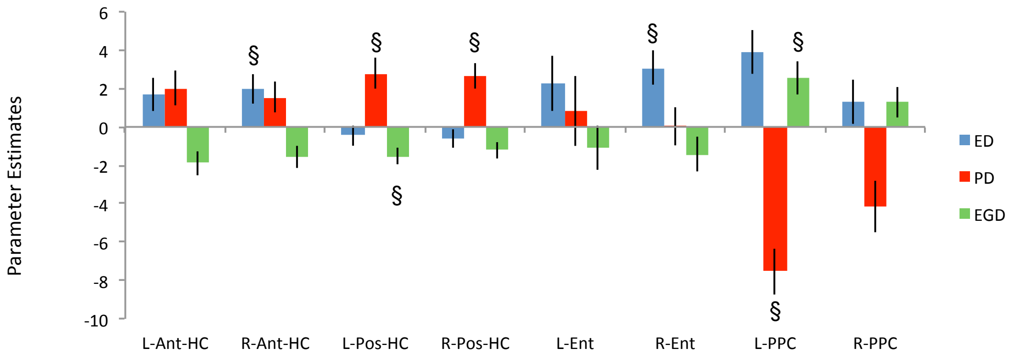

Decision Points

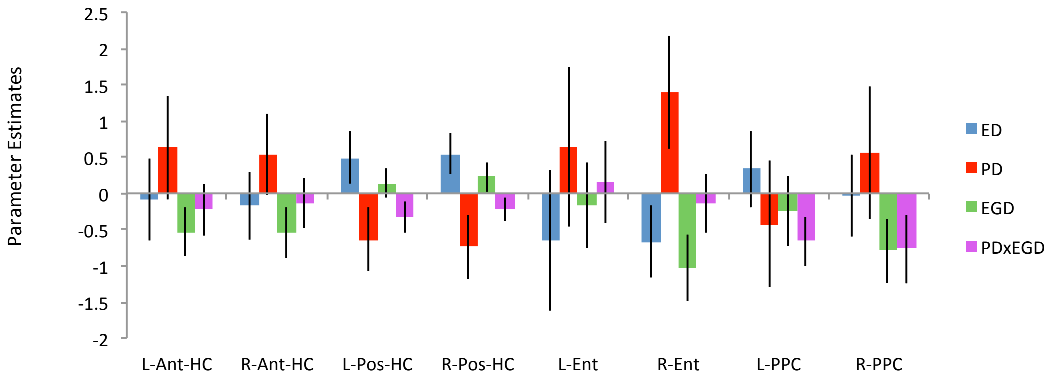

New Goal Events

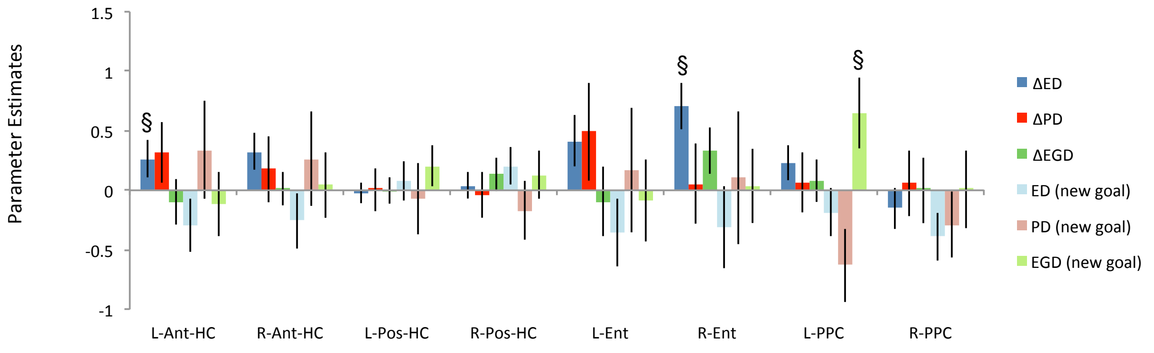

Detours

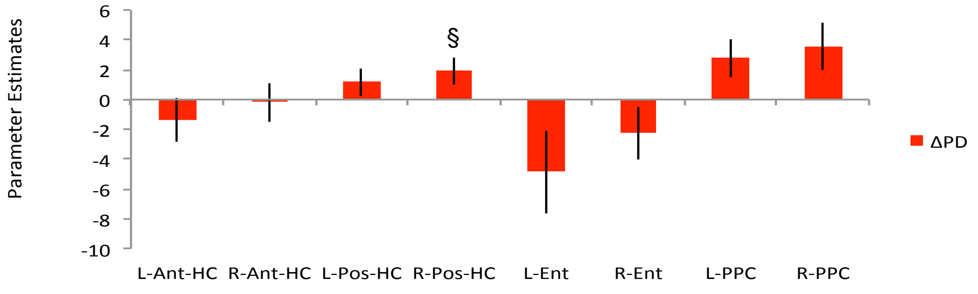

Figure S3. Parameter estimates in each region of interest (ROI) for each spatial parameter for each event type. (**A**) The peak response in each ROI. (**B**) The mean response in each ROI.

ED = Euclidean distance, PD = path distance, EGD = egocentric goal direction. L = Left, R = Right, Ant = anterior, Pos = posterior, HC = hippocampus, Ent = entorhinal cortex, PPC = posterior parietal cortex. Error bars denote SEM. For peak responses in (A) * = significant at $p < 0.05$ corrected for predicted ROIs (right hemisphere for the MTL), see Table S2 for Z-scores derived from SPM. For mean responses in (B) § = significant at $p < 0.05$ for predicted ROIs or $p < 0.05$ bonferroni corrected for other regions, see Table S6 for t-scores and p-values derived from SPSS. See Figures S4 and S5 below for visualisation of the SPM analysis on mean structural images. Note, only regions that showed a significant response and distinct cluster with a minimum of 5 voxels in the ROI were reported in Table S2. There were cases where this criterion was not met, yet a significant mean response in the ROI-based analysis, or a high peak parameter estimate in our ROI, was observed.

Travel Period Events – Positive Contrast

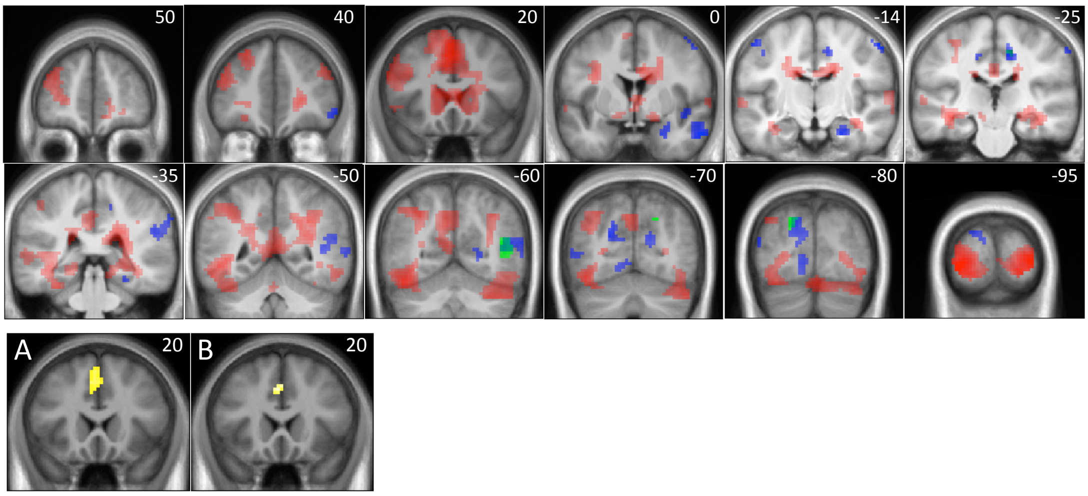

Travel Period Events – Negative Contrast

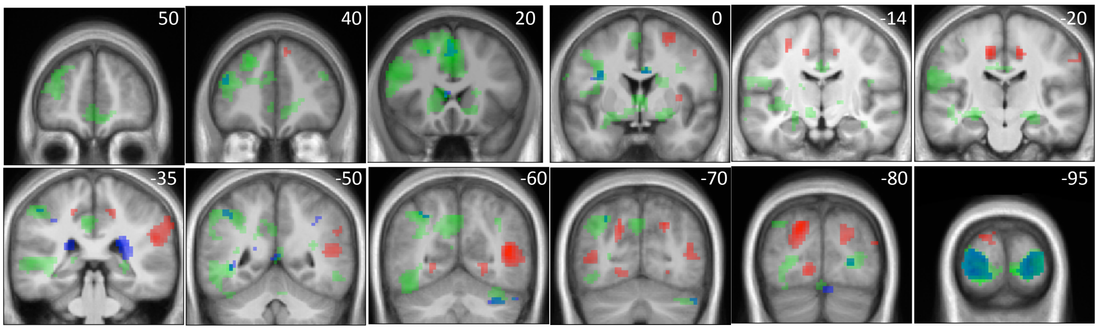

Decision Points - Positive Contrast

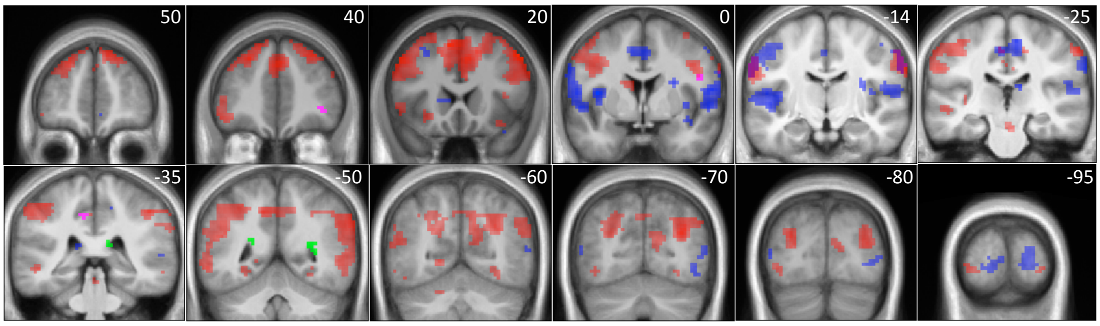

Decision Points - Negative Contrast

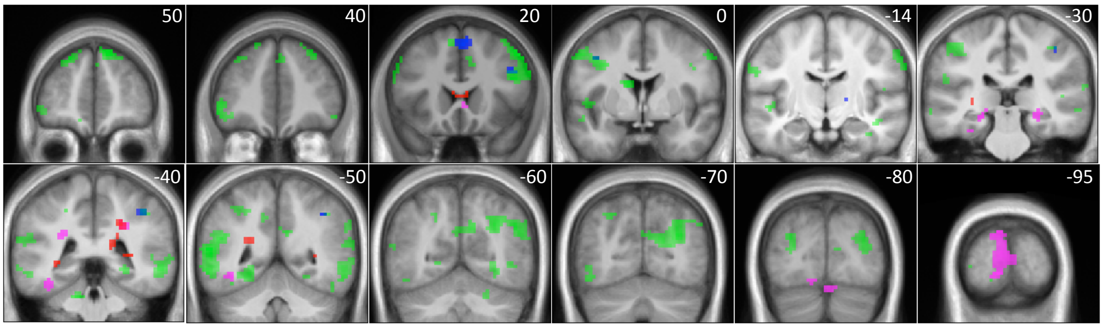

Euclidean Distance ($p < 0.005$ uncorrected)

Path Distance ($p < 0.005$ uncorrected)

Egocentric Goal Direction ($p < 0.005$ uncorrected)

Path Distance x Egocentric Goal Direction ($p < 0.005$ uncorrected)

A. Path Distance $p < 0.05$ corrected for whole brain, B. Path Distance Nav > Con $p < 0.001$ uncorrected

Figure S4 Activity correlated with the spatial parameters at Travel Period Events and Decision Points. The activation maps are displayed on the mean structural, 5 voxels minimum cluster size. Values in the top right corner of each image refer to the MNI y-value for that slice. Slices were chosen to optimally display the pattern of response. Path distance x egocentric distance was only examined at Decision Points. The anterior cingulate activity displayed in images A and B in the section 'Travel Period Events – Positive Contrast' is the only brain region that was both significant at a threshold of $p < 0.05$ (corrected for whole brain volume) for the navigation routes (image A, see Table S2) and also significant at a threshold of $p < 0.001$ uncorrected for Nav > Con for the same parameter and time period (image B: x, y, z, = -3, 20, 37; Z-score = 3.72).

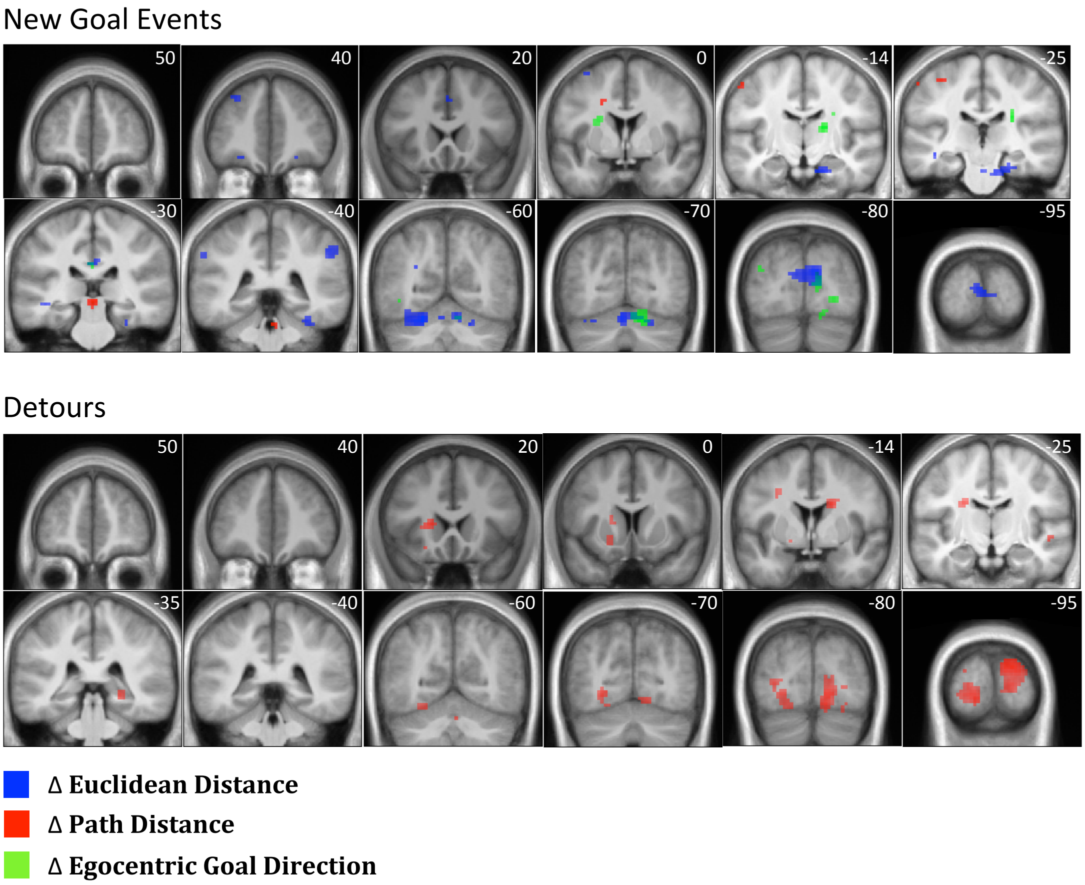

Figure S5 Activity correlated with the change in spatial parameters during New Goal Events and Detours. The activation maps are displayed on the mean structural at a threshold of $p < 0.005$ uncorrected, 5 voxels minimum cluster size. Values in the top right corner of each image refer to the MNI y-value for that slice. Slices were chosen to optimally display the pattern of response. Only path distance changed at Detours.

Supplemental Tables

Table S1. Behavioural Results.

Mean (standard deviation) performance scores (% correct) for the pre- and post-training environmental knowledge assessment

	Pre-training	Post-training
**Streets**	1.44 (3.36)	74.68 (22.55)
**Goals**	1.81 (3.61)	96.92 (6.96)

2x2 repeated measures ANOVA (training phase (pre/post), information type (streets/goals)) revealed a significant main effect of training phase ($F_{(1,23)} = 1092.36$, $p < 0.001$) a significant main effect of information type ($F_{(1,23)} = 23.72$, $p < 0.001$) and a significant interaction ($F_{(1,23)} = 21.00$, $p < 0.001$).

Mean (standard deviation) performance scores (% correct) for New Goal Events and Decision Points in the navigation and control tasks

	Navigation	Control
**New Goal Events**	84.82 (10.96)	95.90 (5.77)
**Decision Points**	79.91 (13.28)	97.63 (5.74)

Mean (standard deviation) reaction times (msec) for New Goal Events and Decision Points in the navigation and control tasks

	Navigation	Control
**New Goal Events**	2036.02 (667.90)	1436.74 (331.71)
**Decision Points**	1902.60 (699.95)	1265.26 (370.63)

Table S1. Behavioural Results (Continued)

Correlation coefficients for behavioural measures and spatial parameters at Decision Points

	Path distance (meters)	Euclidean distance (meters)	Egocentric goal direction (0°- 180°)	Path distance x Egocentric goal direction	Euclidean distance x Egocentric goal direction
**Accuracy**	-0.272*	0.119	-0.342*	-0.367**	-0.117
**Reaction time**	0.363**	-0.099	0.285*	0.409**	0.115

Correlation coefficients for behavioural measures and spatial parameters at New Goal Events

	Change in:	*Change in:*	*Change in:*
	**Path distance (meters)**	**Euclidean distance (meters)**	**Egocentric goal direction (0°- 180°)**
**Accuracy**	-0.121	-0.049	-0.141
**Reaction time**	-0.005	-0.06	0.143

* sig at $p < 0.05$, ** sig at $p < 0.01$. See Results for details of the analysis of the task performance measures.

Table S3. Contrasts between different spatial parameters at the same event type

Brain region	Event type	Parameter comparison	Z-score
Ant. hippocampus	Travel Period Events	ED > PD	1.86
Post. hippocampus	Travel Period Events	ED < PD	3.34*
Post. hippocampus	Decision Points	PDxEGD < ED	2.69 §
Entorhinal Cortex	New Goal Events	ΔED > ΔPD	3.31§

* significant at $p < 0.05$ corrected for region of interest, 5 voxel minimum cluster size. § significant at $p < 0.005$ uncorrected for region of interest, 5 voxel minimum cluster size. Coordinates were very similar to those in the tables above and so are not re-listed here. ED = Euclidean distance, PD = path distance, EGD = egocentric goal direction, Δ = change in the variable, Ant = Anterior, Post = Posterior. In this table '<' and '>' refer to one parameter being significantly more positively, or negatively, correlated relative to the other parameter, not that the absolute correlation of one parameter is greater than the other.

Table S4. Contrasts between the same spatial parameter at different event types

Region	-/+ correlation, Parameter	Event comparison	Z-score
Anterior Hippocampus	+, ED	Travel Period Events > New Goal Events	4.15*
Anterior Hippocampus	+, ED	Travel Period Events > Decision Points	4.04*
Posterior Hippocampus	+, PD	Travel Period Events > New Goal Events	3.00*
Posterior Hippocampus	+, PD	Travel Period Events > Decision Points	3.78*
Posterior Hippocampus	-, PDxEGD	Decision Points > Travel Period Events	3.16*
Posterior Hippocampus	-, PDxEGD	Decision Points > New Goal Events	3.22*
Posterior Hippocampus	+, ΔPD	Detours > New Goal Events	3.26*
Posterior Parietal Cortex	+, EGD	Travel Period Events > New Goal Events	0.33
Posterior Parietal Cortex	+, EGD	Travel Period Events > Decision Points	-0.93

* significant at $p < 0.05$ corrected for region of interest, 5 voxel minimum cluster size. Coordinates were very similar to those in the tables above and so are not re-listed here. ED = Euclidean distance, PD = path distance, EGD = egocentric goal direction, Δ = change in the variable.

Table S5. Analysis of ROI data from the 7 sections through the right hippocampus

MNI-y	Travel<br>ED<br>t (p –value)	Travel<br>PD<br>t (p –value)	Travel<br>ED-PD<br>t (p –value)	Detours<br>ΔPD<br>t (p –value)
-39	-1.05 (0.30)	2.86 (0.010*	-2.22 (0.04*	1.54 (0.14)
-34	-1.30 (0.21)	4.48 (< 0.001)**	-3.75 (0.001)*	2.12 (0.04)*
-29	-0.40 (0.70)	4.31 (< 0.001)**	-2.84 (0.01)*	1.75 (0.09)
-24	0.01 (0.99)	3.71 (0.001)*	-2.39 (0.025)*	0.52 (0.61)
-19	1.25 (0.22)	3.02 (0.01)*	-1.38 (0.18)	-0.09 (0.92)
-14	2.27 (0.01)*	1.79 (0.09)	0.25 (0.81)	-0.15 (0.87)
-9	2.81 (0.01)*	2.19 (0.04)*	0.35 (0.73)	-0.07 (0.95)

ED = Euclidean distance, PD = Path distance, red font indicates a significant response. * significant at $p < 0.05$, MNI-y = MNI y-coordinate values for the middle of the ROI slice through the hippocampus, ** significant at $p < 0.001$ corrected, ED, PD and ΔPD were all independent samples t-tests, n =23, ED-PD comparisons were paired samples t-test, n=23

Table S6. T-scores (uncorrected p-values) from an analysis of ROI mean responses for navigation routes

	L-Ant-HC	R-Ant-HC	L-Pos-HC	R-Pos-HC	L-Ent	R-Ent	L-PPC	R-PPC
**Travel Period Events**								
ED	1.91 (0.068)	2.55 (0.018)§	-0.87 (0.395)	-1.18 (0.250)	1.57 (0.129)	3.51 (0.002)§	3.46 (0.002)	1.14 (0.265)
PD	2.27 (0.033)	1.96 (0.062)	3.54 (0.002)§	4.07 (0.000)§	0.45 (0.655)	0.02 (0.982)	-6.32 (0.000)§	-3.06 (0.006)
EGD	-2.95 (0.007)	-2.56 (0.017)	-3.68 (0.001)§	-2.87 (0.009)	-0.91 (0.371)	-1.62 (0.118)	2.95 (0.007)§	1.63 (0.116)
**Decision Points**								
ED	-0.14 (0.889)	-0.38 (0.711)	1.38 (0.182)	1.87 (0.182)	-0.67 (0.508)	-1.32 (0.200)	0.64 (0.531)	-0.07 (0.946)
PD	0.88 (0.386)	0.96 (0.348)	-1.47 (0.154)	-1.68 (0.154)	0.58 (0.565)	1.80 (0.086)	-0.49 (0.629)	0.61 (0.547)
EGD	-1.58 (0.128)	-1.54 (0.138)	0.69 (0.497)	1.08 (0.497)	-0.29 (0.776)	-2.25 (0.034)	-0.52 (0.607)	-1.77 (0.089)
PDxEGD	-0.65 (0.525)	-0.36 (0.724)	-1.54 (0.138)	-1.45 (0.138)	0.27 (0.790)	-0.36 (0.724)	-1.93 (0.065)	-1.61 (0.121)

Table S6. T-scores (uncorrected p-values) from an analysis of ROI mean responses for navigation routes (Continued)

	L-Ant-HC	R-Ant-HC	L-Pos-HC	R-Pos-HC	L-Ent	R-Ent	L-PPC	R-PPC
**New Goal Events**								
ΔED	1.67 (0.109)	2.14 (0.043)§	-0.23 (0.820)	0.36 (0.719)	1.95 (0.064)	3.58 (0.002)§	1.57 (0.131)	-0.88 (0.389)
ΔPD	1.25 (0.224)	0.64 (0.530)	0.02 (0.985)	-0.22 (0.830)	1.20 (0.243)	0.17 (0.871)	0.27 (0.789)	0.22 (0.830)
ΔEGD	-0.48 (0.634)	0.10 (0.920)	-0.01 (0.989)	0.96 (0.349)	-0.34 (0.739)	1.68 (0.107)	0.46 (0.648)	0.01 (0.995)
ED	-1.30 (0.206)	-1.08 (0.293)	0.47 (0.643)	1.31 (0.202)	-1.27 (0.217)	-0.90 (0.375)	-0.91 (0.372)	-1.92 (0.068)
PD	0.82 (0.422)	0.67 (0.513)	-0.24 (0.816)	-0.69 (0.497)	0.33 (0.745)	0.19 (0.850)	-2.03 (0.054)	-1.06 (0.301)
EGD	-0.42 (0.679)	0.17 (0.868)	1.20 (0.244)	0.62 (0.540)	-0.24 (0.812)	0.11 (0.913)	2.20 (0.038)§	0.02 (0.982)
**Detours**								
PD	-0.92 (0.369)	-0.15 (0.880)	1.25 (0.225)	2.12 (0.045)§	-1.75 (0.094)	-1.29 (0.209)	2.18 (0.040)	2.24 (0.035)

ED = Euclidean distance, PD = Path Distance, EGD = Egocentric Goal Direction, L = Left, R = right, Ant = anterior, Pos = Posterior. Red font indicates significant values. § = $p < 0.05$ for *a priori* regions of interest (see Procedures below) or $p < 0.05$ bonferroni corrected for multiple comparisons with each event type (16 comparisons for Travel Period Events, 21 for Decision Points, 35 for New Goal Events, and 5 for Detours).

Supplemental Experimental Procedures

Subjects

Twenty-four right-handed, healthy volunteers (13 males, mean age = 26.25 years, SD = 3.52 years, range = 20 – 35 years) with normal or corrected to normal vision participated in this experiment. All subjects were free from colour blindness, neurological and psychiatric disease and gave informed written consent in accordance with the local research ethics committee. Only subjects who reported minimal, or no experience, with the environment were invited to take part in the study. Subjects were also screened with the Santa Barbara Sense of Direction Scale [S1]. To avoid testing poor navigators, only those scoring over 3.6 (1 SD below the mean score provided by [S1]) were selected. Subjects tested in our study had a mean score of 4.89 (SD = 0.68).

Test Environment

Soho, London UK, was selected due to its high density of streets (increasing Decision Point sampling) and large number of pubs, clubs, restaurants, cafes and shops, which served as useful landmarks and goals. The region was bounded by Oxford Street in the north, Brewer Street in the south, Lexington Street to the West, and Dean Street to the East. We used a real-world environment rather than a virtual one, to allow subjects the full range of natural sensory cues to encode the space, which has been found to improve spatial memory [S2].

Assessment of Prior Knowledge of the Environment

We assessed subjects' prior knowledge of the environment precisely one week prior to the day of scanning. Subjects were shown a map of the region used with all street names removed, except Oxford Street (Figure S1). They were asked to label as many of the streets as they could. Next, they were shown the same map with street names shown, a set of red dots marked and a list of landmarks (Figure S1). Subjects are asked to indicate if they were familiar with any of the landmarks, and if so to indicate which of the red dots identified its location.

Training

Subjects were required to learn the layout of 26 streets and the location of 23 goals within our test environment in Soho (Figures 1, 2 and S1). The training strategy employed was based on the method London taxi drivers use to learn 'The Knowledge' of London. To ensure that subjects acquired accurate knowledge about the topography of Soho we provided both survey- and ground-level information throughout training. Following completion of the prior knowledge assessment, subjects were given a pack of training materials, this contained: coloured photographs of the 23 goals along with their locations (Figure S1), coloured photographs of 10 start positions along with their locations, a list of 5 routes across the test environment to learn, blank maps for self-testing, and a set of instructions. Subjects were instructed to spend at least 30 minutes looking at each set of photographs with the aim of memorising the location of each goal and start position.

All subjects later confirmed this was the case. Subjects were instructed that they would be expected to remember the name and location of each goal/start solely from presentation of the photograph. To facilitate this process self-testing was encouraged and blank maps, along with examples of the kind of self-testing that might be useful, were enclosed. Finally, to encourage subjects to think about the street layout and devise optimal routes between locations, subjects were required to devise and memorise the optimal (shortest) route between 5 sets of locations (only small sub-sections of these routes overlapped with the routes during scanning). A monetary incentive (£2 extra payment if they scored >70% correct across all tasks) was used to encourage subjects to utilise their training packs as much as possible. Importantly, subjects were specifically told not to use any other maps of Soho to aid their training and instead to rely solely on the materials in this pack.

On the day prior to scanning, subjects were taken on a two-hour tour of the test region in Soho, during which their spatial knowledge was rigorously tested and feedback was given. All subjects were taken on the same training route. This was carefully designed so that each start location was visited once and each goal location was passed at least twice and from different directions. When each of these locations was reached the experimenter showed subjects the coloured photograph of the start or goal as well as their current position on a map. Throughout the tour the experimenter highlighted all useful topographical information and encouraged the subjects to attend to landmarks that would be salient and/or useful for orientation during the subsequent navigation task (i.e., they were highly salient in the movie footage). Subjects were periodically probed about their knowledge of

upcoming streets and goals (e.g. ‘What is the name of the next street coming up on the left?’, ‘There are two goals ahead of us on this road; do you know what they are?’). They were also asked at 6 different locations to indicate, via pointing, the direction along the Euclidean distance to distant goals and describe the optimal route to reach them. None of these goals were later tested from these street segments in the fMRI task. Feedback was provided to the subject for each question asked, using the map where necessary, to ensure subjects benefitted as much as possible from the tour experience. Immediately after the tour, subjects were taken to a café and their post-training knowledge of the test environment was assessed, using the same procedure that had been used pre-training. Immediate feedback was provided to guide subjects towards any aspects they should ‘revise’ on the final evening before scanning. At no point during the training were subjects asked to estimate the path or Euclidean distance.

Stimuli and task

Details of the stimuli are presented in Figure 2. The 10 routes were novel combinations of the streets experienced during the guided tour. Immediately prior to the scan session itself, a training session was conducted to ensure that participants were pre-exposed to both route types and understood the task requirements. A total of 3 training routes were viewed (2 navigation and 1 control). During this period, subjects experienced multiple changes to the goal at several New Goal Events, and thus were aware that their goal would change from time to time during scanning. They were reminded that they must pay attention to the current

goal in order to perform correctly. During scanning, routes were separated by a 17 second interval. During the first 12 seconds of this a centrally positioned white fixation cross was presented on a grey screen and during the latter 5 seconds, just before each route commenced, the fixation cross was replaced with either the cue 'NAVIGATE' or 'CONTROL'; to indicate which type of route would follow. Throughout each route the instruction 'NAV' or 'CON' was displayed at the top of the screen, depending on the route type. The mean duration of the routes was 266.60 sec (SD = 43.63, range = 198 – 325). Routes were presented at walking speed (mean = 1.6 m/s SD = 0.41). The routes were designed to minimize the correlation between Euclidean and path distance to the goal and to maximise the number of turns experienced. Each route was produced in two task formats: Navigation and Control. Route and task were counterbalanced across subjects.

Correlations between the spatial parameters across event types

Correlation Coefficients comparing parameters across all routes

	Euclidean – Path	Euclidean – EGD	Path – EGD
Travel Period Events	0.49*	-0.04	0.06
Decision Points	0.58*	-0.15	0.46*
New Goal Events (change in values)	0.05	-0.24	0.52*

Mean Correlation Coefficients (with range) of the each of the routes

	Euclidean – Path	Euclidean – EGD	Path – EGD
Travel Period Events	0.21 (-0.12 - 0.46)	-0.13 (-0.43 - 0.31)	-0.18 (-0.34 - 0.10)
Decision Points	0.38 (-0.66 - 0.95)	-0.29 (-0.89 - 0.41)	-0.19 (-0.73 - 0.48)
New Goal Events (change in values)	0.24 (-0.56 - 0.79)	-0.08 (-0.64 - 0.63)	0.20 (-0.60 - 0.75)

EGD = Egocentric Goal Direction, * significant at $p < 0.05$

For all routes, the duration that start images remained on screen prior to the display of the first New Goal Event in each route was temporally jittered to last between 5 and 13 seconds. Timing remained constant for New Goal Events and Decision Points. New Goal Events lasted 9 seconds in total, during which the movie was paused. In the initial 4 seconds a colour photograph of the new goal was overlaid, along with text describing its location. For the remaining 5 seconds the photograph of the goal remained but the location description was replaced with the question 'GOAL L/R?'. During this time the subject was expected to make their button press response. Decision Points lasted 5 seconds, during which the movie was paused and the subject was presented with the options to turn at the junction ahead (e.g. 'TURN L/S/R?'), and again, subjects were expected to respond during this time. The amount of time between Decision Points and the onset of the following turn was temporally jittered to last between 3 and 9 seconds to allow separate measures of the BOLD signal at these two events. After each turn, at the beginning of each new street section, text appeared on screen for 3 seconds describing the subject's

current location and general heading direction (e.g., 'Broadwick st, facing east'). At the end of each route, the duration of the final shot of each movie instructing the subject that the final destination had been reached was also temporally jittered so that it remained on screen for between 3 and 9 seconds. In control routes, subjects were instructed which button to press at Decision Points, and had to decide whether it was possible to purchase a drink at the goal location during New Goal Events. Subjects were informed that they could use the street name and direction information presented on entering new streets to orient in control routes, but they must not think about navigating to the goals presented in the New Goal Events.

Immediately after scanning (outside the scanner), subjects completed a debrief interview where all navigation routes viewed during scanning were re-presented in the same order on a laptop (screen size: 12 inch). We do not report data from this debriefing here. Response time and accuracy scores were calculated by comparing the subjects button presses during fMRI scanning with the correct answers based on measurements of the ideal paths and directions to the goal. Statistical analysis of these and all other behavioural data was conducted with SPSS (© IBM Corp).

The ten routes within the test region of Soho were filmed using a HD Sony Z1 and a camera stabilizer (B Hague). Final Cut Pro was used to edit and overlay text onto the original footage to form the first-person-view movie stimuli used in the experiment. Minimizing the correlation was achieved by selecting specific combinations of starting locations, New Goal Events, and Detours. Because of the geometric relationship between these two parameters some degree of correlation

was inevitable, particularly given that at the end of each route the subject reached the goal location and that the goal was not changed too frequently. These constraints were determined to be important from pilot studies. Session 1 started with a navigation route, while session 2 started with a control route. MATLAB 7.5 (© Mathworks) and the Cogent2000 v1.28 Toolbox (http://www.vislab.ucl.ac.uk/cogent_2000.php) were used to control stimulus presentation, interact with the scanner and record response data. All button press responses were made using a button box positioned in the subject's right hand.

Calculation of spatial parameters

Distance and direction data were derived as follows. For each route, the latitude and longitude of start and end points, as well as all those of all the street junctions in between were determined using the program Google Earth (© Google 2010) and converted into Northings and Eastings on a transverse Mercator projection using software from DMAP (©Alan Morton). Each of these coordinates was given a time stamp, indicating the time since the start of that route. MATLAB 7.5 (© Mathworks) was used to provide a linear interpolation over these coordinates to create an estimate of the viewers' spatial position for every second of every route movie. Coordinates of the goal locations were used to create a record of Euclidean distance and egocentric direction to the goal. Euclidean distance measurements were re-scaled across all 10 routes to be between 0 and 1, where a value of 0 corresponded to being at the goal and a value of 1 to being at the maximum Euclidean distance from the goal.

The path distance was the length of the optimal (shortest) route to the current goal. This was measured by summing the length, in meters, of all the component street sections that made up the optimal route. Path distance scores were re-scaled across all 10 routes to be between 0 and 1, where a value of 0 corresponded to being at the goal and a value of 1 to being at the maximum path distance from the goal.

To calculate the egocentric direction to the goal, we first determined the current heading direction (along the route) and the heading direction pointing directly to the goal at each location on each route. The current heading direction was determined by finding the phase angle between current location and the location of the viewer 1 second later on the route. Because there was no future location for each final location, we assumed the viewer was heading in the same direction at the final location as the location occupied 1 second previously. The heading direction pointing directly to the goal was determined by finding the phase angle between the current location and the goal location. The egocentric direction to the goal was the (smallest) angular difference between the current heading direction and the heading direction pointing to the goal. In this study, our main focus was on measuring the overall variation in the egocentric direction toward the goal, thus we collapsed across left and right directions. This meant that values greater than 180° were then subtracted from 360° to bring all values into a range between 0° and 180°. Egocentric direction values were re-scaled across all 10 routes to be between 0 and 1, where a value of 0 corresponded to the goal being directly in front of the subject (0°) and 1 corresponded to the goal being directly behind the subject (180°).

fMRI acquisition and analysis

Participants were scanned at the Birkbeck-UCL Centre for Neuroimaging (BUCNI) using a 1.5 Tesla Siemens Avanto MRI scanner (Siemens Medical Systems, Erlangen, Germany), with a 32-channel head coil. The experimental task, performed over two sessions, lasted around forty-five minutes and twenty-six seconds. A total of nine hundred and forty-one (± 2) functional scans were acquired using a gradient-echo echoplanar imaging (GE-EPI) sequence (TR = 2,897 ms, TE = 50 ms, flip angle = 90°, FoV = $192mm^2$). In each volume thirty-four oblique axial slices, approximately perpendicular to the hippocampus and 3 mm thick were acquired. Following this a high-resolution T1 structural scan was acquired (MPRAGE, 176 slices, 1 x 1 x 1 mm resolution). Foam padding was used to minimise head motions and ear-plugs were used to dampen the noise of the scanner. Stimuli were projected centrally onto a screen at the front of the magnet which participants viewed using a mirror mounted on the head coil (21 x 13 degrees of visual angle of the whole screen). The first 6 functional volumes of each session (dummy scans) were discarded to permit T1 equilibrium. Statistical parametric mapping (SPM8; http://www.fil.ion.ucl.ac.uk/spm/software/spm8/) was used for spatial preprocessing and subsequent analyses. Images were spatially realigned to the first volume of the first session to correct for motion artefacts, coregistered with the structural scan, normalised to a standard EPI template in Montreal Neurological Institute (MNI) space, and spatially smoothed with an isotropic 8 mm FWHM Gaussian kernel filter.

After preprocessing, the smoothed, normalised functional imaging data were entered into a voxel-wise subject-specific general linear model (GLM) (i.e., the first level design matrix). The effects of interest were task epochs (navigation or control) and event-related effects corresponding to: New Goal Events (9 sec duration), Decision Points (5 sec duration), turns along the optimal route (6 sec duration), Detours (6 sec duration) and Travel Period Events (which were time points (zero duration) during the travel periods equidistant between the other events). The number of events varied across subjects because route and task were counter-balanced. Thus, for navigation routes numbers of each type of event were: 21 or 22 New Goal Events, 26 or 27 Decision Points, 12 or 14 Detours, 79 or 80 Travel Period Events. There was the same variation in control route event numbers, e.g. 21 or 22 New Goal Events in control routes. Regressors for each of events/epochs were entered separately for navigation and control routes. For this GLM, the regressors of interest and six subject-specific movement parameters (included as regressors of no interest) derived from the realignment phase of preprocessing, were included. The periods of fixation between blocks was not modelled and treated as the implicit baseline. Each of the regressors of interest was then convolved with the canonical haemodynamic response function (HRF) and a high pass filter with a cut-off of 128 s was used to remove low-frequency drifts. Temporal autocorrelation was modelled using an AR(1) process. At the first level, linear weighted contrasts were used to identify effects of interest, providing contrast images for group effects analysed at the second (random-effects) level. The basic GLM was used to contrast navigation and control tasks and key events as well as Detours with congruent turns (intended route progress).

Following this, in a series of GLM analyses we probed the fMRI data with the spatial parameters (Euclidean distance, path distance, and egocentric goal direction). Parametric regressors were *not* serially orthogonalized, thus allowing each regressor to account independently for the response at each voxel. Separate GLMs were generated to target specific hypotheses about the data at the four event types in our study (Travel Period Events, New Goal Events, Decision Points, and Detours). Each GLM explored the first order parametric modulation of the events of that type, for both navigation and control routes. We did not exclude any New Goal Events or Decision Points from our analysis on the basis of subjects' performance. The table below provides details of the variables assessed in the different GLMs.

General Linear Models with parametric analysis

**Time period**	**Parameters**
Travel Period Events	ED, PD, EGD
Travel Period Events	EDxEGD, PD
Travel Period Events	ED, PDxEGD
Decision Points	ED, PD, EGD, RT
Decision Points	ED, PDxEGD, RT
New Goal Events	ΔED, ΔPD, ΔEGD
New Goal Events	ED, PD, EGD (based on new goal location)
Detours	ΔPD
**Additional models for follow up analyses**	
Travel Period Events (25% events removed)*	ED, PD, EGD
Travel Period Events	ED, PD, EGD, TE

General Linear Models with parametric analysis continued.

**Additional models to allow comparison between events**	
Travel Period Events, Decision Points & New Goal Events	ED, PD, EGD
Travel Period Events, Decision points & New Goal Events	ED, PDxEGD
New Goal Events & Detours	ΔDP

All models contained all the key events (Travel Period Events, New Goal Events, Decision Points, Detours), plus navigation task blocks, control task blocks, non-detour turns. The implicit baseline contained the 17 sec period of fixation between task blocks.

ED = Euclidean distance, PD = path distance, EGD = egocentric goal direction, TE = time elapsed since route started, RT = reaction time, Δ = change in the variable. * 25% events were removed which contained the highest correlation between PD and ED.

In our models exploring Decision Points, we included reaction times (RTs) as a regressor because we observed significant correlations between both path distance and egocentric goal direction with reaction time at Decision Points (Table S1). In light of this behavioural result we examined Decision Points with a model in which the path distance and direction regressors were replaced with a regressor composed of the multiplication of both regressors. We also examined Travel Period Events by applying a similar approach to examine the interaction of the direction with both types of distance. In a further set of GLMs we investigated the parametric effect of the magnitude of the change in path distance at Detours and of the change in all

three spatial parameters at New Goal Events, during both navigation and control routes.

The next set of GLM analyses comprised a variety of control analyses. These aimed to explore whether MTL activity was also modulated by other potential explanatory variables. To summarise, the analyses examined the effect of: 1) time elapsed on the routes, 2) the number of choices at Decision Points, 3) goals moving nearer or further away at New Goal Events, and 4) the impact of removing events from the analysis which contained high correlations between path and Euclidean distance during travel periods. In order to assess the number of choices at Decision Points, the regressor for Decision Points was divided into two regressors, one for events with two choices (T-junctions) the other for events with three choices (crossroads). In the second analysis, two types of New Goal Events (goal moves closer-to vs. goal moves farther-from subjects) were compared in order to determine whether our MTL responses to the change in distance to the goal was driven solely by instances when the goal moves closer-to or farther-from subjects. Two models were constructed for this analysis, one in which the goal moved closer/farther in terms of Euclidean distance, the second for instances where the goal moved closer/farther in terms of path distance. Because hippocampal activity was significantly correlated with both path and Euclidean distance during travel periods, we also examined whether MTL activity was significantly correlated with these parameters by conducting our model of travel periods with the modification that 25% of the events containing the most correlated path and Euclidean distance were removed.

In order to plot parameter estimates for different levels of Euclidean distance to the goal at each event type (e.g. Decision Points) new GLMs were created in which events were assigned to one of four regressors according to the magnitude of their corresponding value. Each regressor reflected 1/4th of the total range of values. Thus, for example when plotting path distance, the first of these regressors reflected events when the subject was nearest to the goal, while the fourth regressor reflected events when the subject was far from the goal. These values were extracted from the sampled events of each participant. Therefore they were not evenly spread over the normalised scale of 0-1. The number of Detours was lower than the rest of the events. Therefore, in order to keep the variance comparable across different event types, path distance values at Detours were split into only three regressors (Figure 5). Note, these GLMs were employed purely for plotting the data and were not used for significance testing.

Given our *a priori* anatomical hypotheses, for distance correlates we specifically report activations in the hippocampus and entorhinal cortex at a threshold of $p < 0.05$ (family-wise-error corrected for brain volume determined by ROIs) and minimum of 5 contiguous voxels. Due to current speculation about the role of the anterior and posterior hippocampus, we used ROIs in the anterior and posterior hippocampus and entorhinal cortex. We focused on the right hemisphere because the right MTL has been more consistently associated with spatial memory in humans (see e.g. [S3–S8]) Both the entorhinal cortex and the hippocampal ROIs were defined using the Duvernoy hippocampal atlas [S9] and Insausti et al. [S10] as guides. Anterior was defined as the most anterior 3rd of the hippocampus, and

posterior as the most posterior 3rd. For follow up contrasts (having initially established a significant response in navigation routes) we explored the MTL data with a more liberal threshold of $p < 0.005$ uncorrected, as we have done in prior work [S11]. To examine the prediction that posterior parietal cortex would encode the egocentric goal direction we used 10 mm spheres located at specific Montreal Neurological Institute (MNI) coordinates [27, -87, 36 and -18, -81, 36] based on [S4]. To plot the response along the longitudinal axis we divided the hippocampus into 7 sections running from the anterior limit to the posterior limit in the right hemisphere: each ROI contained 3 slices of 3mm thickness. This ROI approach was used to display the response along the long axis of the hippocampus. In order to also report left hemisphere MTL parameter estimates we also constructed ROIs for the left anterior hippocampus, left posterior hippocampus and left entorhinal cortex using the same procedure as above. Statistical analyses of mean responses in ROIs were conducted in SPSS, using bonferroni correction to account for multiple comparisons where we had no *a priori* predictions. For completeness, we report all brain regions at a threshold of $p < 0.001$ uncorrected (or $p < 0.005$ for MTL regions) and minimum of 5 contiguous voxels for the planned contrasts in Table S2. We also note which regions survive at a threshold of $p < 0.05$ corrected for whole brain volume. All t-scores and p-values (uncorrected) from our ROI analyses are reported in Tables S5 and S6.

Supplemental References

S1. Hegarty, M., Montello, D. R., Richardson, A. E., Ishikawa, T., and Lovelace, K. (2006). Spatial abilities at different scales: Individual differences in aptitude-test performance and spatial-layout learning. Intelligence *34*, 151–176.

S2. Waller, D., Loomis, J. M., and Haun, D. B. M. (2004). Body-based senses enhance knowledge of directions in large-scale environments. Psychon. Bull. Rev. *11*, 157–163.

S3. Spiers, H. J., Burgess, N., Maguire, E. A., Baxendale, S. A., Hartley, T., Thompson, P. J., and O'Keefe, J. (2001). Unilateral temporal lobectomy patients show lateralized topographical and episodic memory deficits in a virtual town. Brain *124*, 2476–2489.

S4. Spiers, H. J., and Maguire, E. A. (2007). A navigational guidance system in the human brain. Hippocampus *17*, 618–626.

S5. Maguire, E. A., Burgess, N., Donnett, J. G., Frackowiak, R. S., Frith, C. D., and O'Keefe, J. (1998). Knowing where and getting there: a human navigation network. Science *280*, 921–924.

S6. Maguire, E. A., Frackowiak, R. S., and Frith, C. D. (1997). Recalling routes around london: activation of the right hippocampus in taxi drivers. J. Neurosci. Off. J. Soc. Neurosci. *17*, 7103–7110.

S7. Baumann, O., and Mattingley, J. B. (2013). Dissociable representations of environmental size and complexity in the human hippocampus. J. Neurosci. Off. J. Soc. Neurosci. *33*, 10526–10533.

S8. Bohbot, V. D., Kalina, M., Stepankova, K., Spackova, N., Petrides, M., and Nadel, L. (1998). Spatial memory deficits in patients with lesions to the right hippocampus and to the right parahippocampal cortex. Neuropsychologia *36*, 1217–1238.

S9. Duvernoy, H. M. (2005). The Human Hippocampus: Functional Anatomy, Vascularization and Serial Sections with MRI (Springer).

S10. Insausti, R., Juottonen, K., Soininen, H., Insausti, A. M., Partanen, K., Vainio, P., Laakso, M. P., and Pitkänen, A. (1998). MR volumetric analysis of the human entorhinal, perirhinal, and temporopolar cortices. Am. J. Neuroradiol. *19*, 659–671.

S11. Howard, L. R., Kumaran, D., Ólafsdóttir, H. F., and Spiers, H. J. (2011). Double dissociation between hippocampal and parahippocampal responses to object-background context and scene novelty. J. Neurosci. Off. J. Soc. Neurosci. *31*, 5253–5261.
